# Silver-catalyzed remote Csp^3^-H functionalization of aliphatic alcohols

**DOI:** 10.1038/s41467-018-05014-w

**Published:** 2018-07-06

**Authors:** Yuchao Zhu, Kaimeng Huang, Jun Pan, Xu Qiu, Xiao Luo, Qixue Qin, Jialiang Wei, Xiaojin Wen, Lizhi Zhang, Ning Jiao

**Affiliations:** 1 State Key Laboratory of Natural and Biomimetic Drugs, Peking University, 100191 Beijing, China; 2 State Key Laboratory of Organometallic Chemistry, Chinese Academy of Sciences, 200032 Shanghai, China

## Abstract

Aliphatic alcohols are common and bulk chemicals in organic synthesis. The site-selective functionalization of non-activated aliphatic alcohols is attractive but challenging. Herein, we report a silver-catalyzed δ-selective Csp^3^-H bond functionalization of abundant and inexpensive aliphatic alcohols. Valuable oximonitrile substituted alcohols are easily obtained by using well-designed sulphonyl reagents under simple and mild conditions. This protocol realizes the challenging δ-selective C–C bond formation of simple alkanols.

## Abstract (web summary)

Aliphatic alcohols usually require pre-activation for successful functionalization of their carbon chain. Here, the authors report a silver-catalyzed δ-selective functionalization of aliphatic alcohols via Csp^3^-H bond cleavage under mild conditions without the need of substrate pre-activation.

## Introduction

**Fig. 1:**
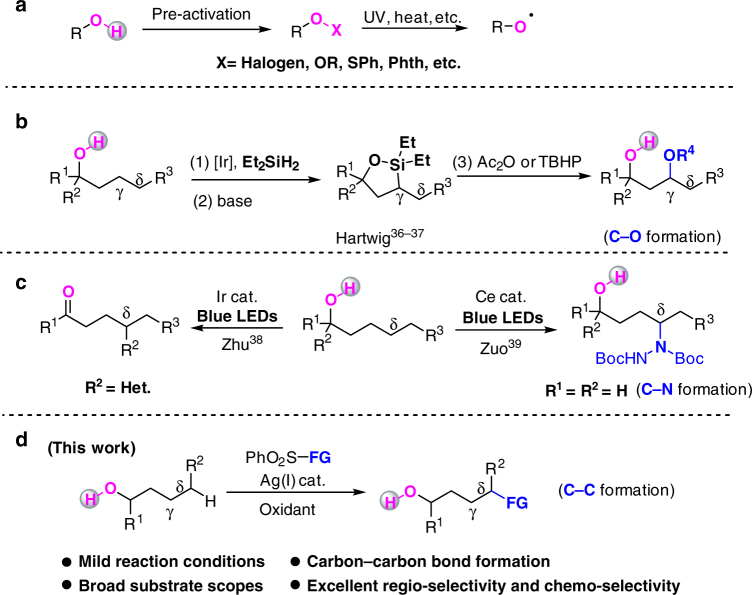
The remote site-selective functionalization reactions of aliphatic alcohols. **a** Traditional indirect strategy to alkoxyl radical. **b** Alcohol-directed γ C–O bond formation. **c** Visible light promoted intra-molecular migration and δ-selective C–N bond formation. **d** This work: Ag-catalyzed direct δ-selective C–C bond functionalization

Aliphatic alcohols that are readily availble and bulk chemicals serve as important building blocks for the construction of value-added molecules for organic chemists^1,2^. However, the selective modification or functionalization of aliphatic alcohols on the carbon chain is very hard due to the inertness of Csp^3^-H bonds as well as the difficulties in the control of regioselectivity, and therefore remains a challenging issue^3–6^. Since the original discoveries by Barton, the remote functionalization via 1,5-Hydrogen Atom Transfer (1,5-HAT) of alkoxyl radicals has been frequently applied in different transformations. However, these methods require a pre-activation of alcohols and the corresponding precursors, such as nitrite esters^7–9^, peroxy compounds^10–13^, hypohalites^14–18^, N-alkoxyphthalimides^19–21^, N-alkoxylpyridine-2-thiones^22,23^, and lead(IV) alkoxides^24,25^, are sometimes hard to handle or prepare and usually need harsh conditions for the genaration of alkoxyl radicals (Fig. 1a). Strategies for the direct activation of simple alcohols under mild conditions are highly desirable.

The direct generation of alkoxyl radicals from alcohols is attractive but more challenging as the bond dissociation energy of O–H bond is around 105 kcal mol^–1^^26^. To address this problem, transition metal catalysis or photocatalysis were developed inducing β-scission reactions of tertiary alcohols^27–35^. Hartwig and coworkers pioneeringly reported a direct γ-selective C–O bond formation of alcohols with the assistance of silicon reagents by Ir-catalysis (Fig. 1b)^36,37^. Zhu and coworkers developed a novel intra-molecular heteroaryl migration of tertiary alcohols by photocatalysis^38^. With a cerium-based photocatalyst, Zuo and coworkers reported an elegant δ-selective C–N bond formation reaction of primary alcohols enabled by a ligand-to-metal charge transfer excitation (Fig. 1c)^39^. To the best of our knowledge, the direct and selective inter-molecular C–C bond formation of simple alcohols via distal Csp^3^-H activation to afford corresponding oxime ether products is still unknown.

Herein, we describe a silver-catalyzed direct δ-selective Csp^3^-H bond functionalization of simple and readily available alkanols under mild conditions (Fig. 1d). This reaction features the three-fold advantages: without the need of pre-activation, a simple Ag/oxidant system enables this selective Csp^3^-H functionalization of aliphatic alkanols; inert C–H bond activation with excellent regio-selectivities and chemo-selectivities are achived; the mild reaction conditions, broad substrate scope, and potential for further applications make this method attractive for the synthesis of valuable functionalized alcohols.

## Results

### Initial optimization of the reaction conditions

Oximes and oxime ethers have various synthetic applications and exist in multiple bioactive molecules^40,41^. In recent years, the well-designed sulfonyl oxime ether reagents showed great reactivity in alkyl radical chemistry^42–46^. Inspired by the recently developed silver-catalyzed radical reactions^47–60^ and our continues interest in developing radical reactions with sulfonyl reagents^58,59^, we envisioned that the sequence silver-catalyzed radical process would achieve the challenging C–H bond functionalization. To investigate our hypothesis, we initially chose **A** as the radical acceptor for the direct functionalization of the widely existed 1-octanol (**1a**). We tested several oxidants with AgNO_3_ (20 mol%) in CH_3_CN/H_2_O (1:1) under argon atmosphere at 50 °C (Table 1, entries 1–4). To our delight, when Na_2_S_2_O_8_ was used as the oxidant, the oxime ether product **2a** was obtained in 37% yield (Table 1, entry 3). K_2_S_2_O_8_ was a bit more efficient compared with Na_2_S_2_O_8_ (40%, Table 1, entry 4). The yield of **2a** based on the recovered starting materials increased to 71% when acetone/H_2_O (1:1) was employed as solvents (Table 1, entry 5). The reaction is unable to carry out without silver catalyst (Table 1, entry 6). Moreover, the solubility of silver salts is vital in this reaction as the insoluble AgI was inefficient (Table 1, entry 7). Other metal catalysts, including CuCl_2_, FeCl_2_, and MnBr_2_ did not show efficiency (Table 1, entries 8–10). Other solvents such as PhCF_3_/H_2_O or DMSO/H_2_O were inactive (Table 1, entries 11–12). Furthermore, the substituted aryl sulfonyl reagent (**B**, **C**) as well as the alkyl sulfonyl reagent **D** are not more effective compared with reagent **A** (Table 1, entries 13–15).

**Table 1:** Optimization of reaction conditions


Entry	Cat (20 mol%)	Reagent	Oxidant	Solvent (1 mL/1 mL)	Yield (%)^a^
1	AgNO_3_	**A**	PIDA	CH_3_CN/H_2_O	Trace
2	AgNO_3_	**A**	Oxone	CH_3_CN/H_2_O	Trace
3	AgNO_3_	**A**	Na_2_S_2_O_8_	CH_3_CN/H_2_O	37
4	AgNO_3_	**A**	K_2_S_2_O_8_	CH_3_CN/H_2_O	40
5	AgNO_3_	**A**	K_2_S_2_O_8_	Acetone/H_2_O	55(71)^b^
6	–	**A**	K_2_S_2_O_8_	Acetone/H_2_O	n.d.
7	AgI	**A**	K_2_S_2_O_8_	Acetone/H_2_O	Trace
8	CuCl_2_	**A**	K_2_S_2_O_8_	Acetone/H_2_O	Trace
9	FeCl_2_	**A**	K_2_S_2_O_8_	Acetone/H_2_O	Trace
10	MnBr_2_	**A**	K_2_S_2_O_8_	Acetone/H_2_O	Trace
11	AgNO_3_	**A**	K_2_S_2_O_8_	PhCF_3_/H_2_O	n.d.
12	AgNO_3_	**A**	K_2_S_2_O_8_	DMSO/H_2_O	Trace
13	AgNO_3_	**B**	K_2_S_2_O_8_	Acetone/H_2_O	32
14	AgNO_3_	**C**	K_2_S_2_O_8_	Acetone/H_2_O	54
15	AgNO_3_	**D**	K_2_S_2_O_8_	Acetone/H_2_O	37

Reaction conditions: **1a** (0.2 mmol), catalyst (0.04 mmol), oxidant (0.3 mmol), reagent (0.4 mmol), solvent (2 mL), stirred at 50 °C under Ar (1 atm) for 24 h

^a^Isolated yield

^b^Yield based on recovered alcohols

### The δ-selective functionalization of primary alkanols

With the optimized reaction conditions in hand, we started to investigate the substrate scope of this transformation. The frequently used alkanols (**1a**–**b**) reacted well to afford the corresponding oxime ether products in moderate yields (**2a**–**b**). Moreover, multiple function groups such as the halogen and azido groups are tolerated (**2c**–**e**). To our delight, better yields are obtained in the reaction of alkanols bearing an oxygen atom at the ε position, owing to the higher stability and stronger nucleophilicity of α-oxygen carbon-centered radicals than typical alkyl radicals (**2f**–**i**).

Notebaly, when we explored the aryl group substituted alkanols, the active benzylic C–H bond remained untouched in this transformation (**2j**–**l**), highlighting the excellent regio-selectivity and chemo-selectivity of the present transfrmation. Furthermore, the five-membered, six-membered, and the four-membered ring substituted alcohols reacted well to afford the corresponding oxime ethers with 51–64% yields (**2m**–**p**).

Despite the secondary carbon–hydrogen functionalization, the tertiary carbon–hydrogen is also compatible in this protocol. The alcohol **1q** containing a tertiary carbon–hydrogen bond afforded **2q** in a little bit low yield. We suppose that the steric hindrance of **1q** blocked the radical addition process of carbon radical to sulfonyl reagent (Table 2).

**Table 2:** AgNO_3_-catalyzed δ-selective functionalization of primary alkanols

Standard conditions: see entry 5, Table 1. Yields shown are isolated products

^a^Yield based on recovered alcohols

^b^Determined by ^1^H NMR

^c^Acetone/H_2_O (0.6 mL/0.6 mL) was used

### The δ-selective functionalization of substituted alkanols

With the aforementioned results in hand, we next explored more special alkanols under the optimized conditions (Table 3). The 2-butoxyethanol and 2-ethoxyethanol reacted well to afford **2r** and **2s** in moderate yields, respectively. The 2-methoxyethanol derivative **2t** could be isolated in 33% yield, as the primary alkyl radical is unstable compared to secondary alkyl radicals. Besides the primary alkanols, the secondary alcohol **1u** is compatible in our conditions producing **2u** with 2:1 of diastereoisomers. Moreover, the γ-substituted alcohol **1v** afforded **2v** in moderate yield. Unfortunately, the β-substituted alcohol **1w** was inactive because of the gem-dimethyl effect (**2w**).

**Table 3:** AgNO_3_-catalyzed δ-selective functionalization of substituted alkanols

Standard conditions: see entry 5, Table 1. Yields shown are isolated products

^a^Diastereoselectivity was determined by ^1^H NMR

^b^Acetone/H_2_O (0.6 mL/0.6 mL) was used

^c^Yield based on recovered alcohols

### Further application of functionalized alkanols

**Fig. 2:**
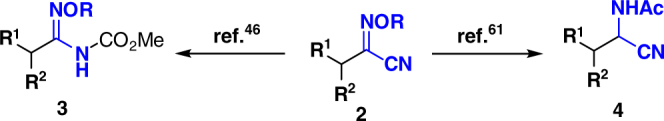
The chemoselective transformation of **2**. The oxime ether product **2** could be converted to valuable amidoxime product **3** and α-cyanoamine product **4**

The oximonitrile fragment in the functionalized products is an important precursor of amidoxime which are key motifs of some fungicides, insecticides, and other bioactive compounds (Fig. 2)^46^. By a zinc-mediated reduction, the oxime can transform into the corresponding amine product **4** (Fig. 2)^61^. Through further operation, **4** could transform into tetrazoles, oxadiazoles, and even amino acid derivatives, which are valuable motifs in bioactive molecules and drugs^62,63^.

### Mechanistic studies

**Fig. 3:**
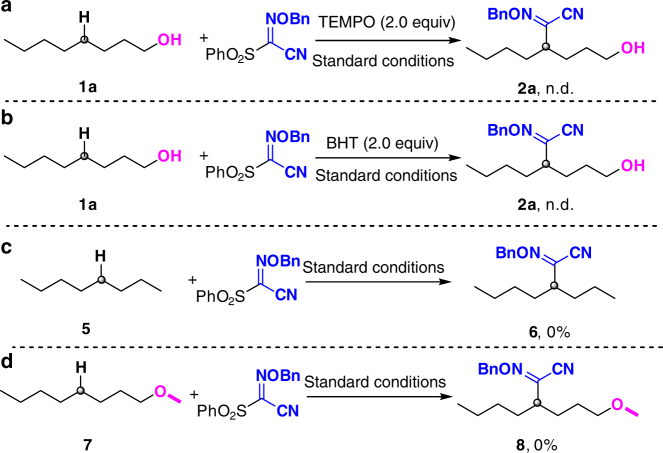
The mechanistic studies. **a** Radical scavenger experiment with TEMPO. **b** Radical scavenger experiment with BHT. **c** Reaction of *n*-octane under standard conditions. **d** Reaction of protected alcohol under standard conditions

Several experiments were investigated to gain the mechanistic insight of this transformation. When stoichiometric amount of TEMPO (Fig. 3a) or BHT (Fig. 3b) was added to the reaction mixture under standard conditions, respectively, no oxime ether product was detected with the revovery of the substrate **1a**. These results indicated that this silver-catalyzed transformation may undergo a radical pathway. Then, we studied the effect of the hydroxyl group. The 1-Octane **5** cannot transform to the corresponding selective functionalized product **6** under standard reaction conditions (Fig. 3c). Furthermore, the reaction of 1-methoxyoctane **7** and reagent **A** afforded no oxime ether product **8** (Fig. 3d). These results supported that the hydroxyl group in the alkanols is essential for the initiation of this transformation.

**Fig. 4:**
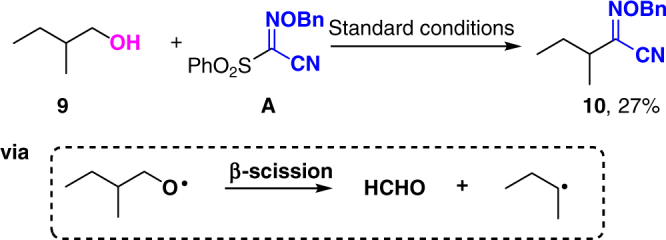
The β-scission experiment. Under standard conditions, N-(benzyloxy)-2-methylbutanimidoyl cyanide **10** could be prepared from **9** through an alkoxyl radical-induced β-scission process

To be emphasized, when 2-methyl-1-butanol **9** was tested, N-(benzyloxy)-2-methylbutanimidoyl cyanide **10** was obtained in 27% yield (Fig. 4). This transformation involves an alkoxyl radical-induced β-scission process^20^, which alternatively supports the direct alkoxyl radical generation from alcohols enabled by the current silver/oxidant catalysis.

**Fig. 5:**
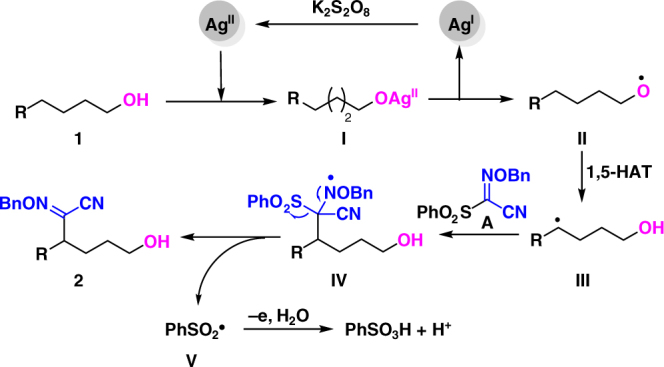
Proposed mechanism. The proposed mechanism involves a Ag^I^/Ag^II^ catalytic cycle

Based on the aforementioned experimental results, the possible mechanism was proposed in Fig. 5. Initially, AgNO_3_ is oxidized to Ag^II^. Then, intermediate **I** formed by coordination of alcohols **1** to Ag^II^ undergoes a homolytic cleavage process to afford alkoxyl radical **II** and regenarate Ag^I^. Subsequently, the intermediate **II** undergoes 1,5-HAT to afford carbon radical **III** which is then trapped by sulphonyl reagent **A** to afford radical intermediate **IV**. The followed fragmentation produces δ-selective functionalized alkanol **2** with the release of sulponyl radical **V**. Finally, the sulfonyl radical is transformed to benzenesulfonic acid^51^.

## Discussion

In summary, we have developed a AgNO_3_-catalyzed δ-selective functionalization of aliphatic alcohols via Csp^3^-H bond cleavage under mild conditions without pre-activation of the alcohol substrates. This atom-economical and easy handled strategy has been applied to various primary and secondary alkanols affording valuable oximonitrile substituted products with high chemo-selectivity. Mechanistic studies indicate the reaction undergoes an alkoxyl radical-mediated 1,5-HAT process. We anticipate that this discovery could inspire the development of the transformation of common aliphatic alcohols and Csp^3^-H functionalization.

## Methods

### General procedure for the functionalization of alkanols

AgNO_3_ (6.8 mg, 0.04 mmol), K_2_S_2_O_8_ (81 mg, 0.3 mmol), and reagent PhSO_2_C(CN)=NOBn (**A**) (120 mg, 0.4 mmol) were added to a 20 mL Schlenk tube under Ar. Aliphatic alcohols **1** (0.2 mmol) was added via syringe, followed by addition of acetone (1.0 mL), H_2_O (1.0 mL). The formed mixture was stirred at 50 °C under Ar for 24 h. After cooling to room temperature, the mixture was diluted with water (10 mL) and extracted with EA (3 × 10 mL). The combined extracts were washed with a saturated solution of NaCl (15 mL), dried over MgSO_4_, and evaporated in vacuo. The residue was purified by chromatography on silica gel (PE/EA = 5:1) to afford product **2**.

### Data availability

All data that support the findings of this study are available in the online version of this paper in the accompanying Supplementary Information (including experimental procedures, compound characterization data).

## Electronic supplementary material

Supplementary Information
