# Supplementary material for: Silver-catalyzed remote Csp3-H functionalization of aliphatic alcohols
Source: Nat Commun. 2018 Jul 6;9:2625. doi: 10.1038/s41467-018-05014-w (PMC6035238; doi:10.1038/s41467-018-05014-w)
Supplement: Supplementary file 1 — Supplementary Information [file 41467_2018_5014_MOESM1_ESM.pdf]

Supplementary Information for

Silver-Catalyzed Remote Csp^3-H Functionalization of Aliphatic Alcohols

Zhu et, al.

Supplementary Methods

General Information :

All manipulations were conducted with a standard Schlenk technique under argon atmosphere (1 atm). ^{1}H-NMR spectra were recorded with a Bruker AVIII-400 spectrometer. Chemical shifts (in ppm) were referenced to $CDCl_3$ (δ = 7.26 ppm) or TMS (δ = 0.00 ppm) as an internal standard. ^{13}C-NMR spectra were obtained by the same NMR spectrometer and were calibrated with $CDCl_3$ (δ = 77.00 ppm). Mass spectra were recorded by PE SCLEX QSTAR spectrometer. Unless otherwise noted, materials obtained from commercial suppliers were used without further purification. $AgNO_3$ was purchased from J&K.

Synthesis and Characterizations of Substrates

Alkanols **1a-d**, **1f**, **1l**, **1r-v** were purchased from commercial sources. **1e, 1g-k, 1m-q, 1w-x, S1**were prepared according to literature methods.

Typical procedure A:

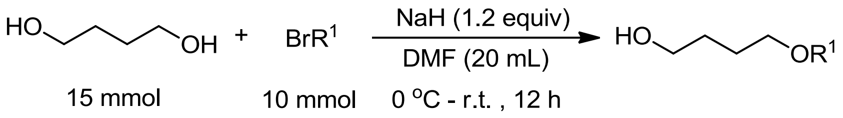

Sodium hydride (15 mmol, 1.5 equiv) was added at room temperature to a solution of alcohol (15 mmol, 1.5 equiv) in DMF (20 mL). The medium was stirred at room temperature during 1 h and alkyl bromide (10 mmol, 1.0 equiv) was added slowly. After 12 h at room temperature, water was added to quench the reaction and the organic layer was extracted with ethyl acetate, dried with Na_2SO_4 and concentrated under reduced pressure. The monoalkylated derivative was isolated as a colorless oil after purification by column chromatography with PE/EA (3:1).

Typical procedure B:

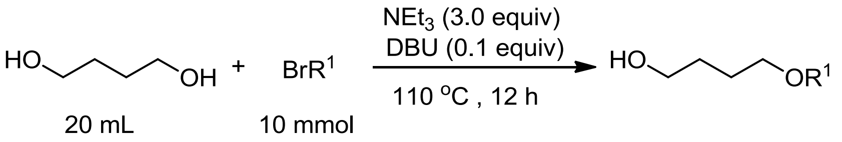

A mixture of 20 ml of butane-1,4-diol, alkyl bromide (10 mmol, 1.0 equiv), triethylamine (4.5 ml, 3.2 equiv) and 1,8-diazabicyclo[5.4.0]undec-7-ene (0.16 g, 0.1 equiv) was stirred at 110 ° for 12 h. The reaction mixture was poured into water (50 ml). Organic layer was extracted with chloroform (3 × 50 ml). The organic layer was washed with water (3×100 ml) and dried with Na_2SO_4 and concentrated under reduced pressure. The monoalkylated derivative was isolated as a colorless oil after purification by column chromatography with PE/EA (3:1).

Typical procedure C:

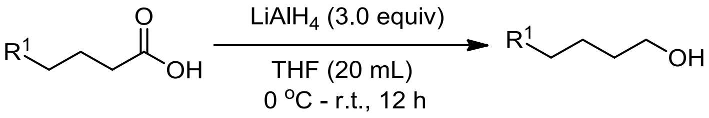

A solution of the carboxylic acid (10 mmol, 1.0 equiv) in 20 mL dry THF was added $LiAlH_4$ (1.2 g, 30 mmol, 3.0 equiv) potionwise at 0 °C. The reaction mixture was stirred overnight at room temperature. H_2O (1.2 mL) 15% NaOH (aq.) (1.2 mL,) and H_2O (3.6 mL) were added and stirred for another 30 min. The mixture was diluted with water (20 mL) and extracted with EA (3 × 20 mL). The combined extracts were washed with a saturated solution of NaCl (15 mL), dried over $MgSO_4$, and evaporated in vacuo and purified by column chromatography with PE/EA (5:1).

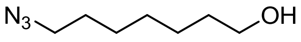

1e

7-azidoheptan-1-ol (1e)[1]

To a mixture 7-bromoheptan-1-ol (1.94 g, 10 mmol) in DMF (20 mL), NaN_3 (1.3 g, 20 mmol) was added potion-wise. The mixture was stirred at room temperature for 12 h. The reaction mixture was poured into water (30 ml). The organic layer was extracted with EA (3 × 30 ml). The organic layer was washed with water (3×100 ml) and dried with Na_2SO_4 and concentrated under reduced pressure to afford 1.46 g (93%) pure **1e** a colorless oil.

^{1}H NMR: (400 MHz, $CDCl_3$): δ 3.65 (t, *J* = 6.0 Hz, 2H), 3.40 (q, *J* = 6.8 Hz, 2H), 3.26 (t, *J* = 6.8 Hz, 1H), 1.87 (q, *J* = 6.8 Hz, 2H), 1.50-1.30 (m, 6H), 1.73-1.52 (m, 2H); **^{13}C NMR: (100 MHz, $CDCl_3$):** δ 62.9, 51.5, 32.6, 28.9, 28.8, 26.7, 25.6.

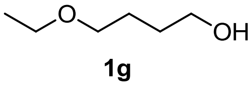

1g

4-ethoxybutan-1-ol (1g)[2]

Following Typical Procedure A, the reaction of butane-1,4-diol (1.5 g, 15 mmol), Bromoethane (1.1 g, 10 mmol) and NaH (60% oil) (0.6 g, 15 mmol) was reacted to afford 0.73 g (62%) **1g** as a colorless oil.

Colorless oil; **^{1}H NMR: (400 MHz, $CDCl_3$):** δ 3.65-3.64 (m, 2H), 3.52-3.45 (m, 4H), 2.62 (brs, 1H), 1.72-1.64 (m, 4H), 1.20 (t, *J* = 7.2 Hz, 3H); **^{13}C NMR: (100 MHz, $CDCl_3$):** δ 70.6, 66.3, 62.8, 30.5, 27.1, 15.0.

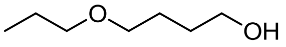

1h

4-propoxybutan-1-ol (1h)[3]

Following Typical Procedure A, the reaction of butane-1,4-diol (1.5 g, 15 mmol), 1-Bromopropane (1.2 g, 10 mmol) and NaH (60% oil) (0.6 g, 15 mmol) was reacted to afford 0.92 g (70%) **1h** as a colorless oil.

^{1}H NMR: (400 MHz, $CDCl_3$): δ 3.63-3.62 (m, 2H), 3.44 (t, *J* = 5.6 Hz, 2H), 3.38 (t, *J* = 6.4 Hz, 2H), 2.79 (brs, 1H), 1.70-1.54 (m, 6H), 0.90 (t, *J* = 7.2 Hz, 3H); **^{13}C NMR: (100 MHz, $CDCl_3$):** δ 72.7, 70.8, 62.7, 30.4, 26.9, 22.8, 10.5.

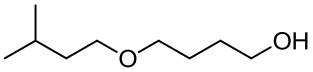

1i

4-(isopentyloxy)butan-1-ol (1i)[4]

Following Typical Procedure A, the reaction of butane-1,4-diol (1.5 g, 15 mmol), 1-Bromo-3-methylbutane (1.51 g, 10 mmol) and NaH (60% oil) (0.6 g, 15 mmol) was re acted to afford 1.10 g (69%) **1i** as a colorless oil.

^{1}H NMR: (400 MHz, $CDCl_3$): δ 3.64 (t, *J* = 5.6 Hz, 2H), 3.47-3.44 (m, 4H), 2.29 (brs, 1H), 1.70-1.65 (m, 5H), 1.46 (q, *J* = 6.8 Hz, 2H), 0.90 (d, *J* = 6.4 Hz, 6H); **^{13}C NMR: (100 MHz, $CDCl_3$):** δ 70.1, 69.5, 62.8, 38.4, 30.5, 27.1, 25.1, 22.6.

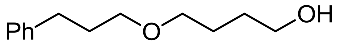

1j

4-(3-phenylpropoxy)butan-1-ol (1j)[5]

Following Typical Procedure A, the reaction of butane-1,4-diol (1.5 g, 15 mmol), 1-Bromo-3-phenylpropane (1.99 g, 10 mmol) and NaH (60% oil) (0.6 g, 15 mmol) wa s reacted to afford 1.54 g (74%) **1j** as a colorless oil.

^{1}H NMR: (400 MHz, $CDCl_3$): δ 7.29-7.26 (m, 2H), 7.19-7.16 (m, 3H), 3.67-3.64 (m, 2H), 3.47-3.43 (m, 4H), 2.69 (t, *J* = 7.2 Hz, 2H), 2.27 (brs, 1H), 1.94-1.87 (m, 2H),

1.71-1.66 (m, 4H); **^{13}C NMR: (100 MHz, $CDCl_3$):** δ 141.9, 128.5, 128.3, 125.8, 70.9, 70.1, 62.8, 32.3, 31.8, 30.4, 26.9.

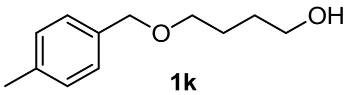

1k

4-((4-methylbenzyl)oxy)butan-1-ol (1k)

Following Typical Procedure A, the reaction of butane-1,4-diol (1.5 g, 15 mmol), 4-Methylbenzyl bromide (1.85 g, 10 mmol) and NaH (60% oil) (0.6 g, 15 mmol) was reacted to afford 1.26 g (65%) **1k** as a colorless oil.

^{1}H NMR: (400 MHz, $CDCl_3$): δ 7.23-7.14 (m, 4H), 4.47 (s, 2H), 3.62 (t, *J* = 6.4 Hz, 2H), 3.49 (t, *J* = 5.6 Hz, 2H), 2.34 (s, 3H), 2.31 (s, 1H), 1.72-1.64 (m, 4H); **^{13}C NMR: (100 MHz, $CDCl_3$):** δ 137.3, 135.1, 129.1, 127.8, 72.9, 70.1, 62.6, 30.1, 26.7, 21.1; **HRMS m/z (ESI)** calcd. for $C_{12}H_{18}NaO_2$ $(M + Na)^+$ 217.1199, found 217.1198.

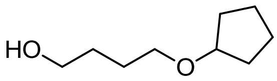

1m

4-(cyclopentyloxy)butan-1-ol (1m)

Following Typical Procedure B, the reaction of butane-1,4-diol (20 mL), Bromocycl opentane (1.49 g, 10 mmol), triethylamine (4.5 ml, 32 mmol) and DBU (0.16 g, 1 mm ol) was was reacted to afford 0.67 g (42%) **1m** as a colorless oil.

^{1}H NMR: (400 MHz, $CDCl_3$): δ 3.92-3.89 (m, 1H), 3.62 (t, *J* = 5.6 Hz, 2H), 3.42 (t, *J* = 5.6 Hz, 2H), 2.57 (brs, 1H), 1.73-1.63 (m, 10H), 1.52-1.49 (m, 2H); **^{13}C NMR: (100 MHz, $CDCl_3$):** δ 81.6, 68.8, 62.8, 32.2, 30.7, 27.4, 23.5.

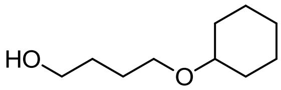

1n

4-(cyclohexyloxy)butan-1-ol (1n)[6]

Following Typical Procedure B, the reaction of butane-1,4-diol (20 mL), Bromocycl ohexane (1.63 g, 10 mmol), triethylamine (4.5 ml, 32 mmol) and DBU (0.16 g, 1 mm ol) was was reacted to afford 0.92 g (53%) **1n** as a colorless oil.

^{1}H NMR: (400 MHz, $CDCl_3$): δ 3.63 (t, *J* = 5.6 Hz, 2H), 3.48 (t, *J* = 5.6 Hz, 2H), 3.28-3.23 (m, 1H), 2.79 (brs, 1H), 1.91-1.88 (m, 2H), 1.76-1.65 (m, 6H), 1.53-1.49 (m, 1H), 1.30-1.21 (m, 5H); **^{13}C NMR: (100 MHz, $CDCl_3$):** δ 77.7, 67.8, 62.8, 32.0, 30.7, 27.5, 25.7, 24.0.

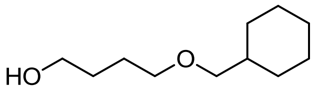

1o

4-(cyclohexylmethoxy)butan-1-ol (1o)

Following Typical Procedure A, the reaction of butane-1,4-diol (1.5 g, 15 mmol), Cyclohexylmethyl bromide (1.77 g, 10 mmol) and NaH (60% oil) (0.6 g, 15 mmol) was reacted to afford 1.44 g (77%) **1o** as a colorless oil.

^{1}H NMR: (400 MHz, $CDCl_3$): δ 3.64 (t, *J* = 5.6 Hz, 2H), 3.44 (t, *J* = 5.6 Hz, 2H), 3.23 (d, *J* = 6.4 Hz, 2H), 2.37 (brs, 1H), 1.76-1.52 (m, 10H), 1.26-1.09 (m, 3H), 0.97-0.87 (m, 2H); **^{13}C NMR: (100 MHz, $CDCl_3$):** δ 77.1, 71.1, 62.8, 38.0, 30.5, 30.1, 27.0, 26.6, 25.8; **HRMS m/z (ESI)** calcd. for $C_{11}H_{22}NaO_2$ $(M + Na)^+$ 209.1512, found 209.1513.

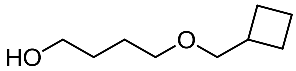

1p

4-(cyclobutylmethoxy)butan-1-ol (1p)

Following Typical Procedure A, the reaction of butane-1,4-diol (1.5 g, 15 mmol), (Bromomethyl)cyclobutane (1.49 g, 10 mmol) and NaH (60% oil) (0.6 g, 15 mmol) was reacted to afford 1.13 g (72%) **1p** as a colorless oil.

^{1}H NMR: (400 MHz, $CDCl_3$): δ 3.62 (t, *J* = 5.6 Hz, 2H), 3.45 (t, *J* = 5.6 Hz, 2H), 3.41 (d, *J* = 6.8 Hz, 2H), 2.64 (brs, 1H), 2.60-2.50 (m, 1H), 2.08-2.00 (m, 2H), 1.91-1.81 (m, 2H), 1.75-1.61 (m, 6H); **^{13}C NMR: (100 MHz, $CDCl_3$):** δ 75.7, 71.0, 62.7, 35.0, 30.4, 27.0, 25.1, 18.6; **HRMS m/z (ESI)** calcd. for $C_9H_{18}NaO_2$ $(M + Na)^+$ 181.1199, found 181.1200.

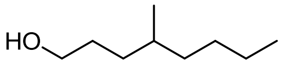

1q

4-methyloctan-1-ol (1q)[7]

Following Typical Procedure C, 4-Methyloctanoic acid (1.58 g, 10 mmol), $LiAlH_4$ (1.2 g, 30 mmol) in THF (20 mL) was reacted to afford 1.32 g (92%) **1q** as a colorless oil.

^{1}H NMR: (400 MHz, $CDCl_3$): δ 3.61 (t, *J* = 6.4 Hz, 2H), 1.65-1.47 (m, 3H), 1.42-1.23 (m, 7H), 1.15-1.09 (m, 2H), 0.89-9.85 (m, 6H); **^{13}C NMR: (100 MHz, $CDCl_3$):** δ 63.4, 36.6, 32.9, 32.6, 30.3, 29.2, 23.0, 19.6, 14.1.

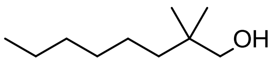

1w

2,2-dimethyloctan-1-ol (1w)[8]

Following Typical Procedure C, 2,2-dimethyloctanoic acid (1.72 g, 10 mmol), $LiAlH_4$ (1.2 g, 30 mmol) in THF (20 mL) was reacted to afford 1.38 g (87%) **1w** as a colorless oil. **^{1}H NMR: (400 MHz, $CDCl_3$):** δ 3.30 (s, 2H), 1.39-1.22 (m, 11H), 0.90-0.86 (m, 9H),; **^{13}C NMR: (100 MHz, $CDCl_3$):** δ 72.1, 38.7, 35.0, 31.9, 30.2, 23.81, 23.80, 22.7, 14.1.

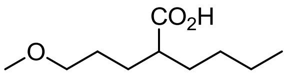

S1

2-(3-methoxypropyl)hexanoic acid (S1)

Diethyl methyl malonate (2.16 g, 10 mmol) and sodium hydride (60%, 0.5 g, 1.25 equiv) in glyme (30 mL) was added in a 100 mL flask and stirred for 30 min at 0 ^{o}C. 1-Bromo-3-methoxypropane (1.53 g, 1.0 equiv) in glyme (10 mL) was added dropwise and the mixture was stirred overnight at room temperature. The mixture was diluted with water (30 mL), and extracted with EtOAc (3 x 30 mL). The combined organic layer was evaporated under vacuo. The crude product was saponification with NaOH (1.6 g, 4.0 equiv) in MeOH (60 mL) at 60 ^{o}C for 6 h. After cooled to room temperature, the mixture was acidified to pH 2 (with aqueous 1 M HCl), and extracted with EtOAc (3 x 15 mL), the combined organic layers were washed with brine, dried (Na_2SO_4), filtered, and concentrated in vacuo. The residue was purified by chromatography to give 2-butyl-2-(3-methoxypropyl)malonic acid (1.95 g, 84%). On heating 1.95 g of this under nitrogen for 1 h in a bath at 160 ^{o}C, 2-(3-methoxypropyl)hexanoic acid (1.54 g, 97%) was obtained. **S1**, Colorless oil. **^{1}H NMR: (400 MHz, $CDCl_3$):** δ 3.40-3.35 (m, 2H), 3.32 (s, 3H), 2.37-2.33 (m, 1H), 1.68-1.45 (m, 6H), 1.32-1.24 (m, 4H), 0.88 (t, *J* = 7.2 Hz, 3H); **^{13}C NMR: (100 MHz, $CDCl_3$):** δ 182.3, 72.4, 58.4, 45.2, 31.9, 29.4, 28.7, 27.3, 22.5, 13.8; **HRMS m/z (ESI)** calcd. for $C_{10}H_{20}NaO_3$ $(M + Na)^+$ 211.1305, found 211.1307.

Synthesis and Characterizations of Sulphonyl Reagents

Reagents **A**-**E** were Prepared According to Literature Methods.

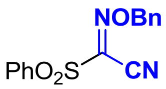

A

N-(benzyloxy)-1-(phenylsulfonyl)methanimidoyl cyanide (A)[9]

A solution of NaOEt in EtOH (prepared fresh from 790 mg, 34.33 mmol, 1.2 equiv. of Na and 18 ml of EtOH) was added to the suspension of (Phenylsulfonyl)acetonitrile (98 % Aldrich, 5.29 g, 28.61 mmol, 1 equiv.) in EtOH (7 ml) at RT. To the resulting clear solution isoamyl nitrite (96 % Sigma-Aldrich, 4.8 ml, 34.33 mmol, 1.2 equiv.) was added. The mixture was stirred at RT for 2 hours upon which a yellow solid precipitated. The mixture was cooled in an ice-bath, the solid was filtered and washed with cold EtOH and then with Et_2O. Drying under high vacuum afforded the sodium salt of N-hydroxy-1-(phenylsulfonyl)methanimidoyl cyanide as yellow powder (5.893 g, 89 %) that was used directly in the next step.

To the suspension of the sodium salt of N-hydroxy-1 (phenylsulfonyl)methanimidoyl cyanide (1 g, 4.307 mmol, 1 equiv.) in EtOH (10 ml) was added benzyl bromide (0.63 ml, 5.168 mmol, 1.2 equiv.) and the mixture was heated to reflux. A clear solution resulted within 2 minutes. After 1 hour the mixture was cooled to RT and concentrated under reduced pressure. EtOAc (80 ml) was added to the residue and was subsequently washed with sat. NH_4Cl (30 ml) and water (30 ml). The organic phase was dried over Na_2SO_4 and the solvent removed under reduced pressure. The crude product was purified by flash chromatography (hexane:EtOAc=3:2) to afford N-(benzyloxy)-1-(phenylsulfonyl)methanimidoyl cyanide (1g) (1.121 g, 87 %) as a white solid.

^{1}H NMR: (400 MHz, $CDCl_3$): δ 7.98 (d, *J* = 8.0 Hz, 2H), 7.76 (t, *J* = 7.2 Hz, 1H), 7.62 (t, *J* = 8.0 Hz, 2H), 7.39-7.35 (m, 3H), 7.33-7.30 (m, 2H), 5.44 (s, 2H); **^{13}C NMR: (100 MHz, $CDCl_3$):** δ 136.4, 135.4, 134.7, 133.6, 129.8, 129.3, 129.1, 128.7, 105.6, 81.5.

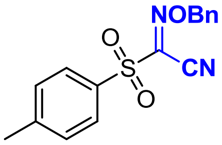

B

N-(benzyloxy)-1-tosylmethanimidoyl cyanide (B)[10]

A solution of NaOEt in EtOH (prepared fresh from 790 mg, 34.33 mmol, 1.2 equiv. of Na and 18 ml of EtOH) was added to the suspension of 2-tosylacetonitrile (5.85 g, 30 mmol, 1 equiv.) in EtOH (7 ml) at RT. To the resulting clear solution isoamyl nitrite (4.8 ml, 34.33 mmol, 1.2 equiv.) was added. The mixture was stirred at RT for 2 hours upon which a yellow solid precipitated. The mixture was cooled in an

ice-bath, the solid was filtered and washed with cold EtOH and then with Et_2O. Drying under high vacuum afforded the sodium salt of N-hydroxy-1-tosylmethanimidoyl cyanide as yellow powder (5.45 g, 74 %) that was used directly in the next step.

To the suspension of the sodium salt of N-hydroxy-1-tosylmethanimidoyl cyanide (5.45 g, 1 equiv.) in EtOH (30 ml) was added benzyl bromide (1.92 ml, 1.2 equiv.) and the mixture was heated to reflux. A clear solution resulted within 2 minutes. After 1 hour the mixture was cooled to RT and concentrated under reduced pressure. EtOAc (80 ml) was added to the residue and was subsequently washed with sat. NH_4Cl (30 ml) and water (30 ml). The organic phase was dried over Na_2SO_4 and the solvent removed under reduced pressure. The crude product was purified by flash chromatography (hexane:EtOAc=3:2) to afford N-(benzyloxy)-1-tosylmethanimidoyl cyanide (4.76 g, 68 %) as a white solid.

^{1}H NMR: (400 MHz, $CDCl_3$): δ 7.85 (d, J = 8.4 Hz, 2H), 7.41-7.31 (m, 7H), 5.43 (s, 2H), 2.49 (s, 3H); **^{13}C NMR: (100 MHz, $CDCl_3$):** δ 147.1, 135.0, 133.8, 133.5, 130.5, 129.35, 129.27, 129.1, 128.8, 105.8, 81.4, 21.8.

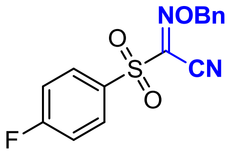

C

N-(benzyloxy)-1-((4-fluorophenyl)sulfonyl)methanimidoyl cyanide (C)

To 2-(4-Fluorophenylthio)acetonitrile (3.34g, 20 mmol, 1.0 equiv) in DCM (50 mL) was added 3-Chloroperoxybenzoic acid (5.16 g, 30 mmol, 1.5 equiv) at 0 °C. The reaction mixture was stirred for 1h at 0 °C and another 12 h at 40 °C. After cooling to room temperature, the mixture was diluted with DCM (50 mL) and $NaHCO_3$ (aq.) (50 mL). The organic layer was washed with Na_2SO_3 (aq.) (50 mL). Extracted with DCM (50 mL x 3). The organic phase was dried over Na_2SO_4 and the solvent removed under reduced pressure. The crude product was purified by flash chromatography (hexane:EtOAc=5:1) to afford 2-((4-fluorophenyl)sulfonyl)acetonitrile (2.43 g, 61%)

A solution of NaOEt in EtOH (prepared fresh from 260 mg, 12 mmol, 1.2 equiv. of Na and 6 ml of EtOH) was added to the suspension of 2-((4-fluorophenyl)sulfonyl)acetonitrile (2.00 g, 10 mmol, 1 equiv.) in EtOH (2.5 ml) at RT. To the resulting clear solution isoamyl nitrite (1.6 ml, 12 mmol, 1.2 equiv.) was added. The mixture was stirred at RT for 2 hours upon which a yellow solid precipitated. The mixture was cooled in an ice-bath, the solid was filtered and washed with cold EtOH and then with Et_2O. Drying under high vacuum afforded the sodium salt of 1-((4-

fluorophenyl)sulfonyl)-N-hydroxymethanimidoyl cyanide as yellow powder (1.12 g, 45 %) that was used directly in the next step.

To the suspension of the sodium salt of 1-((4-fluorophenyl)sulfonyl)-N-hydroxymethanimidoyl cyanide (1.12 g, 4.5 mmol, 1 equiv.) in EtOH (10 ml) was added benzyl bromide (0.66 ml, 5.4 mmol, 1.2 equiv.) and the mixture was heated to reflux. A clear solution resulted within 2 minutes. After 1 hour the mixture was cooled to RT and concentrated under reduced pressure. EtOAc (30 ml) was added to the residue and was subsequently washed with sat. NH_4Cl (20 ml) and water (20 ml). The organic phase was dried over Na_2SO_4 and the solvent removed under reduced pressure. The crude product was purified by flash chromatography (hexane:EtOAc=3:2) to afford N-(benzyloxy)-1-((4-fluorophenyl)sulfonyl)methanimidoyl cyanide (0.66 g, 46 %) as a white solid.

^{1}H NMR: (400 MHz, $CDCl_3$): δ 8.00-7.96 (m, 2H), 7.39-7.26 (m, 7H), 5.43 (s, 2H); **^{13}C NMR: (100 MHz, $CDCl_3$):** δ = 166.9 (d, *J* = 258.2 Hz), 134.7, 133.7, 132.4 (d, *J* = 3.3 Hz), 132.3 (d, *J* = 107.0 Hz), 129.4, 129.2, 128.8, 117.3 (d, *J* = 231.0 Hz), 105.6, 81.6; **^{19}F NMR: (376 MHz, $CDCl_3$):** δ -99.5; **HRMS m/z (ESI)** calcd. for $C_{15}H_{11}N_2O_3FNaS$ $(M + Na)^+$ 341.0367, found 341.0371.

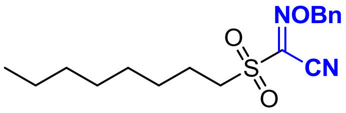

D

N-(benzyloxy)-1-(octylsulfonyl)methanimidoyl cyanide (D)

To 2-(octylthio)acetonitrile (3.7g, 20 mmol, 1.0 equiv) in DCM (50 mL) was added 3-Chloroperoxybenzoic acid (5.16 g, 30 mmol, 1.5 equiv) at 0 °C. The reaction mixture was stirred for 1h at 0 °C and another 12 h at 40 °C. After cooling to room temperature, the mixture was diluted with DCM (50 mL) and $NaHCO_3$ (aq.) (50 mL). The organic layer was washed with Na_2SO_3 (aq.) (50 mL). Extracted with DCM (50 mL x 3). The organic phase was dried over Na_2SO_4 and the solvent removed under reduced pressure. The crude product was purified by flash chromatography (hexane:EtOAc=5:1) to afford 2-(octylsulfonyl)acetonitrile (2.33 g, 54%)

A solution of NaOEt in EtOH (prepared fresh from 260 mg, 12 mmol, 1.2 equiv. of Na and 6 ml of EtOH) was added to the suspension of 2-(octylsulfonyl)acetonitrile (2.17 g, 10 mmol, 1 equiv.) in EtOH (2.5 ml) at RT. To the resulting clear solution isoamyl nitrite (1.6 ml, 12 mmol, 1.2 equiv.) was added. The mixture was stirred at RT for 2 hours upon which a yellow solid precipitated. The mixture was cooled in an ice-bath, the solid was filtered and washed with cold EtOH and then with Et_2O.

Drying under high vacuum afforded the sodium salt of N-hydroxy-1-(octylsulfonyl)methanimidoyl cyanide.

To the suspension of the sodium salt of N-hydroxy-1-(octylsulfonyl)methanimidoyl cyanide in EtOH (15 ml) was added benzyl bromide (1.55 ml, 12 mmol, 1.2 equiv.) and the mixture was heated to reflux. A clear solution resulted within 2 minutes. After 1 hour the mixture was cooled to RT and concentrated under reduced pressure. EtOAc (30 ml) was added to the residue and was subsequently washed with sat. NH_4Cl (20 ml) and water (20 ml). The organic phase was dried over Na_2SO_4 and the solvent removed under reduced pressure. The crude product was purified by flash chromatography (hexane:EtOAc=4:1) to afford N-(benzyloxy)-1-(octylsulfonyl)methanimidoyl cyanide (1.46 g, 59 % over two steps) as a colorless oil.

^{1}H NMR: (400 MHz, CDCl$_3$): δ 7.42-7.39 (m, 5H), 5.50 (s, 2H), 3.26-3.22 (m, 2H), 1.79-1.71 (m, 2H), 1.39-1.27 (m, 10H), 0.89 (t, J = 7.2 Hz, 3H); **^{13}C NMR: (100 MHz, CDCl$_3$):** δ = 133.8, 133.5, 129.5, 129.2, 128.9, 105.4, 81.6, 54.5, 31.6, 28.80, 28.79, 28.1, 22.5, 22.0, 14.0; **HRMS m/z (ESI)** calcd. for $C_{17}H_{24}N_2O_3NaS$ $(M + Na)^+$ 359.1400, found 359.1400.

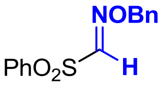

E

(Phenylsulfonyl)methanal O-benzyl oxime (1b)[11]

To a solution of O-benzylformaldoxime (1.35 g, 10 mmol) in DMF (25 mL) was added N-chlorosuccinimide (1.50 g, 11 mmol). The reaction mixture was heated for 3 h at 40oC, diluted with diethyl ether (100 ml), and washed with aqueous 10% HCI (2x50 mL) and brine (50 mL). The organic layer was dried over anhydrous MgSO4, filtered, and concentrated under reduced pressure. The crude product was purified by passing through a short column of silica gel (ethyl acetate : hexane = 1 : 50) to afford O-Benzylformohydroximoyl chloride (1.50 g, 88%):

To a solution of O-benzyl formhydroximoyl chloride (1.19 g, 7 mmol) in THF (20 mL) was added thiophenol sodium salt (1.39 g, 10.5 mmol). The reaction mixture was stirred for 3 h at room temperature, diluted with diethyl ether (60 mL) and washed with aqueous $NaHCO_3$ (2 x 30 mL) and brine (30 mL). The organic layer was dried over anhydrous $MgSO_4$, filtered, and concentrated under reduced pressure. The residue was chromatographed on a silica gel chromatography (ethyl acetate : hexane = 1 : 20) to give the product S-Phenyl N-(benzyloxy)-thioformimidate (1.53 g, 90%) colorless liquid.

To a solution of S-Phenyl N-(benzyloxy)-thioformimidate (486 mg, 2 mmol) in

CH_2Cl_2 (10mL) was added $NaHCO_3$ (336 mg, 4 mmol) and MCPBA (1.52 g, 4.4 mmol) at 0 °C**.** After being stirred for 1 h, the reaction mixture was heated for 1 h at 400C, diluted with CH_2Cl_2 (10 mL) and washed with aqueous $NaHCO_3$ (2x10 mL), aqueous Na_2SO_3 (10 mL) and brine (10 mL). The organic layer was dried over anhydrous $MgSO_4$**,** filtered, and concentrated under reduced pressure. The residue was chromatographed on a silica gel chromatography (ethyl acetate **:** hexane = 1 : 7) to give the product **E** as a white solid (484 mg, 88%)

^{1}H NMR: (400 MHz, $CDCl_3$): δ 7.92 (d, *J* = 7.6 Hz, 2H), 7.64 (t, *J* = 7.2 Hz, 1H), 7.47-7.44 (m, 3H), 7.28-7.26 (m, 3H), 7.04-7.02 (m, 2H), 5.12 (s, 2H); **^{13}C NMR: (100 MHz, $CDCl_3$):** δ 143.8, 139.1, 135.2, 134.3, 129.1, 128.8, 128.5, 128.4, 128.1, 78.7.

Supplementary Table 1: The solvent effect of δ-selective functionalization of primary alkanols

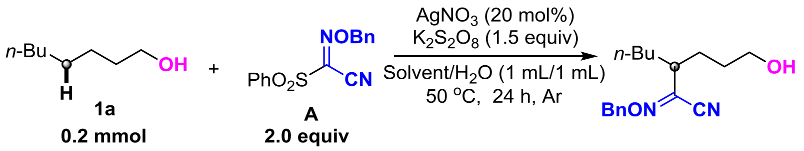

Entry	solvent	Results(%)[a]
1	CH_3CN	33
2	DMF	0
3	DMSO	<5
4	Dioxane	complex
5	PhCl	0
6	PhMe	0
7	$PhCF_3$	0
8	DCE	0
9	$(CF_3)_2CH_2OH$	0
10	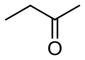	0
11	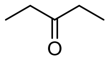	0

Reaction conditions: **1a** (0.2 mmol), $AgNO_3$ (0.04 mmol), $K_2S_2O_8$ (0.3 mmol), **A** (0.4 mmol), solvent/H_2O (1 mL/1 mL), stirred at 50 °C under Ar (1 atm) for 24 h. [a] isolated yields.

Supplementary Table 2: The test of several radical acceptors besides the oxime ether reagents

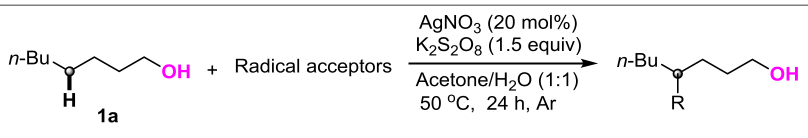

Entry	Radical acceptors	Predicted products	Results
1	PhSSPh	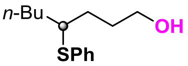	0%
2	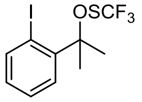	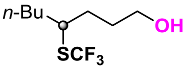	0%
3	*t*BuOCl	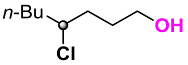	0%
4	TBN	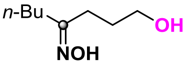	0%
5[a]	Selectflour	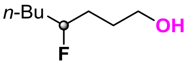	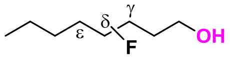 mixture product of ε,δ,γ C-H fluorinated alcohols
6	$TosN_3$	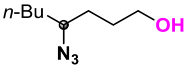	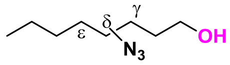 mixture product of ε,δ,γ C-H azidation alcohols

Reaction conditions: 1a (0.2 mmol), $AgNO_3$ (0.04 mmol), $K_2S_2O_8$ (0.3 mmol), Radical acceptors (0.4 mmol), Acetone/H_2O (1 mL/1 mL), stirred at 50 °C under Ar (1 atm) for 24 h. [a] without $K_2S_2O_8$

Synthesis of δ-functionalized aliphatic alcohols 2:

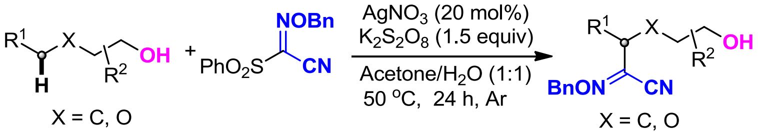

1 **A** **2**

$AgNO_3$ (6.8 mg, 0.04 mmol), $K_2S_2O_8$ (81 mg, 0.3 mmol) and reagent $PhSO_2C(CN)$=NOBn (**A**) (120 mg, 0.4 mmol) were added to a 20 mL Schlenk tube under Ar. Aliphatic alcohols **1** (0.2 mmol) was added via syringe, followed by addition of Acetone (1.0 mL), H_2O (1.0 mL). The formed mixture was stirred at 50 °C under Ar for 24 h. After cooling to room temperature, the mixture was diluted with water (10 mL) and extracted with EA (3 × 10 mL). The combined extracts were washed with a saturated solution of NaCl (15 mL), dried over $MgSO_4$, and evaporated

in vacuo. The residue was purified by chromatography on silica gel (PE/EA = 5:1) to afford product **2.**

Analytical Data for Products

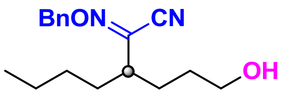

2a

N-(benzyloxy)-2-(3-hydroxypropyl)hexanimidoyl cyanide (2a) The reaction of octan-1-ol **1a** (26.0 mg, 0.2 mmol), $AgNO_3$ (6.8 mg, 0.04 mmol), $K_2S_2O_8$ (81 mg, 0.3 mmol) and reagent $PhSO_2C(CN)$=NOBn (**A**) (120 mg, 0.4 mmol) in Acetone (1.0 mL) and H_2O (1.0 mL) under Ar at 50 ºC for 24 h afforded 31.7 mg (55%) of product **2a** with 9.5:1 of two isomers, 5.7 mg (22%) of **1a** was recovered. **2a**: colorless oil; **^{1}H NMR: (400 MHz, CDCl3)** δ 7.39-7.30 (m, 5H), 5.25 (s, $2H_{major}$), 5.23 (s, $2H_{minor}$), 3.58 (t, *J* = 6.4 Hz, 2H), 2.53-2.45 (m, 1H), 1.68-1.43 (m, 6H), 1.36 (brs, 1H), 1.31-1.16 (m, 4H), 0.86 (t, *J* = 7.2 Hz, 3H); **^{13}C NMR: (100 MHz, CDCl3)** δ 136.9, 136.3, 128.6, 128.5, 128.3, 109.3, 77.6, 62.2, 42.0, 32.2, 29.9, 29.0, 28.6, 22.3, 13.8; **HRMS m/z (ESI)** calcd. for $C_{17}H_{25}N_2O_2$ $(M + H)^+$ 289.1916, found 289.1922.

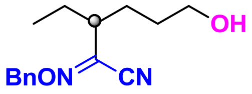

2b

N-(benzyloxy)-2-ethyl-5-hydroxypentanimidoyl cyanide (2b) The reaction of hexan-1-ol **1b** (20.4 mg, 0.2 mmol), $AgNO_3$ (6.8 mg, 0.04 mmol), $K_2S_2O_8$ (81 mg, 0.3 mmol) and $PhSO_2C(CN)$=NOBn (**A**) (120 mg, 0.4 mmol) in Acetone (1.0 mL) and H_2O (1.0 mL) under Ar at 50 ºC for 24 h afforded 31.3 mg (60%) of **2b** with 7:1 of two isomers, 1.0 mg (5%) of **1b** was recovered. **2b**: colorless oil; **^{1}H NMR: (400 MHz, CDCl3)** δ 7.35-7.31 (m, 5H), 5.25 (s, $2H_{major}$), 5.23 (s, $2H_{minor}$), 3.58 (t, *J* = 6.4 Hz, 2H), 2.43-2.40 (m, 1H), 1.70-1.54 (m, 4H), 1.52-1.41 (m, 3H), 0.86 (t, *J* = 7.6 Hz, 3H); **^{13}C NMR: (100 MHz, CDCl3)** δ 136.6, 136.3, 128.6, 128.5, 128.29, 128.26, 109.3, 77.6, 62.2, 43.7, 29.8, 28.3, 25.6, 11.3; **HRMS m/z (ESI)** calcd. for $C_{15}H_{20}O_2N_2Na$ $(M + Na)^+$ 283.1417, found 283.1419.

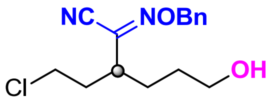

2c

N-(benzyloxy)-2-(2-chloroethyl)-5-hydroxypentanimidoyl cyanide (2c) The reaction of 6-chlorohexan-1-ol (95% pure.) **1c** (27.2 mg, 0.2 mmol), $AgNO_3$ (6.8 mg, 0.04 mmol), $K_2S_2O_8$ (81 mg, 0.3 mmol) and reagent $PhSO_2C(CN)$=NOBn (**A**) (120

mg, 0.4 mmol) in Acetone (1.0 mL) and H_2O (1.0 mL) under Ar at 50 ºC for 24 h afforded 28.5 mg (51%) of **2c** as a single isomer, 5.4 mg (21%) of **1c** was recovered. **2c**: colorless oil; **^{1}H NMR: (400 MHz, $CDCl_3$)** δ 7.40-7.32 (m, 5H), 5.26 (s, 2H), 3.60 (t, *J* = 6.4 Hz, 2H), 3.54-3.48 (m, 1H), 3.39-3.32 (m, 1H), 2.86-2.80 (m, 1H), 2.10-1.92 (m, 2H), 1.70-1.64 (m, 2H), 1.52-1.45 (m, 2H), 1.38 (brs, 1H); **^{13}C NMR: (100 MHz, $CDCl_3$)** δ 136.0, 135.1, 128.54, 128.46, 128.45, 109.1, 78.0, 61.9, 41.5, 39.3, 34.8, 29.5, 28.3; **HRMS m/z (ESI)** calcd. for $C_{15}H_{20}O_2N_2Cl$ $(M + H)^+$ 295.1208, found 295.1212.

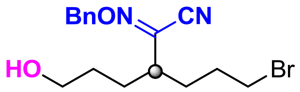

2d

N-(benzyloxy)-5-bromo-2-(3-hydroxypropyl)pentanimidoyl cyanide (2d) The reaction of 7-bromoheptan-1-ol **1d** (38.8 mg, 0.2 mmol), $AgNO_3$ (6.8 mg, 0.04 mmol), $K_2S_2O_8$ (81 mg, 0.3 mmol) and reagent $PhSO_2C(CN)$=NOBn (**A**) (120 mg, 0.4 mmol) in Actone (1.0 mL) and H_2O (1.0 mL) under Ar at 50 ºC for 24 h afforded 32.7 mg (46%) of **2d** as a single isomer, 11.6 mg (30%) of **1d** was recovered. **2d**: colorless oil; **^{1}H NMR: (400 MHz, $CDCl_3$)** δ 7.39-7.33 (m, 5H), 5.25 (s, 2H), 3.59 (t, *J* = 6.0 Hz, 2H), 3.32 (t, *J* = 6.0 Hz, 2H), 2.57-2.50 (m, 1H), 1.78-1.69 (m, 3H), 1.68-1.62 (m, 3H), 1.51-1.45 (m, 2H), 1.36 (brs, 1H); **^{13}C NMR: (100 MHz, $CDCl_3$)** δ 136.1, 136.07, 128.5, 128.39, 128.36, 109.1, 77.8, 62.0, 41.3, 32.6, 30.7, 29.69, 29.66, 28.6; **^{1}HRMS *m/z* (ESI)** calcd. for $C_{16}H_{22}O_2N_2Br$ $(M + H)^+$ 353.0859, found 353.0858.

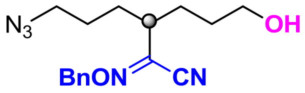

2e

5-azido-N-(benzyloxy)-2-(3-hydroxypropyl)pentanimidoyl cyanide (2e) The reaction of 7-azidoheptan-1-ol **1e** (31.4 mg, 0.2 mmol), $AgNO_3$ (6.8 mg, 0.04 mmol), $K_2S_2O_8$ (81 mg, 0.3 mmol) and reagent $PhSO_2C(CN)$=NOBn (**A**) (120 mg, 0.4 mmol) in Acetone (1.0 mL) and H_2O (1.0 mL) under Ar at 50 ºC for 24 h afforded 31.8 mg (50%) of **2e** as a single isomer, 5.3 mg (17%) of **1e** was recovered. **2e**: colorless oil; **^{1}H NMR: (400 MHz, $CDCl_3$)** δ 7.37-7.33 (m, 5H), 5.25 (s, 2H), 3.58 (t, *J* = 6.4 Hz, 2H), 3.23 (t, *J* = 6.4 Hz, 2H), 2.56-2.49 (m, 1H), 1.67-1.60 (m, 4H), 1.53-1.44 (m, 5H); **^{13}C NMR: (100 MHz, $CDCl_3$)** δ 136.1, 136.0, 128.5, 128.4, 128.3, 109.1, 77.7, 62.0, 50.8, 41.6, 29.7, 29.4, 28.6, 26.1; **^{1}HRMS *m/z* (ESI)** calcd. for $C_{16}H_{21}O_2N_5Na$ $(M + Na)^+$ 338.1588, found 338.1585.

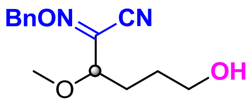

2f

N-(benzyloxy)-5-hydroxy-2-methoxypentanimidoyl cyanide (2f) The reaction of 4-methoxybutan-1-ol **1f** (20.6 mg, 0.2 mmol), $AgNO_3$ (6.8 mg, 0.04 mmol), $K_2S_2O_8$ (81 mg, 0.3 mmol) and reagent $PhSO_2C(CN)$=NOBn (**A**) (120 mg, 0.4 mmol) in Acetone (1.0 mL) and H_2O (1.0 mL) under Ar at 50 ºC for 24 h afforded 31.1 mg (60%) of **2f** with 7.5:1 of two isomers. **2f**: colorless oil; **^{1}H NMR: (400 MHz, $CDCl_3$)** δ 7.54-7.36 (m, 5H), 5.29 (s, $2H_{major}$), 5.27 (s, $2H_{minor}$), 4.52 (t, *J* = 6.4 Hz, $1H_{minor}$), 3.90 (t, *J* = 6.4 Hz, $1H_{minor}$), 3.63 (t, *J* = 6.0 Hz, 2H), 3.33 (s, $3H_{minor}$), 3.27 (s, $3H_{major}$), 1.94-1.84 (m, 1H), 1.80-1.72 (m, 1H), 1.70-1.57 (m, 3H); **^{13}C NMR: (100 MHz, $CDCl_3$)** δ 135.7, 134.0, 128.6, 128.5, 128.4, 108.5, 79.2, 78.3, 62.1, 57.0, 29.5, 28.1; **HRMS *m/z* (ESI)** calcd. for $C_{18}H_{26}O_2N_2Na$ $(M + Na)^+$ 285.1210, found 285.1207.

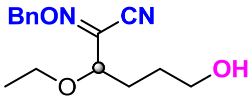

2g

N-(benzyloxy)-2-ethoxy-5-hydroxypentanimidoyl cyanide (2g) The reaction of 4-ethoxybutan-1-ol **1g** (23.6 mg, 0.2 mmol), $AgNO_3$ (6.8 mg, 0.04 mmol), $K_2S_2O_8$ (81 mg, 0.3 mmol) and reagent $PhSO_2C(CN)$=NOBn (**A**) (120 mg, 0.4 mmol) in Acetone (1.0 mL) and H_2O (1.0 mL) under Ar at 50 ºC for 24 h afforded 33.5 mg (61%) of **2g** with 10:1 of two isomers. **2g**: colorless oil; **^{1}H NMR: (400 MHz, $CDCl_3$)** δ 7.46-7.36 (m, 5H), 5.28 (s, $2H_{major}$), 5.25 (s, $2H_{minor}$), 4.61-4.58 (m, $1H_{minor}$), 4.02-3.98 (m, $1H_{major}$), 3.64 (t, *J* = 6.4 Hz, 2H), 3.52-3.45 (m, 1H), 3.42-3.33 (m, 1H), 1.95-1.86 (m, 1H), 1.80-1.70 (m, 1H), 1.68-1.55 (m, 3H), 1.23-1.16 (m, 3H); **^{13}C NMR: (100 MHz, $CDCl_3$)** δ 135.8, 134.6, 128.6, 128.5, 128.4, 108.7, 78.2, 77.4, 65.0, 62.2, 29.8, 28.3, 14.9; **HRMS *m/z* (ESI)** calcd. for $C_{15}H_{20}O_3N_2Na$ $(M + Na)^+$ 299.1366, found 299.1364.

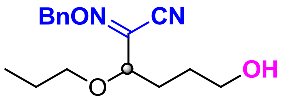

2h

N-(benzyloxy)-5-hydroxy-2-propoxypentanimidoyl cyanide (2h) The reaction of 2-4-propoxybutan-1-ol **1h** (26.4 mg, 0.2 mmol), $AgNO_3$ (6.8 mg, 0.04 mmol), $K_2S_2O_8$ (81 mg, 0.3 mmol) and reagent $PhSO_2C(CN)$=NOBn (**A**) (120 mg, 0.4 mmol) in Acetone (1.0 mL) and H_2O (1.0 mL) under Ar at 50 ºC for 24 h afforded 36.5 mg (63%) of **2h** with 11:1 of two isomers. **2h**: colorless; **^{1}H NMR: (400 MHz, $CDCl_3$)** δ 7.43-7.36 (m, 5H), 5.28 (s, $2H_{major}$), 5.26 (s, $2H_{minor}$), 4.60-4.57 (m, $1H_{minor}$), 4.00-3.97 (m, $1H_{major}$), 3.65 (brs, 2H), 3.38-3.33 (m, 1H), 3.29-3.25 (m, 1H), 1.93-1.89 (m, 1H), 1.78-1.71 (m, 1H), 1.69-1.52 (m, 5H), 0.89 (t, *J* = 7.6 Hz, 3H); **^{13}C NMR: (100**

MHz, $CDCl_3$) δ 135.8, 134.7, 128.6, 128.5, 128.4, 108.7, 78.2, 77.6, 71.3, 62.2, 29.8, 28.3, 22.7, 10.5; **HRMS *m/z* (ESI)** calcd. for $C_{15}H_{20}O_3N_2Na$ $(M + Na)^+$ 313.1523, found 313.1525.

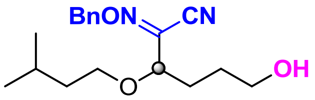

2i

N-(benzyloxy)-5-hydroxy-2-(isopentyloxy)pentanimidoyl cyanide (2i) The reaction of 4-(isopentyloxy)butan-1-ol **1i** (32.0 mg, 0.2 mmol), $AgNO_3$ (6.8 mg, 0.04 mmol), $K_2S_2O_8$ (81 mg, 0.3 mmol) and reagent $PhSO_2C(CN)$=NOBn (**A**) (120 mg, 0.4 mmol) in Acetone (1.0 mL) and H_2O (1.0 mL) under Ar at 50 ºC for 24 h afforded 38.2 mg (60%) of **2i** with 9:1 of two isomers, 2.7 mg (8%) of **1i** was recovered. **2i**: colorless oil; **^{1}H NMR: (400 MHz, $CDCl_3$)** δ 7.36-7.32 (m, 5H), 5.28 (s, $2H_{major}$), 5.26 (d, *J* = 2.0 Hz, $2H_{minor}$), 4.59-4.56 (m, $1H_{minor}$), 3.99-3.95 (m, $1H_{major}$), 3.64 (t, *J* = 6.4 Hz, 2H), 3.45-3.39 (m, 1H), 3.33-3.28 (m, 1H), 1.93-1.86 (m, 1H), 1.79-1.74 (m, 1H), 1.72-1.56 (m, 4H), 1.50-1.41 (m, 2H), 0.89-0.85 (m, 6H); **^{13}C NMR: (100 MHz, $CDCl_3$)** δ 135.8, 134.6, 128.7, 128.6, 128.5, 128.4, 108.7, 78.2, 77.7, 68.0, 62.2, 38.2, 29.8, 28.3, 24.8, 22.6, 22.5, 22.4; **HRMS *m/z* (ESI)** calcd. for $C_{18}H_{26}O_3N_2Na$ $(M + Na)^+$ 341.1836, found 341.1833.

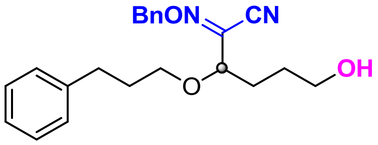

2j

N-(benzyloxy)-5-hydroxy-2-(3-phenylpropoxy)pentanimidoyl cyanide (2j) The reaction of 4-(3-phenylpropoxy)butan-1-ol **1j** (41.6 mg, 0.2 mmol), $AgNO_3$ (6.8 mg, 0.04 mmol), $K_2S_2O_8$ (81 mg, 0.3 mmol) and reagent $PhSO_2C(CN)$=NOBn (**A**) (120 mg, 0.4 mmol) in Acetone (1.0 mL) and H_2O (1.0 mL) under Ar at 50 ºC for 24 h afforded 38.9 mg (53%) of **2j** with >20:1 of two isomers**,** 5.8 mg (14%) of **1j** was recovered. **2j**: colorless oil; **^{1}H NMR: (400 MHz, $CDCl_3$)** δ 7.38-7.33 (m, 5H), 7.30-7.28 (m, 2H), 7.20-7.16 (m, 3H), 5.26 (s, 2H), 3.99-3.96 (m, 1H), 3.65 (t, *J* = 6.4 Hz, 2H), 3.44-3.39 (m, 1H), 3.33-3.27 (m, 1H), 2.70-2.62 (m, 2H), 1.96-1.84 (m, 3H), 1.81-1.71 (m, 1H), 1.70-1.57 (m, 2H), 1.51 (brs, 1H); **^{13}C NMR: (100 MHz, $CDCl_3$)** δ 141.5, 135.8, 134.4, 128.6, 128.5, 128.44, 128.35, 125.9, 108.7, 78.2, 77.7, 68.6, 62.2, 32.1, 31.0, 29.7, 28.2; **HRMS *m/z* (ESI)** calcd. for $C_{22}H_{26}O_3N_2Na$ $(M + Na)^+$ 389.1836, found 389.1833.

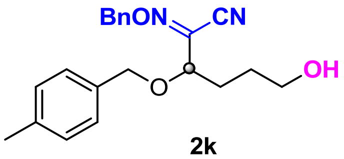

2k

N-(benzyloxy)-5-hydroxy-2-((4-methylbenzyl)oxy)pentanimidoyl cyanide (2k) The reaction of 4-((4-methylbenzyl)oxy)butan-1-ol **1k** (38.8 mg, 0.2 mmol), $AgNO_3$ (6.8 mg, 0.04 mmol), $K_2S_2O_8$ (81 mg, 0.3 mmol) and reagent $PhSO_2C(CN)$=NOBn (**A**) (120 mg, 0.4 mmol) in Acetone (1.0 mL) and H_2O (1.0 mL) under Ar at 50 ºC for 24 h afforded 45% **2k** and 20% **1k** was recovered (NMR yields with 0.2 mmol 1,1,2,2-Tetrachloroethane as an internal standard)**.** Further purification afford **2k** with 13:1 of two isomers. **2k**: colorless oil; **^{1}H NMR: (400 MHz, $CDCl_3$)** δ 7.42-7.34 (m, 5H), 7.18-7.09 (m, 4H), 5.33-5.23 (m, 2H), 4.69-4.66 (m, $1H_{minor}$), 4.57 (d, *J* = 11.2 Hz, $1H_{minor}$), 4.53 (d, *J* = 11.6 Hz, $1H_{major}$), 4.27 (d, *J* = 11.6 Hz, $1H_{minor}$), 4.22 (d, *J* = 11.6 Hz, $1H_{major}$), 4.08-4.05 (m, $1H_{major}$), 3.59 (t, *J* = 6.0 Hz, 2H), 2.34 (s, 3H), 1.98-1.89 (m, 1H), 1.80-1.72 (m, 1H), 1.68-1.61 (m, 1H), 1.58-1.50 (m, 1H), 1.40 (brs, 1H); **^{13}C NMR: (100 MHz, $CDCl_3$)** δ 137.9, 135.9, 134.3, 133.6, 129.1, 128.62, 128.57, 128.49, 128.36, 108.7, 78.2, 70.9, 62.1, 29.6, 28.1, 21.2; **HRMS *m/z* (ESI)** calcd. for $C_{21}H_{24}O_3N_2Na$ $(M + Na)^+$ 375.1679, found 375.1675.

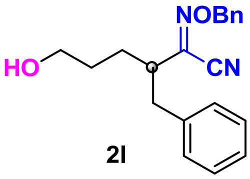

2l

2-benzyl-N-(benzyloxy)-5-hydroxypentanimidoyl cyanide (2l) The reaction of 5-phenylpentan-1-ol **1l** (32.8 mg, 0.2 mmol), $AgNO_3$ (6.8 mg, 0.04 mmol), $K_2S_2O_8$ (81 mg, 0.3 mmol) and reagent $PhSO_2C(CN)$=NOBn (**A**) (120 mg, 0.4 mmol) in Acetone (1.0 mL) and H_2O (1.0 mL) under Ar at 50 ºC for 24 h afforded 31.0 mg (48%) of **2l** with 3:1 of two isomers, 6.6 mg (20%) of **1l** was recovered. **2l**: colorless oil; **^{1}H NMR: (400 MHz, $CDCl_3$)** δ 7.36-7.31 (m, 4H), 7.27-7.10 (m, 6H), 5.27 (s, $2H_{minor}$), 5.16 (s, $2H_{major}$), 3.59-3.56 (m, 2H), 2.87-2.84 (m, 2H), 2.07-1.25 (m, 6H); **^{13}C NMR: (100 MHz, $CDCl_3$)** δ 137.9, 135.9, 134.3, 133.6, 129.1, 128.62, 128.57, 128.49, 128.36, 108.7, 78.2, 70.9, 62.1, 29.6, 28.1, 21.2. **HRMS *m/z* (ESI)** calcd. for $C_{20}H_{23}N_2O_2$ $(M + H)^+$ 323.1760, found 323.1754.

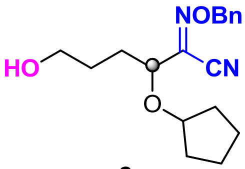

2m

N-(benzyloxy)-2-(cyclopentyloxy)-5-hydroxypentanimidoyl cyanide (2m) The reaction of 4-(cyclopentyloxy)butan-1-ol **1m** (31.7 mg, 0.2 mmol), $AgNO_3$ (6.8 mg, 0.04 mmol), $K_2S_2O_8$ (81 mg, 0.3 mmol) and reagent $PhSO_2C(CN)=NOBn$ (**A**) (120 mg, 0.4 mmol) in Acetone (1.0 mL) and H_2O (1.0 mL) under Ar at 50 °C for 24 h afforded 35.4 mg (56%) of **2m** with 16:1 of two isomers, 5.1 mg (16%) of **1m** was recovered. **2m**: colorless oil; **^{1}H NMR: (400 MHz, $CDCl_3$)** δ 7.39-7.32 (m, 5H), 5.28-5.25 (m, 2H), 4.64-4.61 (m, $1H_{minor}$), 4.05-4.02 (m, $1H_{major}$), 3.85-3.81 (m, 1H), 3.64 (t, *J* = 6.0 Hz, 2H), 1.90-1.80 (m, 1H), 1.76-1.54 (m, 10H), 1.51-1.47 (m, 2H); **^{13}C NMR: (100 MHz, $CDCl_3$)** δ 135.9, 135.2, 128.54, 128.49, 128.40, 108.8, 80.3, 70.1, 75.8, 62.2, 33.0, 31.7, 30.0, 28.4, 23.3, 23.2; **HRMS *m/z* (ESI)** calcd. for $C_{18}H_{24}O_3N_2Na$ $(M + Na)^+$ 339.1679, found 339.1677.

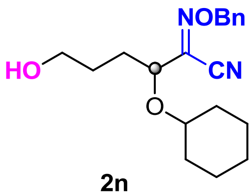

2n

N-(benzyloxy)-2-(cyclohexyloxy)-5-hydroxypentanimidoyl cyanide (2n)[6] The reaction of 4-(cyclohexyloxy)butan-1-ol **1n** (34.5 mg, 0.2 mmol), $AgNO_3$ (6.8 mg, 0.04 mmol), $K_2S_2O_8$ (81 mg, 0.3 mmol) and reagent $PhSO_2C(CN)=NOBn$ (**A**) (120 mg, 0.4 mmol) in Acetone (1.0 mL) and H_2O (1.0 mL) under Ar at 50 °C for 24 h afforded 42.1 mg (64%) of **2n** with 8:1 of two isomers, 4.8 mg (14%) of **1n** was recovered. **2n**: colorless oil; **^{1}H NMR: (400 MHz, $CDCl_3$)** δ 7.39-7.32 (m, 5H), 5.27-5.25 (m, 2H), 4.74-4.38 (m, $1H_{minor}$), 4.15-4.12 (m, $1H_{major}$), 3.64 (t, *J* = 6.0 Hz, 2H), 3.21-3.16 (m, 1H), 1.90-1.81 (m, 2H), 1.77-1.49 (m, 8H), 1.35-1.14 (m, 5H); **^{13}C NMR: (100 MHz, $CDCl_3$)** δ 136.0, 135.4, 128.54, 128.45, 128.3, 108.8, 78.0, 76.3, 74.7, 62.2, 42.2, 40.0, 36.1, 33.0, 31.2, 30.1, 28.3, 25.5, 24.0, 23.8. **HRMS *m/z* (ESI)** calcd. for $C_{19}H_{26}O_3N_2Na$ $(M + Na)^+$ 353.1836, found 353.1833.

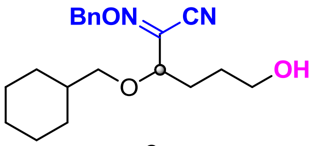

2o

N-(benzyloxy)-2-(cyclohexylmethoxy)-5-hydroxypentanimidoyl cyanide (2o) The reaction of 4-(cyclohexylmethoxy)butan-1-ol **1o** (37.3 mg, 0.2 mmol), $AgNO_3$ (6.8 mg, 0.04 mmol), $K_2S_2O_8$ (81 mg, 0.3 mmol) and reagent $PhSO_2C(CN)=NOBn$ (**A**) (120 mg, 0.4 mmol) in Acetone (1.0 mL) and H_2O (1.0 mL) under Ar at 50 °C for 24 h afforded 35.1 mg (51%) of **2o** with 18:1 of two isomers, 6.0 mg (16%) of **1o** was recovered. **2o**: colorless oil; **^{1}H NMR: (400 MHz, $CDCl_3$)** δ 7.39-7.33 (m, 5H), 5.28 (s, $2H_{major}$), 5.25 (d, *J* = 2.4 Hz, $2H_{minor}$), 4.57-4.54 (m, $1H_{minor}$), 3.96-3.93 (m,

1H$_{major}$), 3.64 (t, J = 5.6 Hz, 2H), 3.46-3.07 (m, 2H), 1.96-1.88 (m, 1H), 1.79-1.53 (m, 10H), 1.26-1.12 (m, 3H), 0.93-0.85 (m, 2H); **^{13}C NMR: (100 MHz, $CDCl_3$)** δ 135.8, 134.6, 128.6, 128.5, 128.4, 108.7, 78.1, 77.7, 75.3, 62.2, 37.8, 29.9, 29.8, 29.7, 28.2, 26.4, 25.72, 25.68. **HRMS *m/z* (ESI)** calcd. for $C_{20}H_{28}O_3N_2Na$ $(M + Na)^+$ 367.1992, found 367.1990.

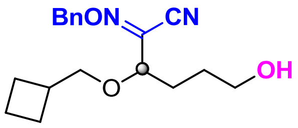

2p

N-(benzyloxy)-2-(cyclobutylmethoxy)-5-hydroxypentanimidoyl cyanide (2p) The reaction of 4-(cyclobutylmethoxy)butan-1-ol **1p** (31.7 mg, 0.2 mmol), $AgNO_3$ (6.8 mg, 0.04 mmol), $K_2S_2O_8$ (81 mg, 0.3 mmol) and reagent $PhSO_2C(CN)$=NOBn (**A**) (120 mg, 0.4 mmol) in Acetone (1.0 mL) and H_2O (1.0 mL) under Ar at 50 ºC for 24 h afforded 40.5 mg (64%) of **2p** with 12:1 of two isomers, 4.7 mg (15%) of **1p** was recovered. **2p**: colorless oil; **^{1}H NMR: (400 MHz, $CDCl_3$)** δ 7.40-7.33 (m, 5H), 5.29 (s, 2H$_{major}$), 5.26 (d, J = 2.0 Hz, 2H$_{minor}$), 4.59-4.56 (m, 1H$_{minor}$), 3.99-3.95 (m, 1H$_{major}$), 3.64 (t, J = 6.4 Hz, 2H), 3.38-3.32 (m, 1H), 3.29-3.25 (m, 1H), 2.56-2.48 (m, 1H), 2.06-1.98 (m, 2H), 1.96-1.82 (m, 3H), 1.79-1.65 (m, 4H), 1.64-1.61 (m, 2H); **^{13}C NMR: (100 MHz, $CDCl_3$)** δ 135.8, 134.6, 128.6, 128.5, 128.4, 108.7, 78.2, 77.6, 73.9, 62.1, 34.7, 29.7, 28.3, 24.9, 24.8, 18.5. **HRMS *m/z* (ESI)** calcd. for $C_{18}H_{24}O_3N_2Na$ $(M + Na)^+$ 339.1679, found 339.1678.

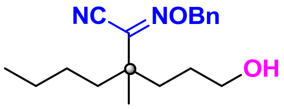

2q

N-(benzyloxy)-2-(3-hydroxypropyl)-2-methylhexanimidoyl cyanide (2q) The reaction of 4-methyloctan-1-ol **1q** (28.8 mg, 0.2 mmol), $AgNO_3$ (6.8 mg, 0.04 mmol), $K_2S_2O_8$ (81 mg, 0.3 mmol) and reagent $PhSO_2C(CN)$=NOBn (**A**) (120 mg, 0.4 mmol) in Acetone (1.0 mL) and H_2O (1.0 mL) under Ar at 50 ºC for 24 h afforded 18.8 mg (31%) of **2q** as a single isomer, 8.7 mg (30%) of **1q** was recovered. **2q**: colorless oil; **^{1}H NMR: (400 MHz, $CDCl_3$)** δ 7.35-7.33 (m, 5H), 5.25 (s, 2H), 3.57 (t, J = 6.4 Hz, 2H), 1.68-1.44 (m, 5H), 1.35-1.24 (m, 5H), 1.14-1.07 (m, 4H), 0.87 (t, J = 7.2 Hz, 3H); **^{13}C NMR: (100 MHz, $CDCl_3$)** δ 139.7, 136.4, 128.5, 128.4, 128.3, 109.5, 77.6, 62.9, 42.3, 38.8, 34.7, 27.1, 25.9, 23.0, 21.5, 13.9; **HRMS *m/z* (ESI)** calcd. for $C_{18}H_{26}O_2N_2Na$ $(M + Na)^+$ 325.1887, found 325.1885.

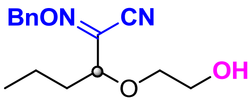

2r

N-(benzyloxy)-2-(2-hydroxyethoxy)pentanimidoyl cyanide (2r) The reaction of 2-butoxyethanol **1r** (23.6 mg, 0.2 mmol), $AgNO_3$ (6.8 mg, 0.04 mmol), $K_2S_2O_8$ (81 mg, 0.3 mmol) and reagent $PhSO_2C(CN)$=NOBn (**A**) (120 mg, 0.4 mmol) in Acetone (1.0 mL) and H_2O (1.0 mL) under Ar at 50 ºC for 24 h afforded 32.0 mg (58%) of **2r** with 12:1 of two isomers. **2r**: colorless oil; **^{1}H NMR: (400 MHz, $CDCl_3$)** δ 7.49-7.36 (m, 5H), 5.28 (s, $2H_{major}$), 5.26 (s, $2H_{minor}$), 4.64 (t, J = 6.4 Hz, $1H_{minor}$), 4.02 (t, J = 6.8 Hz, $1H_{major}$), 3.68 (brs, 2H), 3.52-3.42 (m, 2H), 2.04 (brs, 1H), 1.89-1.80 (m, 1H), 1.71-1.62 (m, 1H), 1.45-1.30 (m, 2H), 0.93 (t, J = 7.2 Hz, 3H); **^{13}C NMR: (100 MHz, $CDCl_3$)** δ 135.7, 134.2, 128.6, 128.54, 128.51, 128.4, 108.7, 78.2, 77.9, 70.5, 61.6, 34.8, 18.1, 13.5; **HRMS *m/z* (ESI)** calcd. for $C_{15}H_{20}O_3N_2Na$ $(M + Na)^+$ 299.1366, found 299.1364.

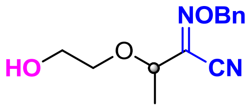

2s

N-(benzyloxy)-2-(2-hydroxyethoxy)butanimidoyl cyanide (2s) The reaction of 2-ethoxyethanol **1s** (18.0 mg, 0.2 mmol), $AgNO_3$ (6.8 mg, 0.04 mmol), $K_2S_2O_8$ (81 mg, 0.3 mmol) and reagent $PhSO_2C(CN)$=NOBn (**A**) (120 mg, 0.4 mmol) in Acetone (1.0 mL) and H_2O (1.0 mL) under Ar at 50 ºC for 24 h afforded 26.9 mg (54%) of **2s** with 9:1 of two isomers. **2s**: colorless oil; **^{1}H NMR: (400 MHz, $CDCl_3$)** δ 7.37-7.34 (m, 5H), 5.28 (s, $2H_{major}$), 5.26 (s, $2H_{minor}$), 4.77-4.72 (m, $1H_{minor}$), 4.24-4.19 (m, $1H_{major}$), 3.74-3.69 (m, 2H), 3.53-3.47 (m, 2H), 2.04 (brs, 1H), 1.45 (d, J = 6.8 Hz, $3H_{major}$), 1.36 (d, J = 6.4 Hz, $3H_{minor}$); **^{13}C NMR: (100 MHz, $CDCl_3$)** δ 135.6, 134.5, 128.7, 128.6, 128.4, 108.6, 78.3, 74.0, 70.3, 69.2, 61.6, 18.8; **HRMS *m/z* (ESI)** calcd. for $C_{13}H_{16}O_3N_2Na$ $(M + Na)^+$ 271.1053, found 271.1053.

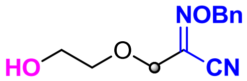

2t

N-(benzyloxy)-2-(2-hydroxyethoxy)acetimidoyl cyanide (2t) The reaction of 2-methoxyethanol **1t** (15.3 mg, 0.2 mmol), $AgNO_3$ (6.8 mg, 0.04 mmol), $K_2S_2O_8$ (81 mg, 0.3 mmol) and reagent $PhSO_2C(CN)$=NOBn (**A**) (120 mg, 0.4 mmol) in Acetone (0.6 mL) and H_2O (0.6 mL) under Ar at 50 ºC for 24 h afforded 15.5 mg (33%) of **2t** with 3.5:1 of two isomers. **2t**: colorless oil; **^{1}H NMR: (400 MHz, $CDCl_3$)** δ 7.37-7.35

(m, 5H), 5.30 (s, 2H*major*), 5.26 (s, 2H*minor*), 4.37 (s, 2H*minor*), 4.28 (s, 2H*major*), 3.79-3.74 (m, 2H), 3.64 (t, *J* = 4.0 Hz, 2H*minor*,) 3.59 (t, *J* = 4.0 Hz, 2H*major*), 1.90 (brs, 1H); **^{13}C NMR: (100 MHz, CDCl$_3$)** δ 135.5, 129.9, 128.9, 128.73, 128.69, 128.64, 128.61, 128.5, 109.6, 79.2, 78.5, 73.2, 72.2, 68.5, 64.5, 61.65, 61.61; **HRMS *m/z* (ESI)** calcd. for $C_{12}H_{14}O_3N_2Na$ (M + Na)$^+$ 257.0897, found 257.0895.

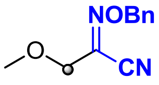

3t

N-(benzyloxy)-3-methoxypropanimidoyl cyanide (3t) The reaction of 2-methoxyethanol **1t** (15.3 mg, 0.2 mmol), $AgNO_3$ (6.8 mg, 0.04 mmol), $K_2S_2O_8$ (81 mg, 0.3 mmol) and reagent $PhSO_2C(CN)$=NOBn (**A**) (120 mg, 0.4 mmol) in Acetone (0.6 mL) and H_2O (0.6 mL) under Ar at 50 ºC for 24 h afforded 16.7 mg (41%) of **3t** with 3:1 of two isomers. **3t**: colorless oil; **^{1}H NMR: (400 MHz, CDCl$_3$)** δ 7.38-7.36 (m, 5H), 5.30 (s, 2H*major*), 5.26 (s, 2H*minor*), 4.25 (s, 2H*minor*), 4.17 (s, 2H*major*), 3.43 (s, 3H*minor*), 3.37 (s, 3H*major*); **^{13}C NMR: (100 MHz, CDCl$_3$)** δ 135.6, 135.4, 130.0, 129.3, 128.8, 128.7, 128.6, 128.5, 112.7, 109.6, 79.1, 78.4, 69.7, 65.8, 59.5, 58.5; **HRMS *m/z* (ESI)** calcd. for $C_{11}H_{13}O_2N_2$ (M + H)$^+$ 205.0972, found 205.0969.

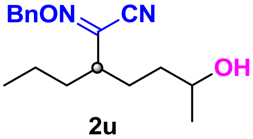

2u

N-(benzyloxy)-5-hydroxy-2-propylhexanimidoyl cyanide (2u) The reaction of octan-2-ol **1u** (26.0 mg, 0.2 mmol), $AgNO_3$ (6.8 mg, 0.04 mmol), $K_2S_2O_8$ (81 mg, 0.3 mmol) and reagent $PhSO_2C(CN)$=NOBn (**A**) (120 mg, 0.4 mmol) in Acetone (0.6 mL) and H_2O (0.6 mL) under Ar at 50 ºC for 24 h afforded 24.2 mg (42%) of **2u** with dr 2:1 of two isomers. **2u**: colorless oil; **^{1}H NMR: (400 MHz, CDCl$_3$)** δ 7.36-7.32 (m, 5H), 5.25 (s, 2H), 3.76-3.68 (m, 1H), 2.54-2.45 (m, 1H), 1.72-1.62 (m, 1H), 1.60-1.45 (m, 3H), 1.35-1.19 (m, 7H), 1.15-1.13 (m, 3H), 0.87 (t, *J* = 7.2 Hz, 3H); **^{13}C NMR: (100 MHz, CDCl$_3$)** δ 136.9, 136.3, 128.5, 128.3, 109.3, 77.6, 67.8, 67.3, 42.1, 41.8, 36.4, 36.1, 34.63, 34.56, 28.8, 28.3, 23.6, 23.5, 20.1, 20.0, 13.7; **HRMS *m/z* (ESI)** calcd. for $C_{17}H_{24}O_2N_2Na$ (M + Na)$^+$ 311.1730, found 311.1727.

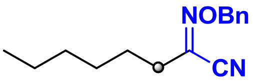

3u

N-(benzyloxy)heptanimidoyl cyanide (3u) The reaction of octan-2-ol **1u** (26.0 mg, 0.2 mmol), $AgNO_3$ (6.8 mg, 0.04 mmol), $K_2S_2O_8$ (81 mg, 0.3 mmol) and reagent

$PhSO_2C(CN)$=NOBn (**A**) (120 mg, 0.4 mmol) in Acetone (0.6 mL) and H_2O (0.6 mL) under Ar at 50 ºC for 24 h afforded 3.4 mg (7%) of **3u** with 2.1:1 of two isomers. **3u**: colorless oil; **^{1}H NMR: (400 MHz, $CDCl_3$)** δ 7.40-7.33 (m, 5H), 5.25 (s, $2H_{minor}$), 5.24 (s, $2H_{major}$), 2.48 (t, J = 8.0 Hz, $2H_{minor}$), 2.42 (t, J = 7.6 Hz, $2H_{major}$), 1.63-1.58 (m, 2H), 1.32-1.29 (m, 6H), 0.89 (t, J = 7.2 Hz, 3H); **^{13}C NMR: (100 MHz, $CDCl_3$)** δ 139.6, 136.2, 135.9, 128.6, 128.5, 128.33, 128.31, 114.6, 110.4, 78.2, 77.6, 31.9, 31.22, 31.21, 28.6, 28.2, 27.8, 26.1, 25.3, 22.39, 22.37, 13.9; **HRMS *m/z* (ESI)** calcd. for $C_{15}H_{19}ON_2$ (M - H) 243.1497, found 243.1500.

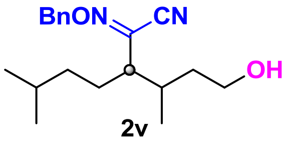

2v

N-(benzyloxy)-2-(4-hydroxybutan-2-yl)-5-methylhexanimidoyl cyanide (2v) The reaction of 3,7-dimethyloctan-1-ol **1v** (31.7 mg, 0.2 mmol), $AgNO_3$ (6.8 mg, 0.04 mmol), $K_2S_2O_8$ (81 mg, 0.3 mmol) and reagent $PhSO_2C(CN)$=NOBn (**A**) (120 mg, 0.4 mmol) in Acetone (1.0 mL) and H_2O (1.0 mL) under Ar at 50 ºC for 24 h afforded 28.5 mg (45%) of **2v** with dr 2:1 of two isomers, 6.9 mg (24%) of **1v** was recovered. **2v**: colorless oil; **^{1}H NMR: (400 MHz, $CDCl_3$)** δ 7.37-7.32 (m, 5H), 5.26-5.23 (m, 2H), 3.69-3.54 (m, 2H), 2.33-2.28 (m, 1H), 1.89-1.46 (m, 5H), 1.35-1.28 (m, 3H), 1.09-0.91 (m, 4H), 0.86-0.83 (m, 6H); **^{13}C NMR: (100 MHz, $CDCl_3$)** δ 136.45, 136.42, 136.38, 135.9, 129.0, 128.3, 128.2, 110.0, 109.9, 77.5, 60.52, 60.48, 48.0, 47.2, 36.88, 36.85, 36.5, 36.3, 32.6, 32.4, 27.8, 27.4, 26.5, 22.7, 22.6, 22.1, 22.0, 16.9, 16.5; **HRMS *m/z* (ESI)** calcd. for $C_{19}H_{28}O_2N_2Na$ $(M + Na)^+$ 339.2043, found 339.2042.

The Byproduct Analysis:

The results and the identified byproducts in some cases were summarized in **Supplementary Table 3.** For some substrates such as **1l**, **1q**, the alcohols were recovered. For some substrates such as **1t**, **1u**, the main identified byproducts were β-scission products.

Supplementary Table 3: The mass balance analysis of selected reactions

Selected Substrates	The mass balance analysis[a]
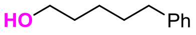 **1l**	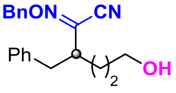 **2l**, 48% + recovered **1l** 20% + Undetectable fragments
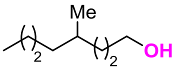 **1q**	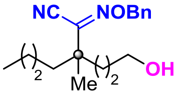 **2q**, 34% + recovered **1q** 33% + Undetectable fragments
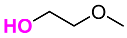 **1t**	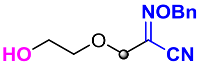 **2t**, 33% + 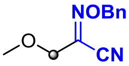 **3t**, 41% + Undetectable fragments
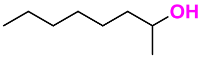 **1u**	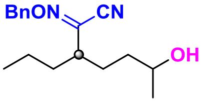 **2u**, 42% d.r. 2:1[b] + 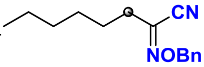 **3u**, 7% + Undetectable fragments

[a] Reaction conditions: substrate (0.2 mmol), $AgNO_3$ (0.04 mmol), $K_2S_2O_8$ (0.3 mmol), $PhSO_2(CN)NOBn$ (0.4 mmol), Acetone/H_2O (0.6 mL/0.6 mL), stirred at 50 °C under Ar (1 atm) for 24 h. [b] Diastereoselectivity was determined by ^{1}H NMR.

The Identification of Activation Site

As the alkanols have multiple carbon-hydrogen bonds with similar reactivity, the functionalized position is supposed to be determined. Thus, we designed an experiment to clarify the substitution position of alcohol substrates. With the developed radical decarboxylative strategy,[11] the 2-(3-methoxypropyl)hexanoic acid **S1** could transformed to the corresponding oxime ether **8** in 66% yield (**Supplementary Fig. 1a**). Meanwhile, the functionalized alcohol **2a** modified from **1a** in our conditions was reacted with iodomethane to afford **S-8** in 14% yield (**Supplementary Fig. 1b**). We were glad to see that the ^{1}H NMR of **8** and **S-8** are almost identical except for different E/Z isomer ratio of the oxime ether groups (**Supplementary Fig. 2**). This is a powerful evidence that the functionalized position is the δ carbon-hydrogen bond of the alkanols.

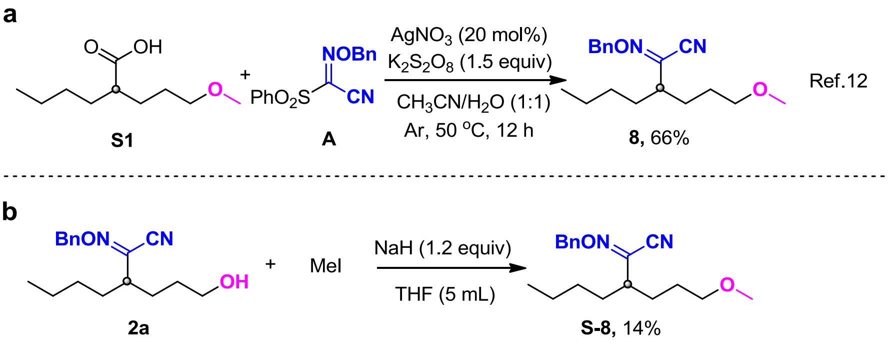

Supplementary Figure 1. Synthesis of **8** and **S-8**. **a** Synthesis of **8** via reported decarboxylative stragegy. **b** Synthsis of **S-8** from **2a**.

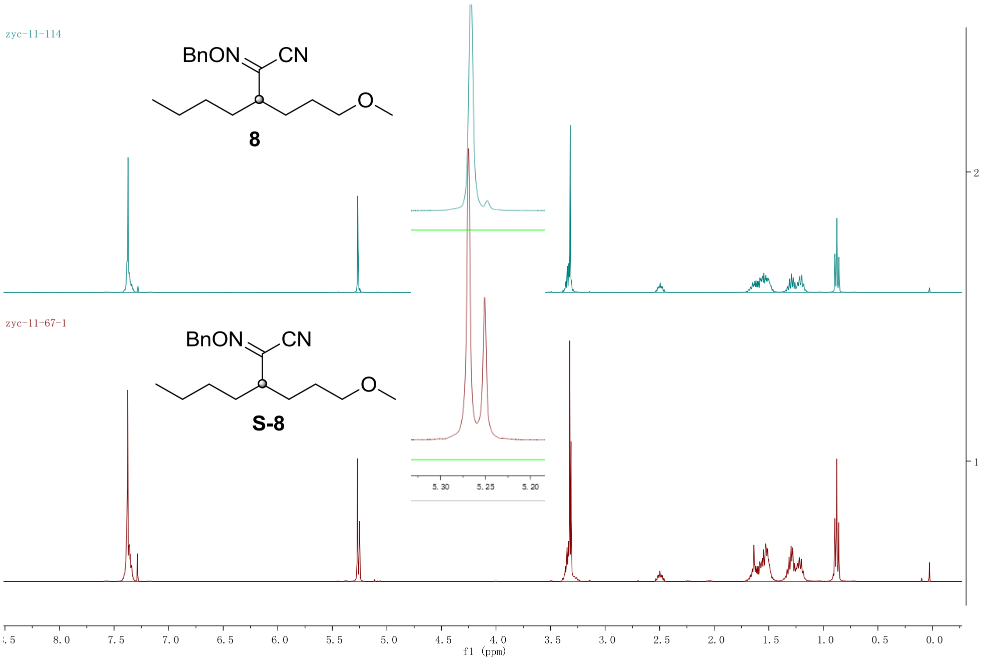

Supplementary Figure 2. ^{1}H NMR of **8** and **S-8**

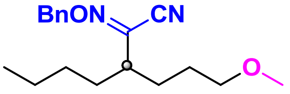

8

N-(benzyloxy)-2-(3-methoxypropyl)hexanimidoyl cyanide (8)[12]

The reaction of 2-(3-methoxypropyl)hexanoic acid **S1** (37.6 mg, 0.2 mmol), $AgNO_3$ (6.8 mg, 0.04 mmol), $K_2S_2O_8$ (81 mg, 0.3 mmol) and reagent $PhSO_2C(CN)$=NOBn (**A**) (120 mg, 0.4 mmol) in CH_3CN (1.0 mL) and H_2O (1.0 mL) under Ar at 50 ºC for 12 h afforded 39.9 mg (66%) of **8** with 13:1 of two isomers. **8**: colorless oil; **^{1}H NMR: (400 MHz, $CDCl_3$)** δ 7.38-7.32 (m, 5H), 5.24 (s, $2H_{major}$), 5.23 (m, $2H_{minor}$), 3.36-3.27 (m, 5H), 2.51-2.44 (m, 1H), 1.66-1.46 (m, 6H), 1.32-1.16 (m, 4H), 0.85 (t, J = 7.2 Hz, 3H); **^{13}C NMR: (100 MHz, $CDCl_3$)** δ 136.9, 136.2, 128.4, 128.24, 128.20, 109.3,

77.5, 71.9, 58.5, 42.0, 32.1, 28.9, 26.9, 22.3, 13.8; **HRMS *m/z* (ESI)** calcd. for $C_{18}H_{26}O_2N_2Na$ (M + Na)$^+$ 325.1887, found 325.1887.

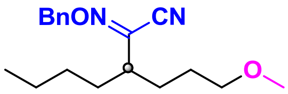

S-8

N-(benzyloxy)-2-(3-methoxypropyl)hexanimidoyl cyanide (S-8)

The reaction of N-(benzyloxy)-2-(3-hydroxypropyl)hexanimidoyl cyanide **2a** (184.4 mg, 0.64 mmol), NaH (60% in oil) (30.7 mg, 1.2 equiv), MeI (136 mg, 1.2 equiv) in THF (5.0 mL) at r.t. for 12 h afforded 26.5 mg (14%) of **S-8** with 2:1 of two isomers. **S-8**: colorless oil; **^{1}H NMR: (400 MHz, $CDCl_3$)** δ 7.37-7.31 (m, 5H), 5.24 (s, 2H$_{major}$), 5.23 (m, 2H$_{minor}$), 3.34-3.29 (m, 5H), 2.49-2.45 (m, 1H$_{major}$), 1.62-1.47 (m, 6H), 1.31-1.18 (m, 4H+1H$_{minor}$), 0.85 (t, *J* = 7.2 Hz, 3H); **^{13}C NMR: (100 MHz, $CDCl_3$)** δ 143.9, 136.9, 136.3, 135.9, 128.50, 128.49, 128.47, 128.3, 128.2, 113.2, 109.3, 78.2, 77.6, 71.9, 58.5, 42.0, 37.1, 32.1, 32.0, 29.2, 29.0, 27.1, 26.9, 22.4, 22.3, 13.8; **HRMS *m/z* (ESI)** calcd. for $C_{18}H_{26}O_2N_2Na$ (M + Na)$^+$ 325.1887, found 325.1884.

Mechanistic Studies

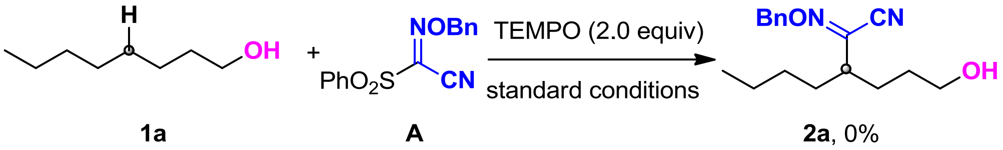

1) $AgNO_3$ (6.8 mg, 0.04 mmol), $K_2S_2O_8$ (81 mg, 0.3 mmol), TEMPO (62.5 mg, 0.4 mmol) and reagent $PhSO_2C(CN)$=NOBn (**A**) (120 mg, 0.4 mmol) were added to a 20 mL Schlenk tube under Ar. Octan-1-ol **1a** (26.0 mg, 0.2 mmol) was added via syringe, followed by addition of Acetone (1.0 mL), H_2O (1.0 mL). The formed mixture was stirred at 50 ºC under Ar for 24 h. After cooling to room temperature, the mixture detected by TLC. No **2a** was detected.

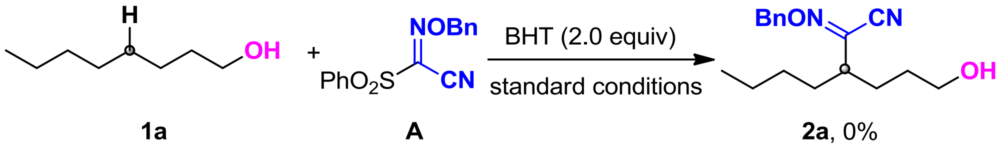

2) $AgNO_3$ (6.8 mg, 0.04 mmol), $K_2S_2O_8$ (81 mg, 0.3 mmol), BHT (88.1 mg, 0.4 mmol) and reagent $PhSO_2C(CN)$=NOBn (**A**) (120 mg, 0.4 mmol) were added to a 20 mL Schlenk tube under Ar. Octan-1-ol **1a** (26.0 mg, 0.2 mmol) was added

via syringe, followed by addition of Acetone (1.0 mL), H_2O (1.0 mL). The formed mixture was stirred at 50 ºC under Ar for 24 h. After cooling to room temperature, the mixture detected by TLC. No **2a** was detected.

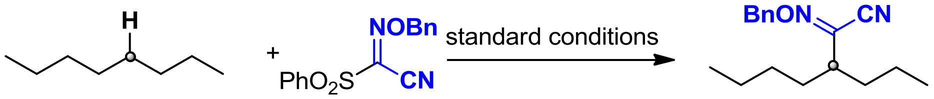

5 **A** **6**, 0%

3) $AgNO_3$ (6.8 mg, 0.04 mmol), $K_2S_2O_8$ (81 mg, 0.3 mmol), and reagent $PhSO_2C(CN){=}NOBn$ (**A**) (120 mg, 0.4 mmol) were added to a 20 mL Schlenk tube under Ar. 1-Octane **5** (22.8 mg, 0.2 mmol) was added via syringe, followed by addition of Acetone (1.0 mL), H_2O (1.0 mL). The formed mixture was stirred at 50 ºC under Ar for 24 h. After cooling to room temperature, the mixture was diluted with water (10 mL) and extracted with EA (3 × 10 mL). The combined extracts were washed with a saturated solution of NaCl (15 mL), dried over $MgSO_4$, and evaporated in vacuo. The residue was purified by chromatography on silica gel (PE/EA = 5:1) to afford 2.1 mg undefined product, no aim product was detected**.**

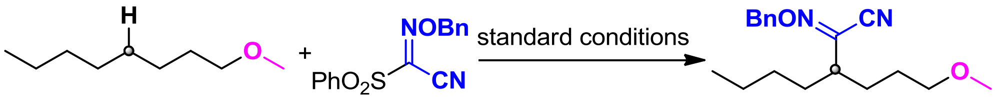

7 **A** **8**, 0%

4) $AgNO_3$ (6.8 mg, 0.04 mmol), $K_2S_2O_8$ (81 mg, 0.3 mmol), and reagent $PhSO_2C(CN){=}NOBn$ (**A**) (120 mg, 0.4 mmol) were added to a 20 mL Schlenk tube under Ar. 1-methoxyoctane **7** (28.8 mg, 0.2 mmol) was added via syringe, followed by addition of Acetone (1.0 mL), H_2O (1.0 mL). The formed mixture was stirred at 50 ºC under Ar for 24 h. After cooling to room temperature, the mixture was diluted with water (10 mL) and extracted with EA (3 × 10 mL). The combined extracts were washed with a saturated solution of NaCl (15 mL), dried over $MgSO_4$, and evaporated in vacuo. The residue was purified by chromatography on silica gel (PE/EA = 5:1) , no aim product was detected**.**

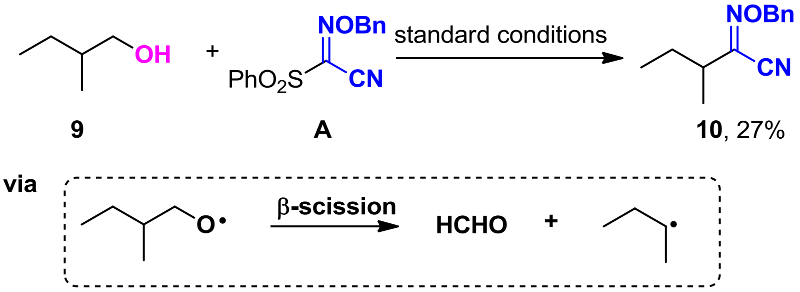

5) The reaction of 2-methylbutan-1-ol **9** (44.1 mg, 0.5 mmol), $AgNO_3$ (17.0 mg, 0.1 mmol), $K_2S_2O_8$ (202.5 mg, 0.75 mmol) and reagent $PhSO_2C(CN)$=NOBn (**A**) (300 mg, 1.0 mmol) in Acetone (2.5 mL) and H_2O (2.5 mL) under Ar at 50 °C for 24 h afforded 28.9 mg (27%) of **10** with 13:1 of two isomers.

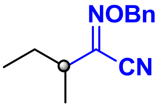

10

N-(benzyloxy)-2-methylbutanimidoyl cyanide (10)

10: colorless oil; **^{1}H NMR: (400 MHz, $CDCl_3$)** δ 7.40-7.32 (m, 5H), 5.25 (s, 2H), 3.24-3.18 (m, 1H_{minor}), 2.58-2.49 (m, 1H_{major}), 1.67-1.60 (m, 1H), 1.58-1.49 (m, 1H), 1.19 (d, *J* = 6.8 Hz, 3H_{major}), 1.13 (d, *J* = 6.8 Hz, 3H_{minor}), 0.91-0.86 (m, 3H); **^{13}C NMR: (100 MHz, $CDCl_3$)** δ 137.5, 136.2, 128.5, 128.29, 128.28, 109.5, 77.6, 38.4, 33.3, 27.0, 17.5, 11.3; **HRMS *m/z* (ESI)** calcd. for $C_{18}H_{26}O_2N_2Na$ $(M + Na)^+$ 239.1155, found 239.1152.

Radical clock experiment:

We synthesized the cyclopropane substrate **1x.** Under standard conditions, **1x** was consumed. However, we didn't obtain the ring-opening product. In contrast, a mixture product of complex products was detected. We infer this is because the following three reasons: 1) the addition of ring-opening primary alkyl radical to reagent **A** is difficult. 2) the generated alkene product **S2** was broken by $K_2S_2O_8$. 3) the produced product **S2** might undergo further radical addition or cyclization reaction with the internal alkene group.

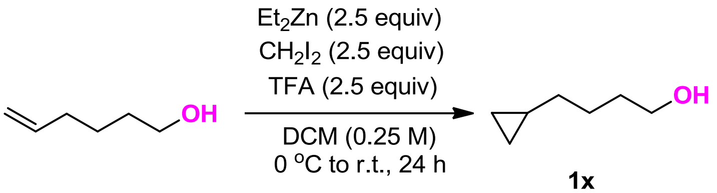

1x

To a flame-dried 100-mL flask was added DCM (40 mL, 0.25 M), hex-5-en-1-ol (1.2 mL, 10 mmol, 1.0 equiv), and diiodomethane (1.2 mL, 25 mmol, 2.5 equiv). The solution was cooled to 0 ºC and diethylzinc (25 mL of a 1.0 M solution in hexanes, 25 mmol, 2.5 equiv) was added dropwise over 10 min. Trifluoroacetic acid (1.9 mL, 25 mmol, 2.5 equiv) was added dropwise over 10 min. The reaction was stirred for 24 h under nitrogen while the ice bath was allowed to slowly warm to r.t. The reaction was opened to air and quenched with sat. aq. NH_4Cl. The solution was extracted with DCM (×3). The organic fractions were combined and washed with brine (×1), dried over $MgSO_4$, and concentrated. The crude residue was then added to a 50-mL flask with DCM (20 mL, 0.50 M), and m-CPBA (0.49 g of a 70% w/w suspension in H_2O, 2.0 mmol, 0.20 equiv) was added. The solution was stirred for 2 h open to air and then quenched with sat. aq. NH_4Cl. The mixture was extracted with DCM (×3), and the organic fractions were combined, washed with sat. aq. $NaHCO_3$ (×1) and brine (×1), dried over $MgSO_4$, and concentrated. The crude residue was purified by flash column chromatography (8/2 hexanes/EtOAc) to yield **1x** as a colorless, fruity-smelling oil (0.21 g, 1.8 mmol, 18%).

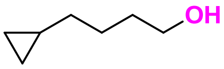

1x

4-cyclopropylbutan-1-ol (1x)[13]

1x: colorless oil; **^{1}H NMR: (400 MHz, $CDCl_3$)** δ 3.64 (t, J = 6.8 Hz, 2H), 1.63-1.56 (m, 2H), 1.49-1.42 (m, 3H), 1.25-1.19 (m, 2H), 0.67-0.64 (m, 1H), 0.42-0.37 (m, 2H), 0.02-(-0.02) (m, 2H); **^{13}C NMR: (100 MHz, $CDCl_3$)** δ 63.1, 34.5, 32.6, 25.8, 10.8, 4.3;.

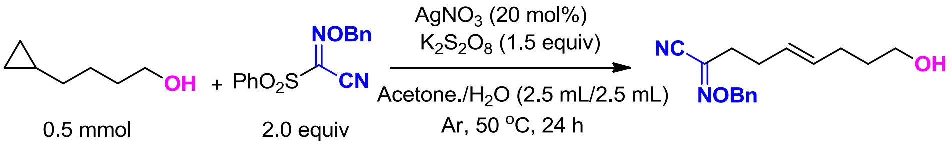

0.5 mmol **1x** 2.0 equiv **A** **S2**, undetected

The reaction of 2-methylbutan-1-ol **1x** (57.0 mg, 0.5 mmol), $AgNO_3$ (17.0 mg, 0.1 mmol), $K_2S_2O_8$ (202.5 mg, 0.75 mmol) and reagent $PhSO_2C(CN)$=NOBn (**A**) (300 mg, 1.0 mmol) in Acetone (2.5 mL) and H_2O (2.5 mL) under Ar at 50 ºC for 24 h

afforded a mixture product of complex products

The reactivity of reagent E:

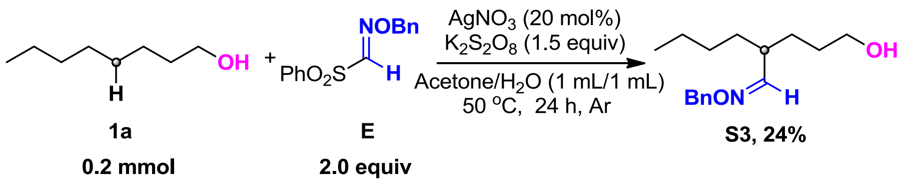

We tested the reactivity of reagent **E** [$PhSO_2CH_2$(H)NOBn] with **1a** as the substrate under standard conditions, and the corresponding product was isolated in 24% yield, which is lower compared to reagent **A** [$PhSO_2CH_2$(CN)NOBn]. In our previous report,[12] the reactivity of reagent **A** and **E** was tested and the reaction rate of reagent **A** was three times faster compared to reagent **E**.

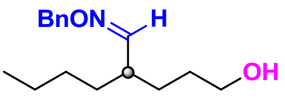

S3

2-(3-hydroxypropyl)hexanal O-benzyl oxime (S3)

The reaction of octan-1-ol **1a** (26.0 mg, 0.2 mmol), $AgNO_3$ (6.8 mg, 0.04 mmol), $K_2S_2O_8$ (81 mg, 0.3 mmol) and reagent $PhSO_2C$(H)=NOBn (**E**) (110 mg, 0.4 mmol) in Acetone (1.0 mL) and H_2O (1.0 mL) under Ar at 50 ºC for 24 h afforded 12.6 mg (24%) of product **S3** with E/Z=3:1 of two isomers. **S3**: colorless oil; **^{1}H NMR: (400 MHz, $CDCl_3$)** δ 7.36-7.29 (m, 5H), 7.22 (d, J = 8.4 Hz, $1H_E$), 6.43 (d, J = 8.4 Hz, 1Hz), 5.08 (s, 2Hz), 5.06 ($2H_E$), 3.61-3.58 (m, 2H), 3.11-3.06 (m, 1Hz), 2.26-2.20 (m, $1H_E$), 1.58-1.21 (m, 11H), 0.87 (t, J = 6.8 Hz, 3H); **^{13}C NMR: (100 MHz, $CDCl_3$)** δ 156.3, 155.3, 138.6, 137.8, 128.4, 128.3, 128.26, 128.0, 127.8, 75.5, 62.7, 39.6, 37.1, 35.3, 32.9, 30.2, 29.2, 29.0, 22.6, 14.0; **HRMS m/z (ESI)** calcd. for $C_{16}H_{26}NO_2$ $(M + H)^+$ 264.1964, found 264.1966.

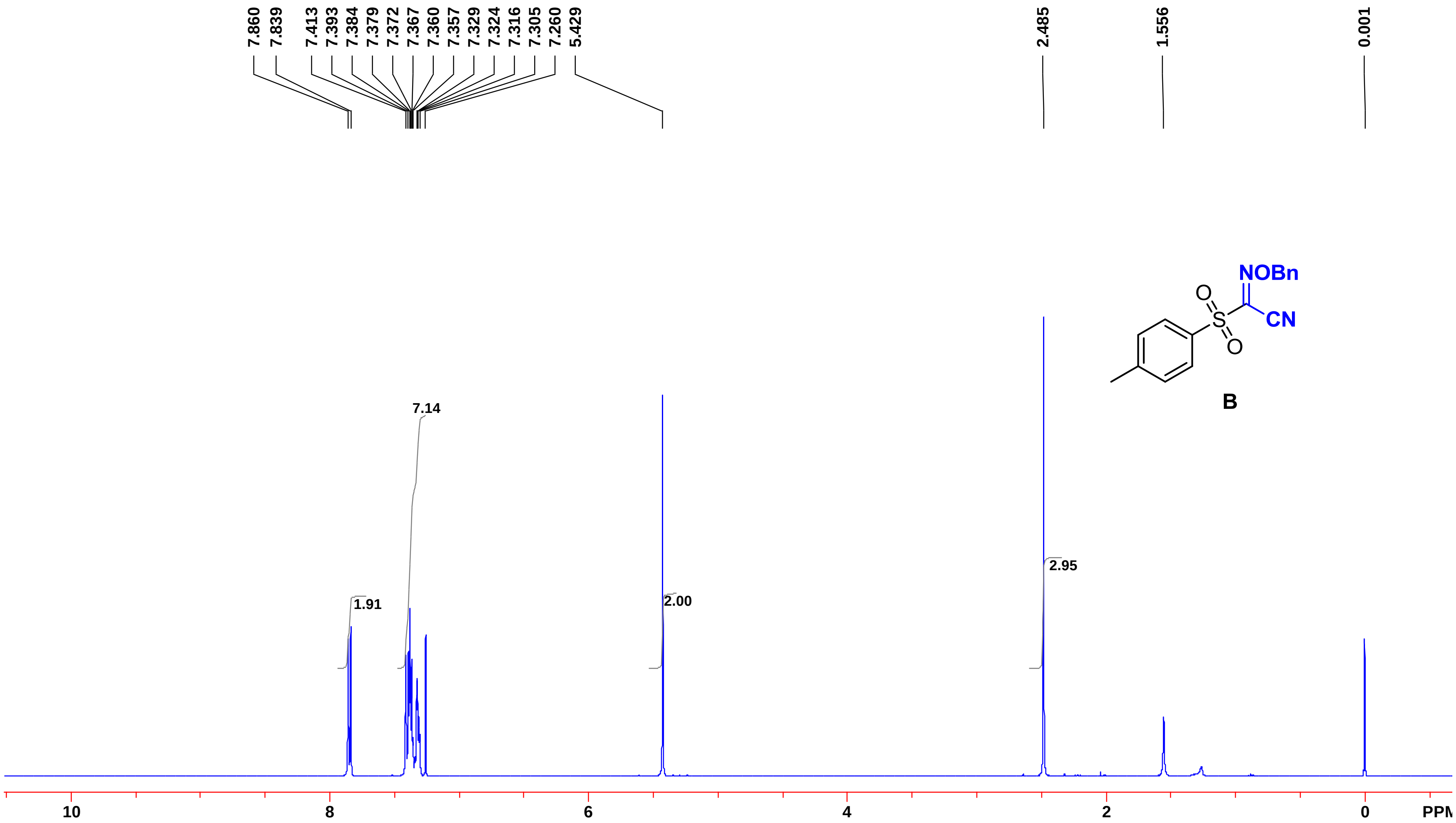

Supplementary Figure 3. ^{1}H NMR of **B**

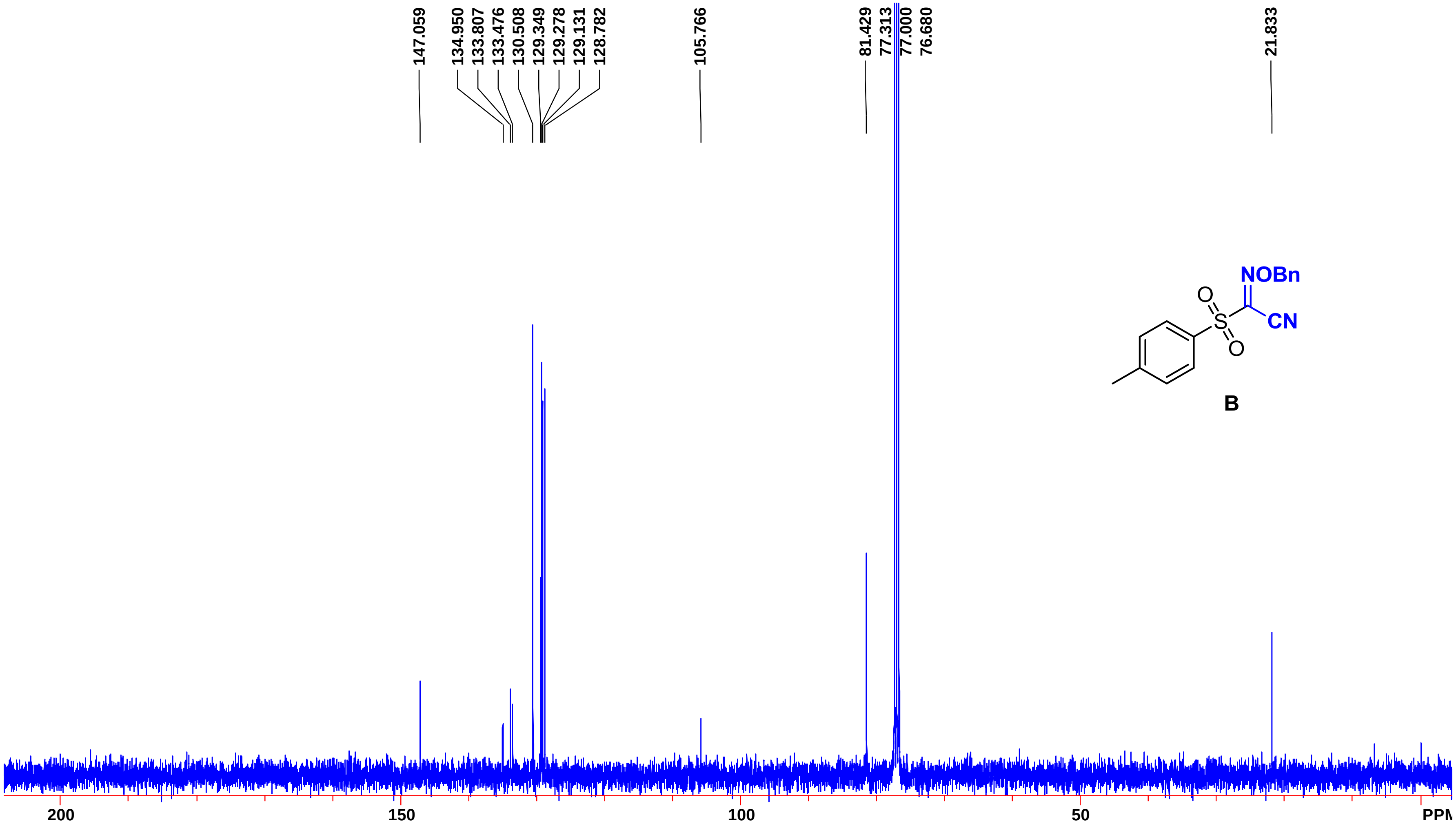

Supplementary Figure 4. ^{13}C NMR of **B**

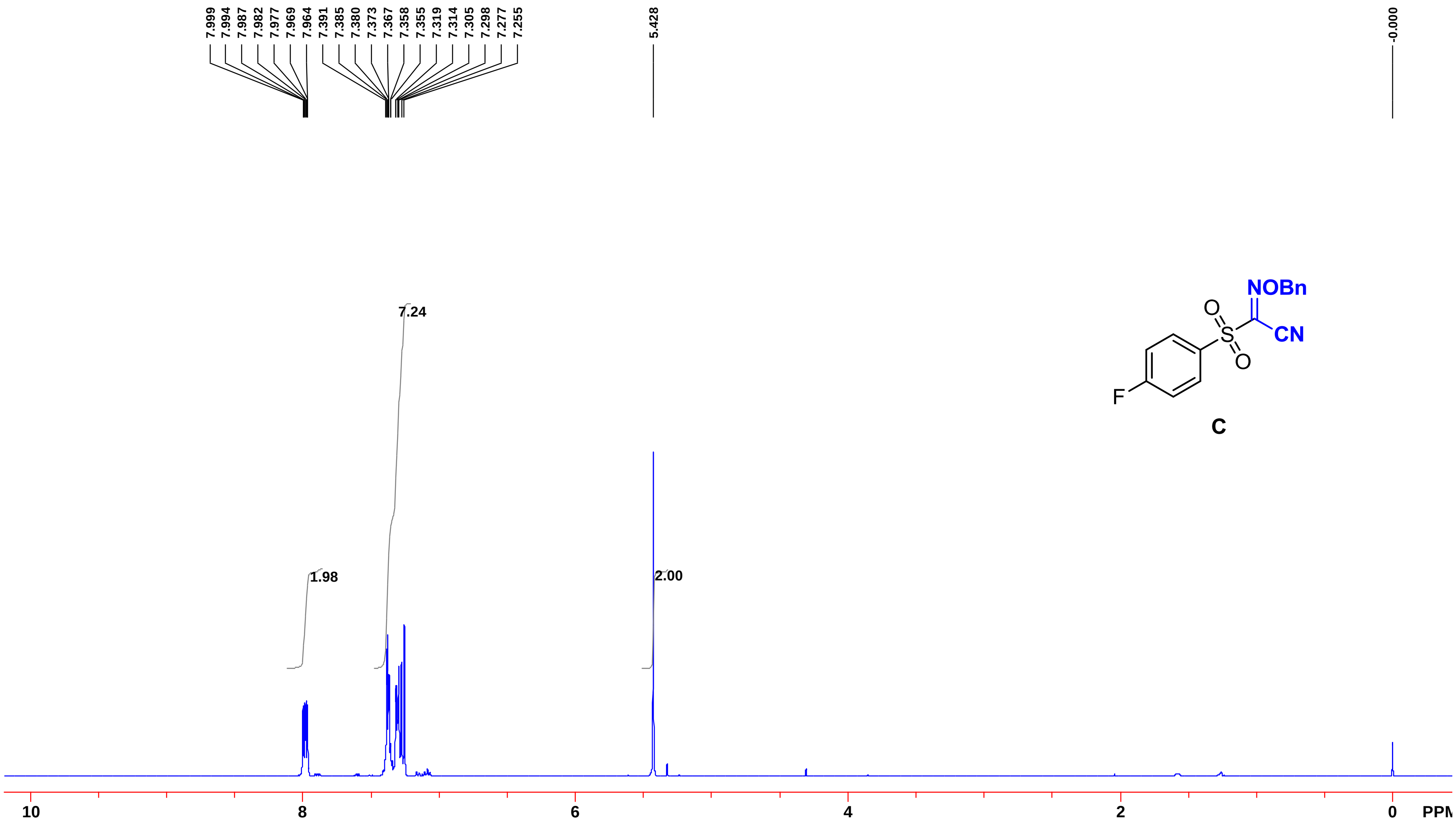

Supplementary Figure 5. ^{1}H NMR of **C**

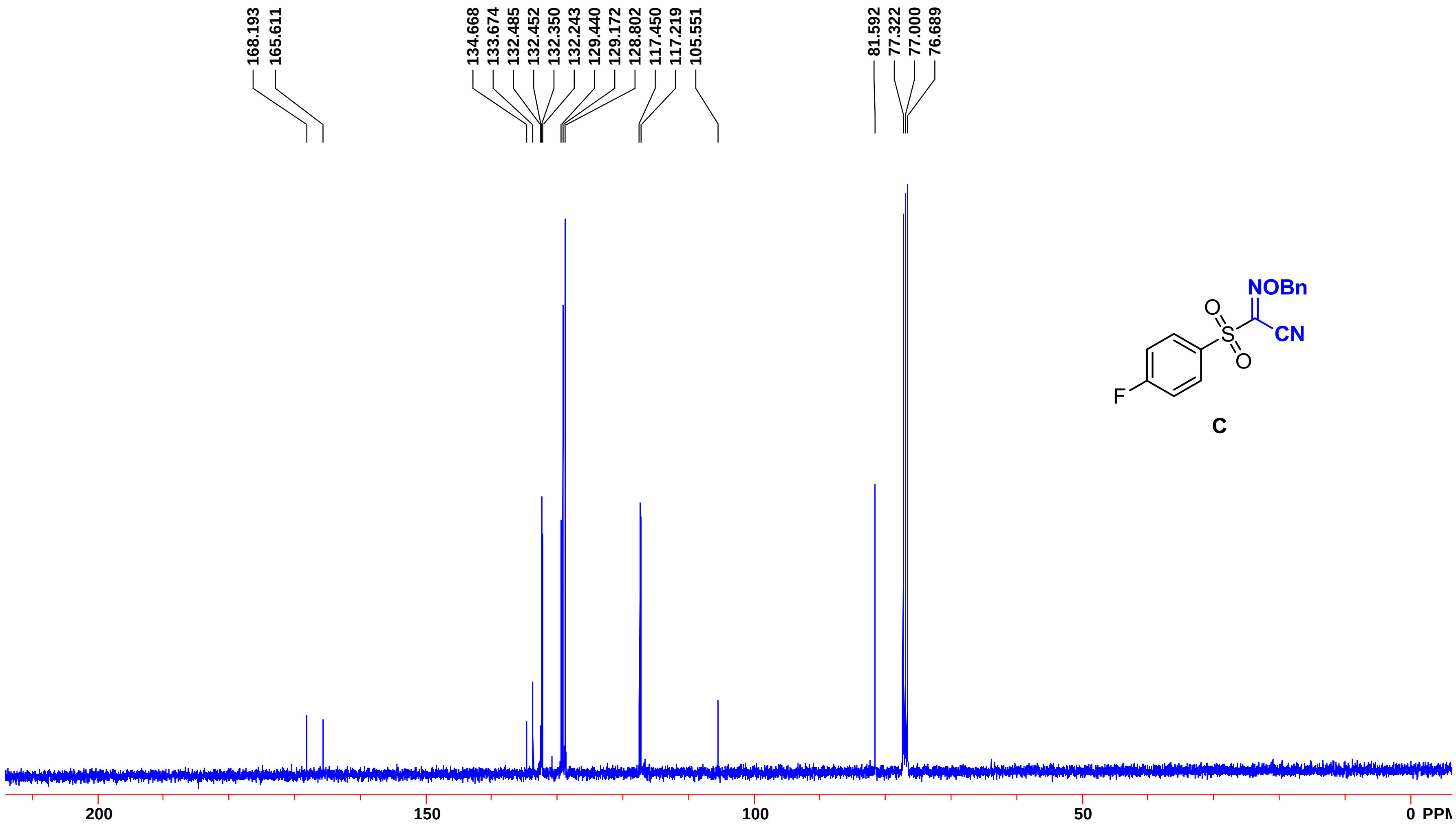

Supplementary Figure 6. ^{13}C NMR of **C**

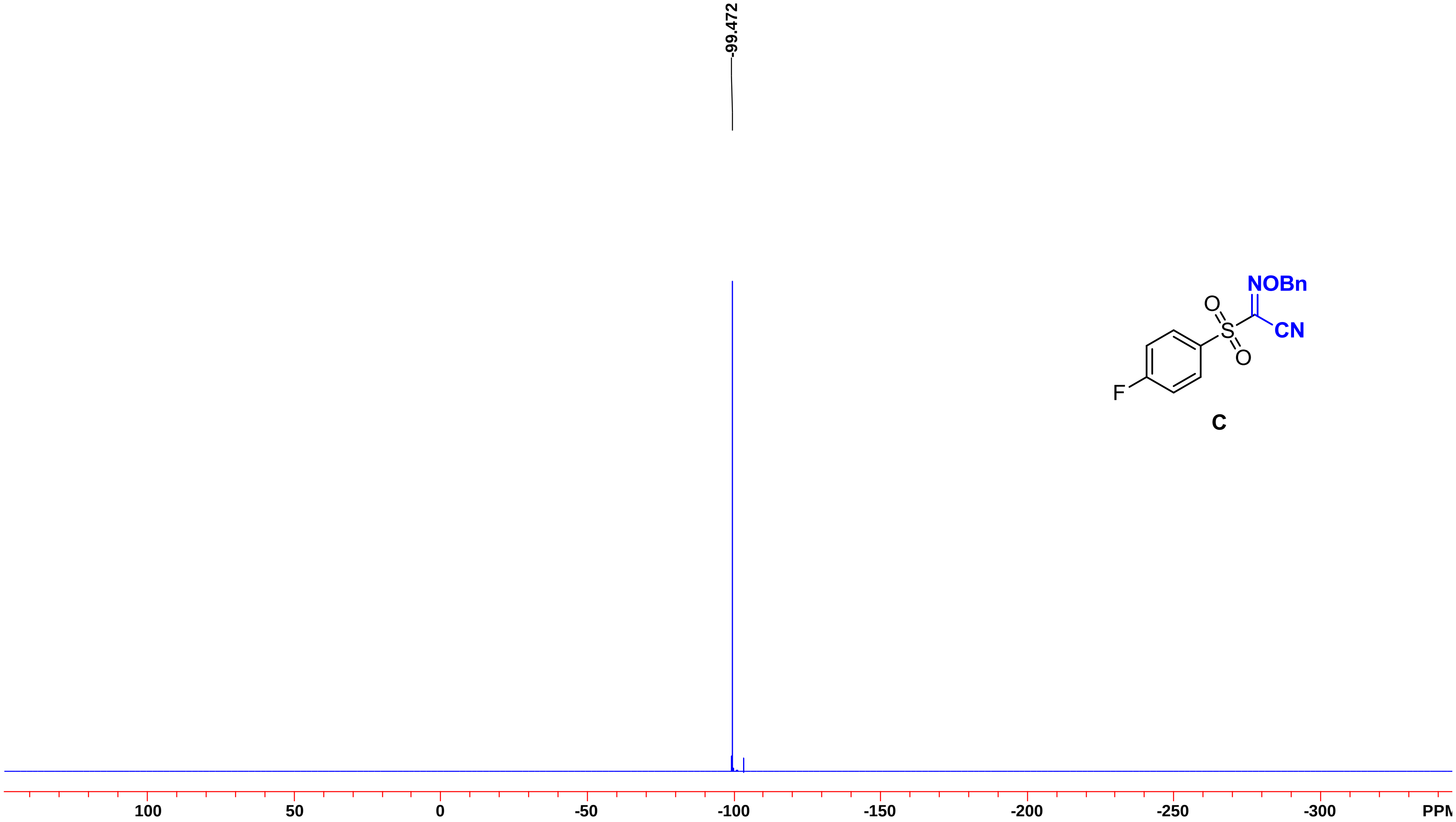

Supplementary Figure 7. ^{19}F NMR of **C**

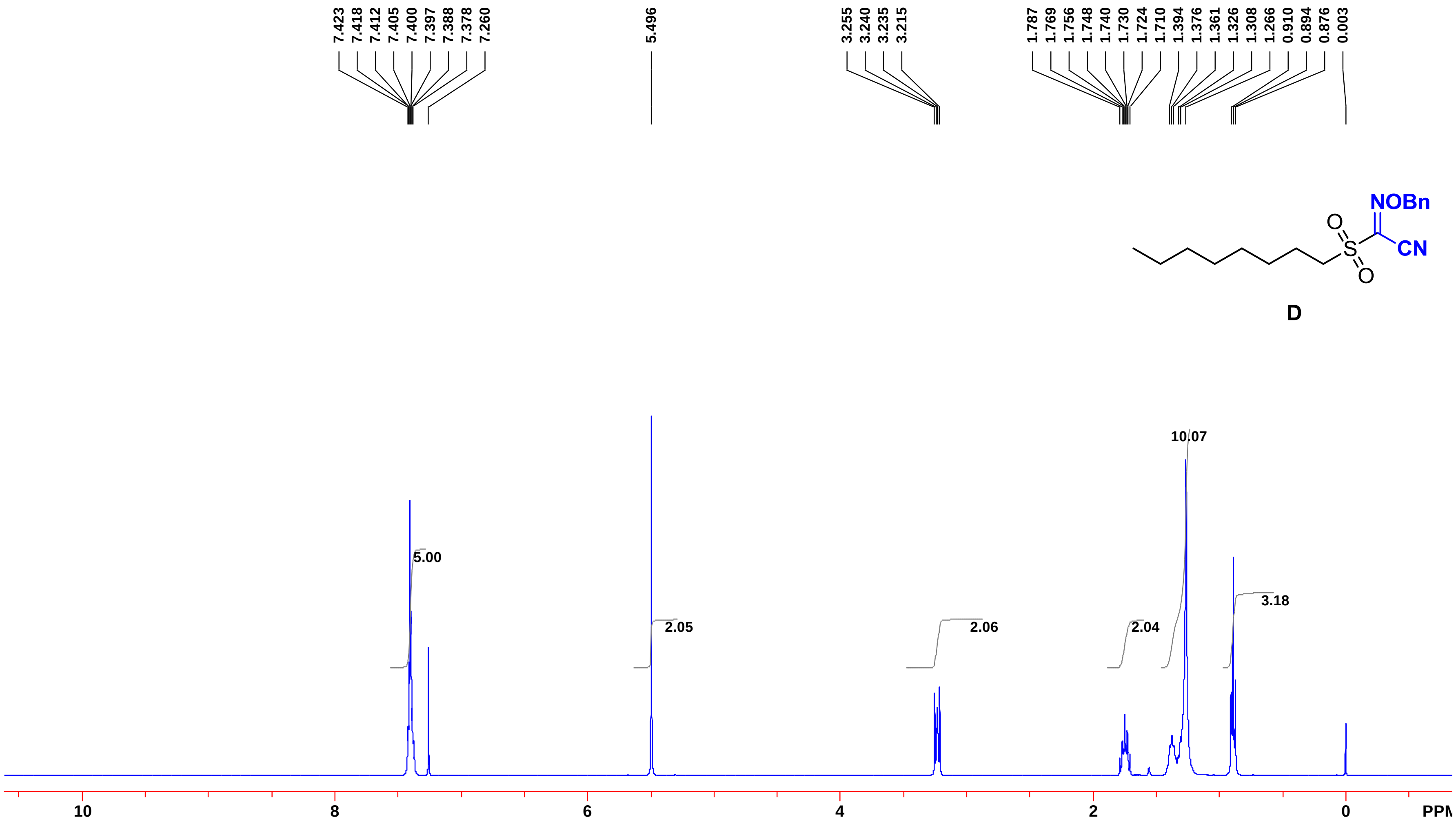

Supplementary Figure 8. ^{1}H NMR of **D**

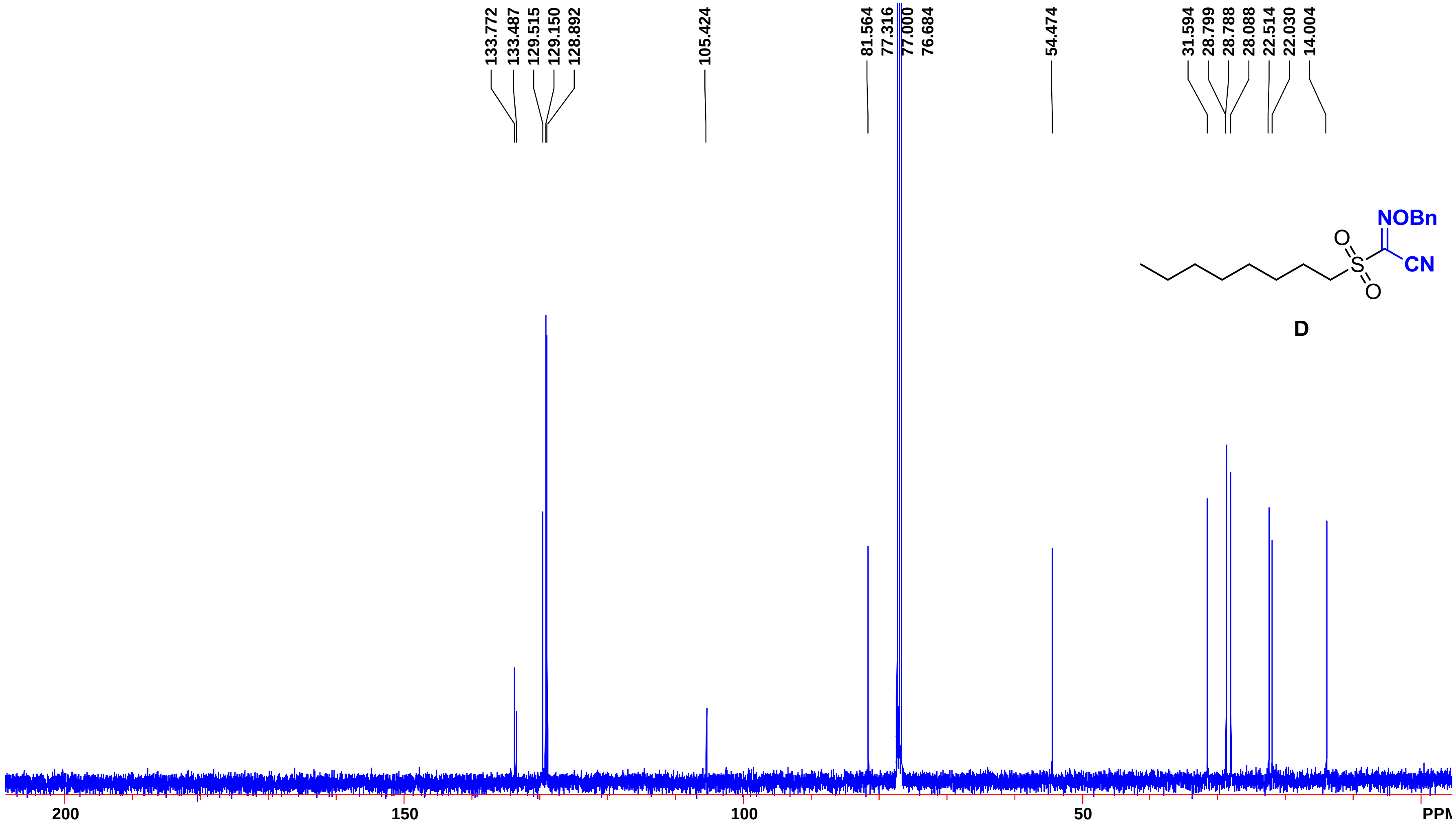

Supplementary Figure 9. ^{13}C NMR of **D**

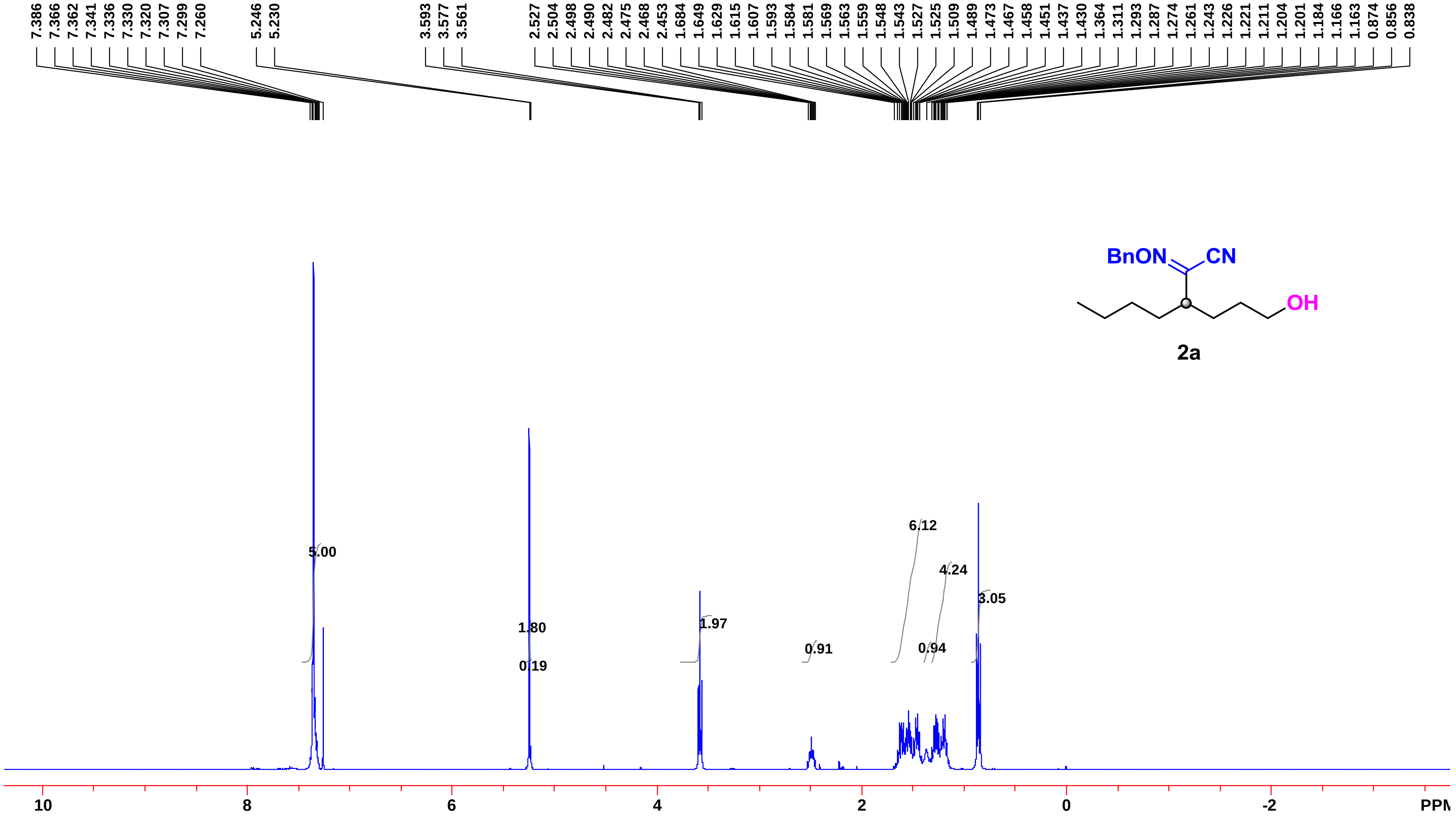

Supplementary Figure 10. ^{1}H NMR of **2a**

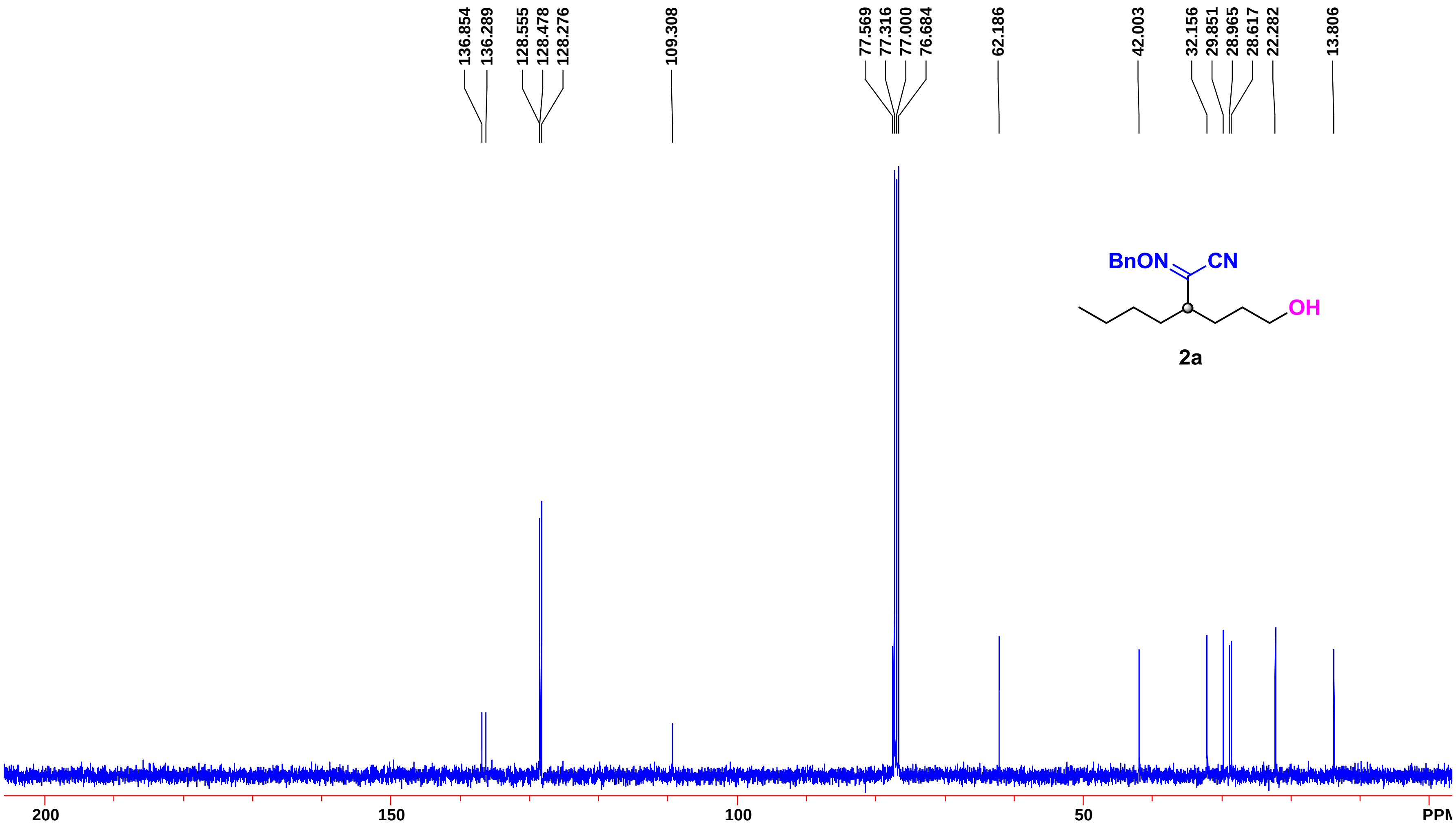

Supplementary Figure 11. ^{13}C NMR of **2a**

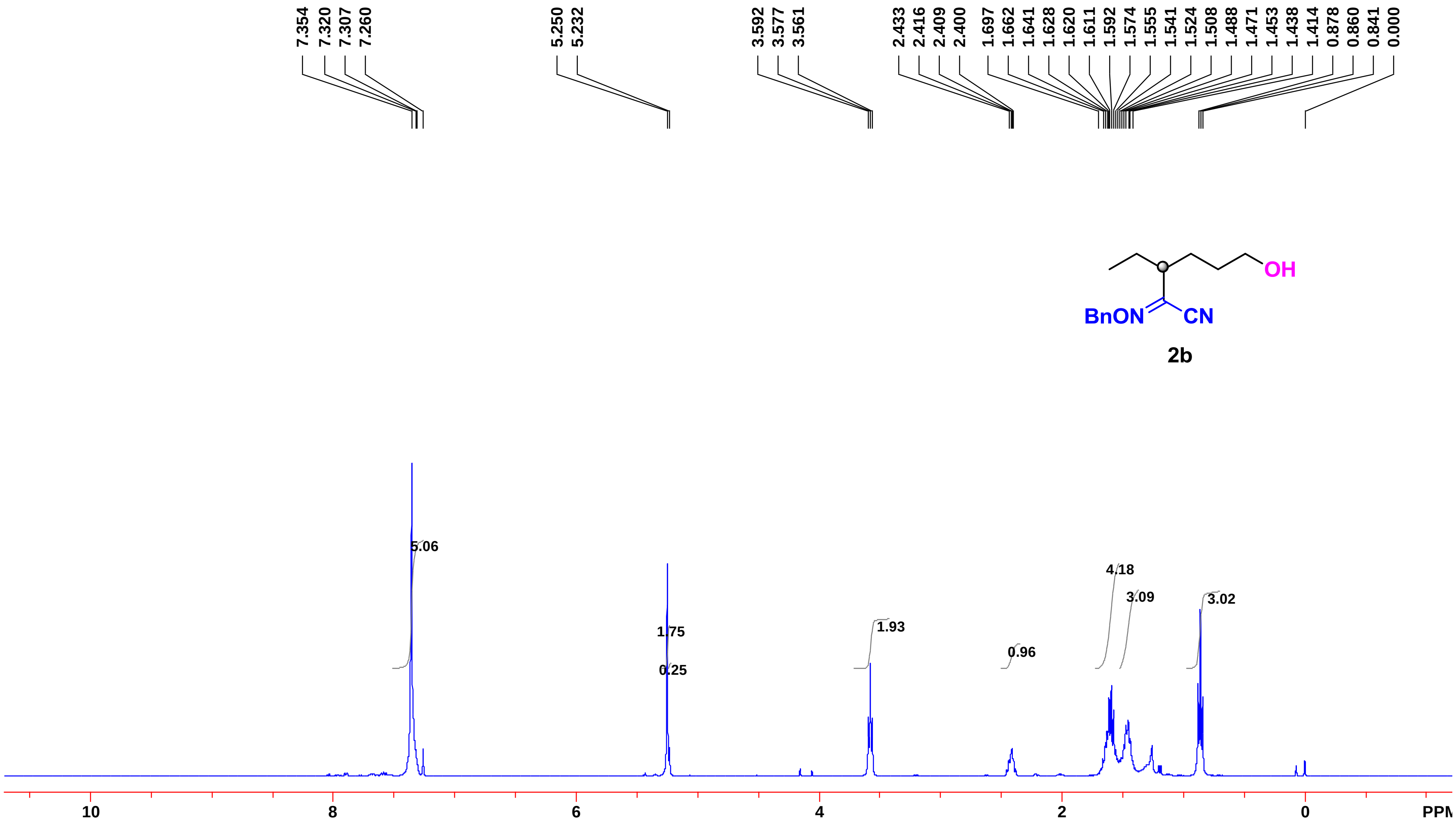

Supplementary Figure 12. ^{1}H NMR of **2b**

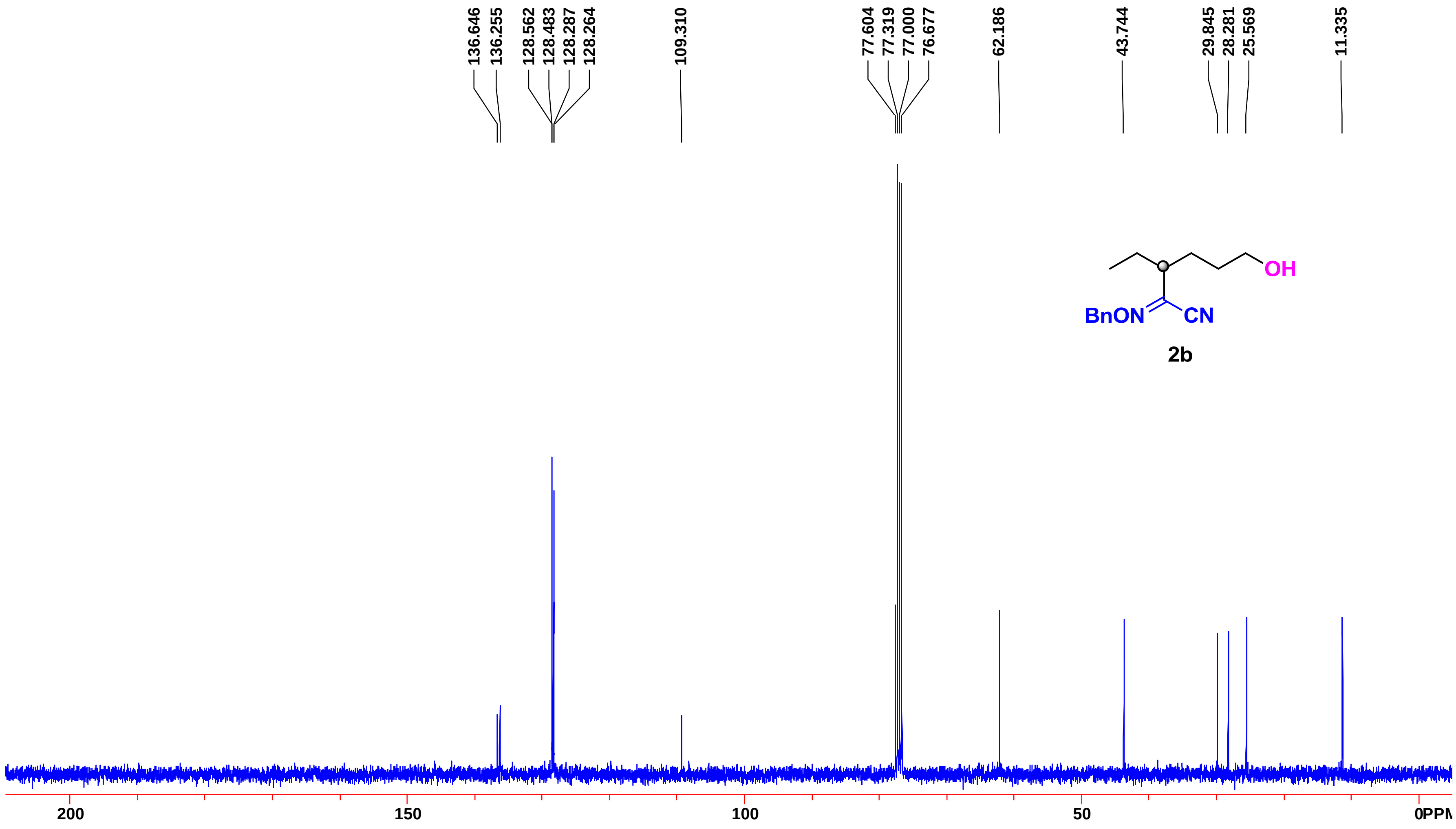

Supplementary Figure 13. ^{13}C NMR of **2b**

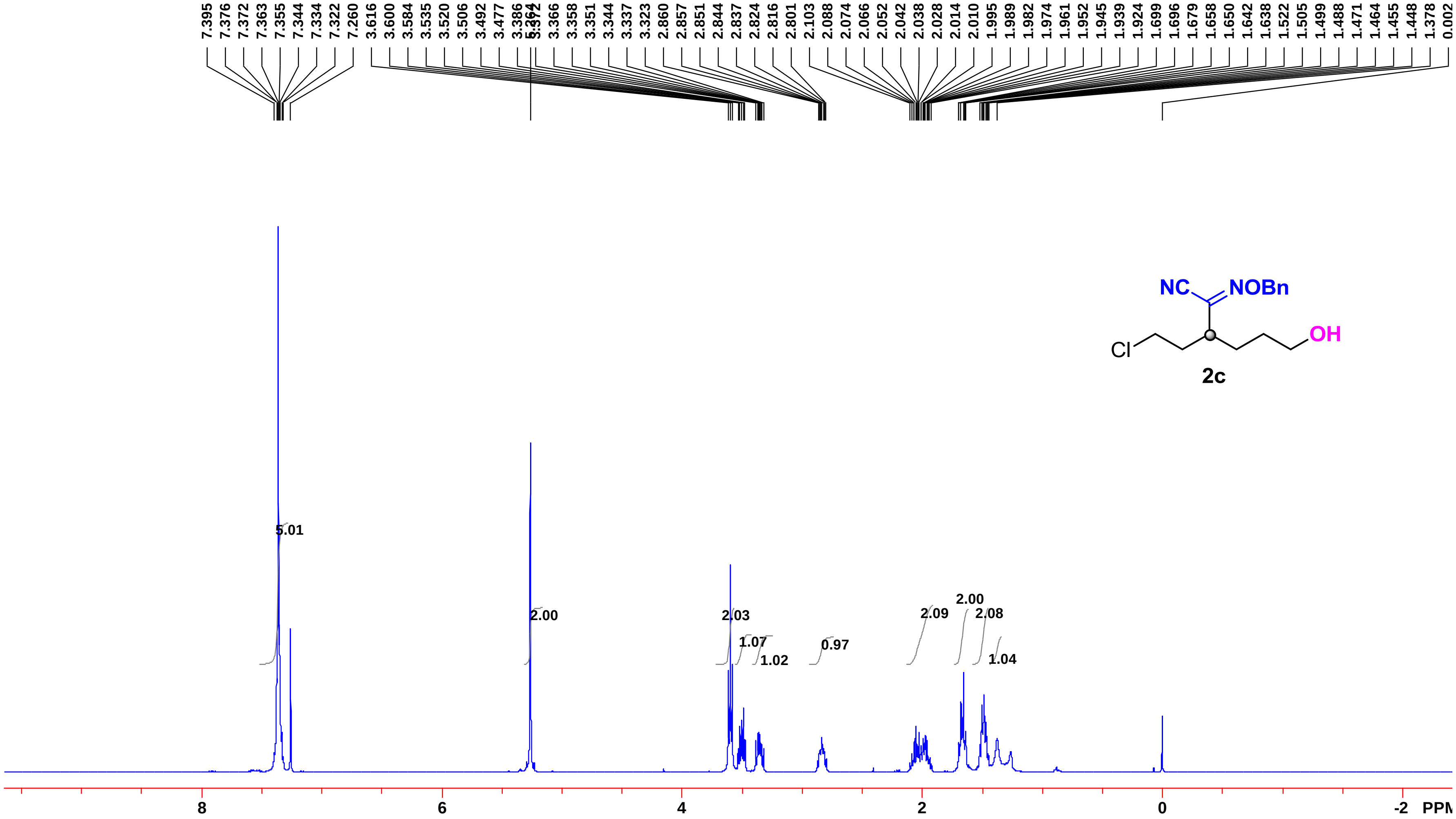

Supplementary Figure 14. ^{1}H NMR of **2c**

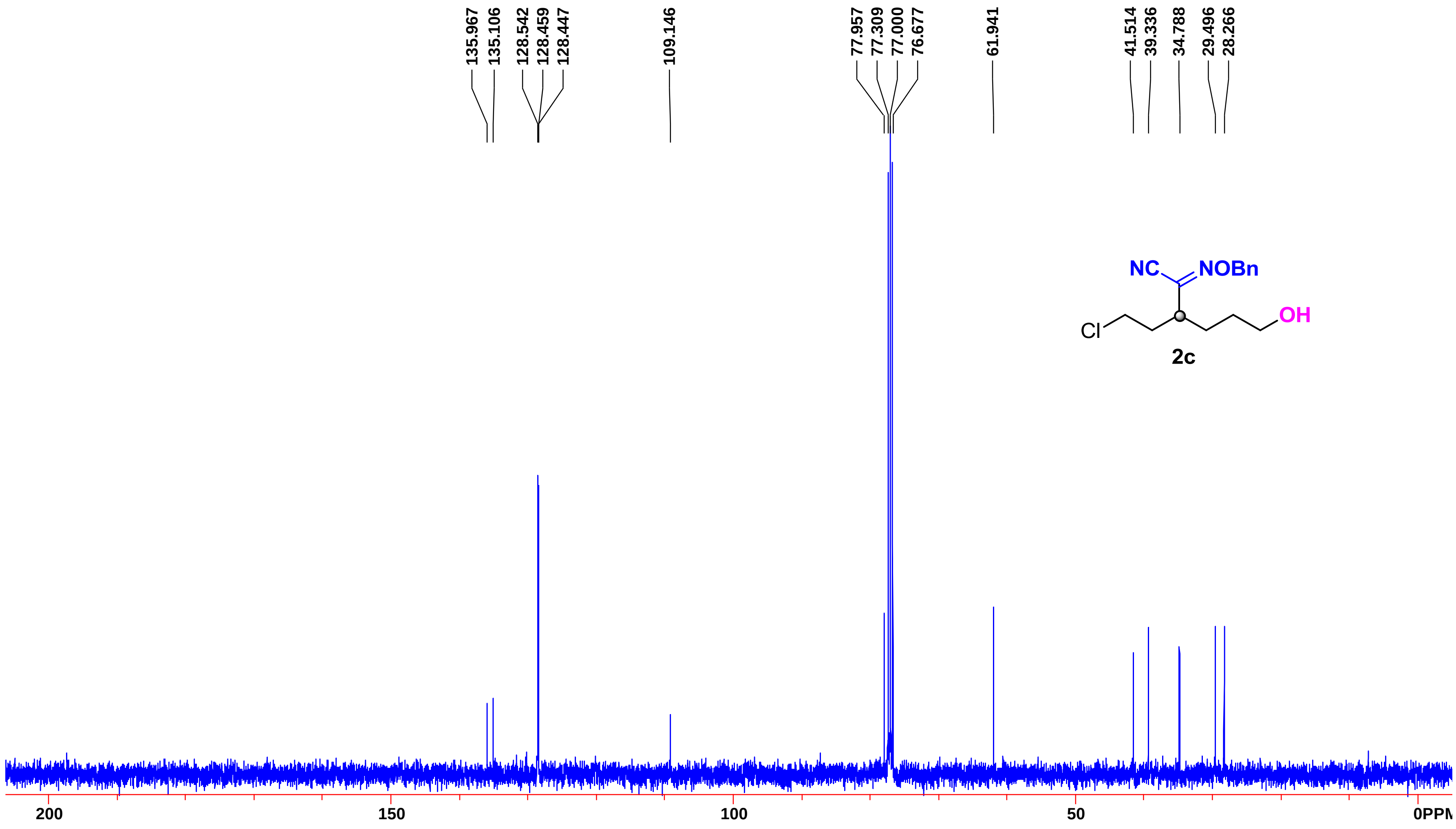

Supplementary Figure 15. ^{13}C NMR of **2c**

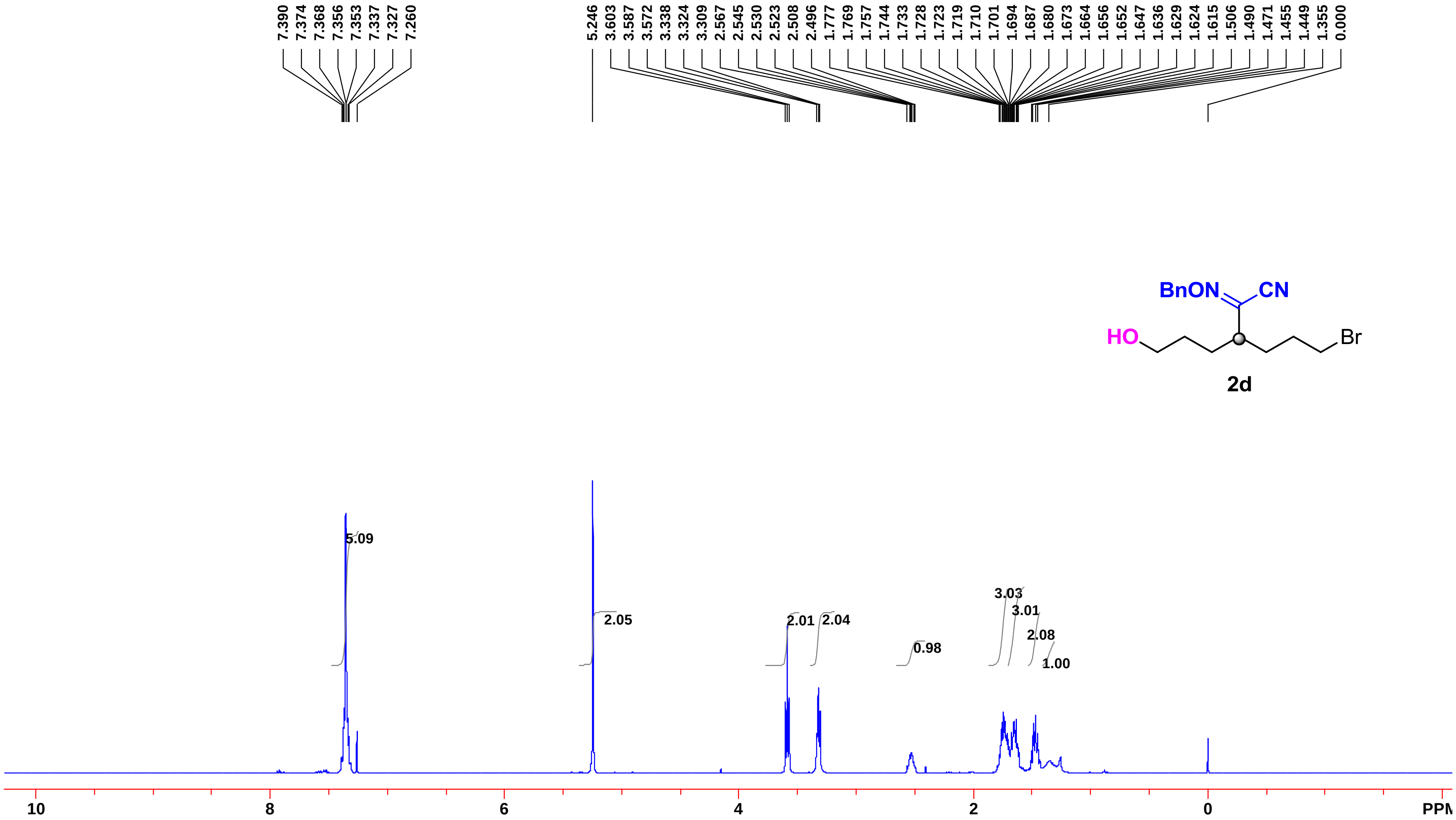

Supplementary Figure 16. ^{1}H NMR of **2d**

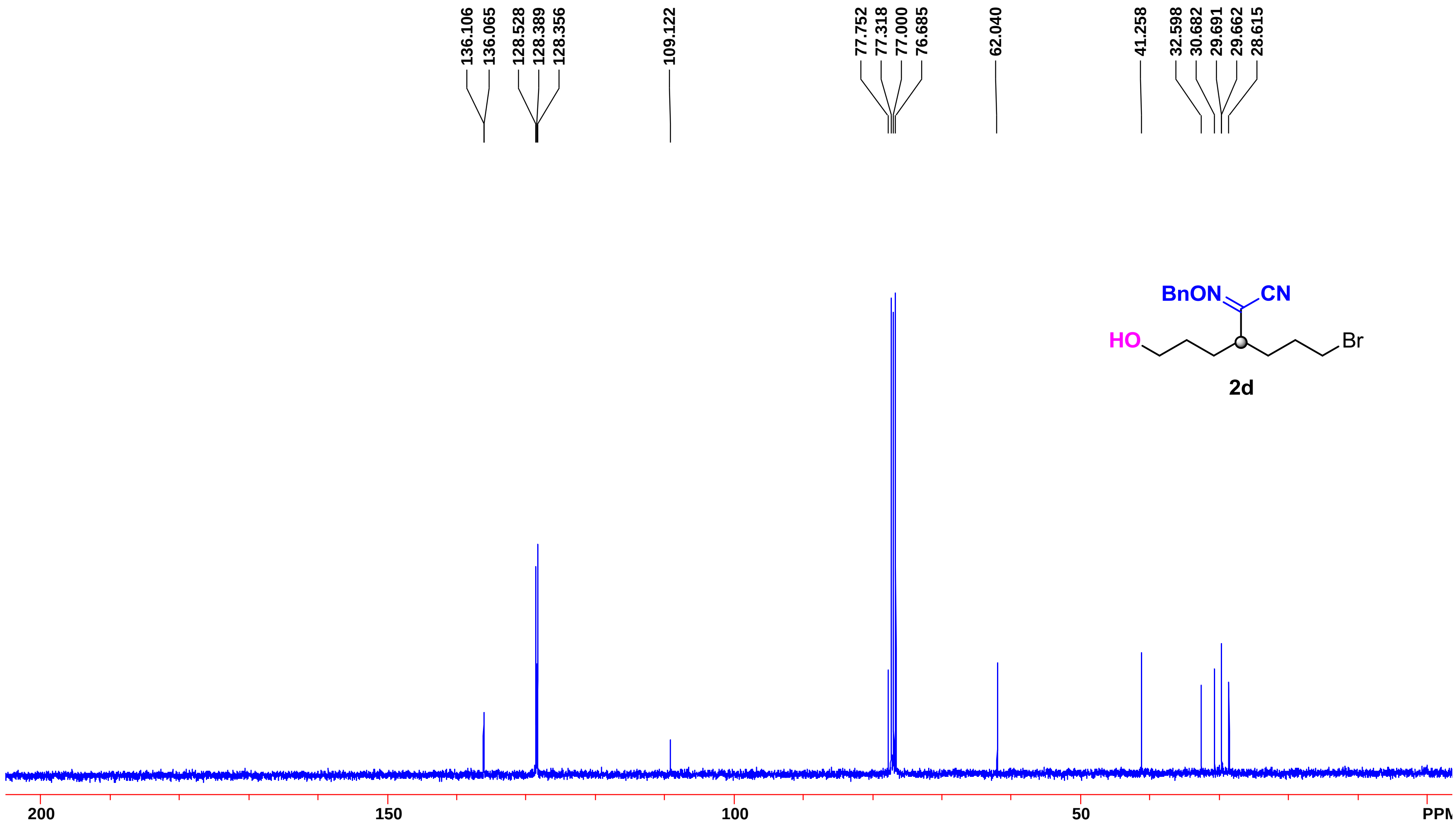

Supplementary Figure 17. ^{13}C NMR of **2d**

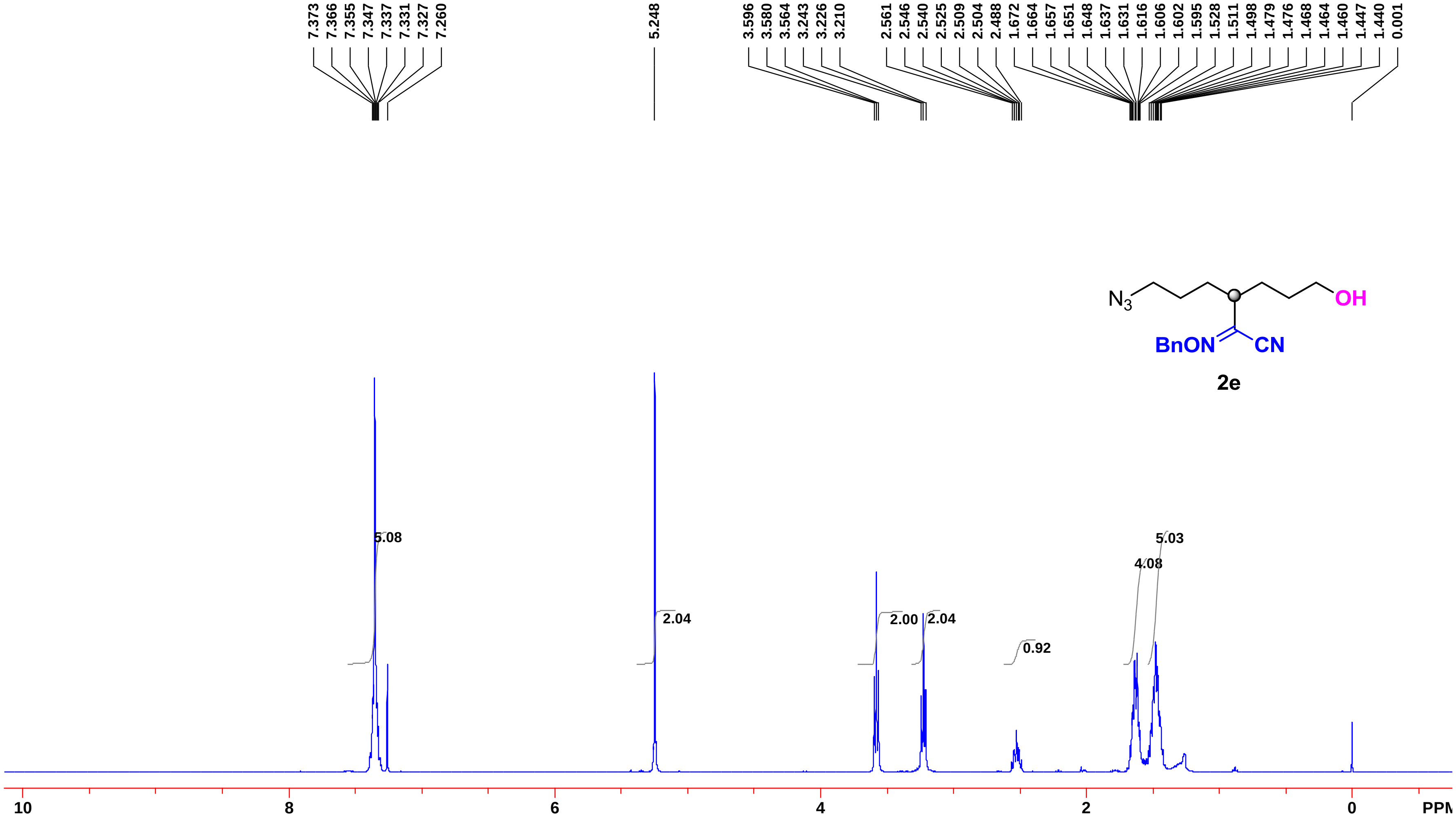

Supplementary Figure 18. ^{1}H NMR of **2e**

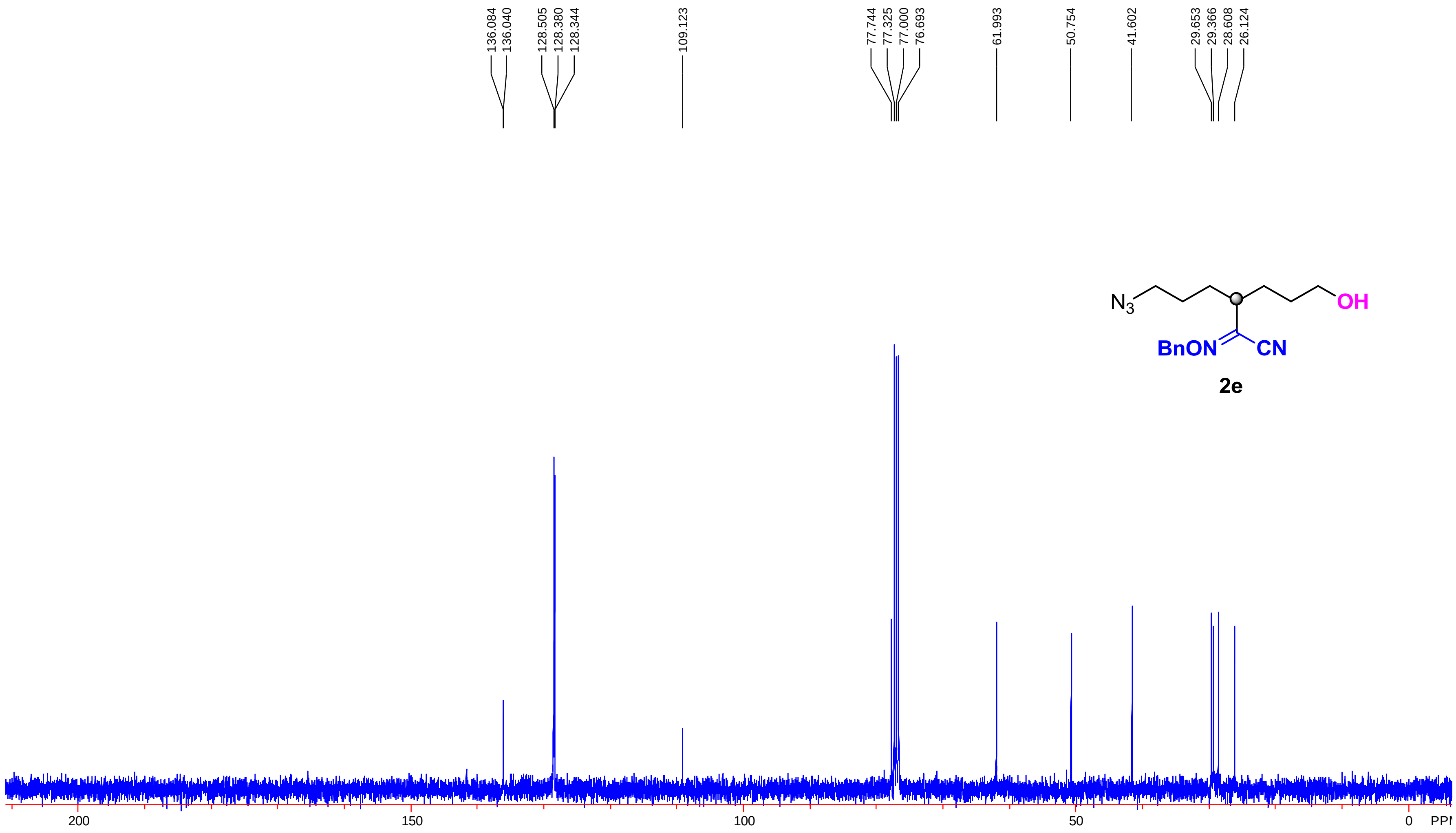

Supplementary Figure 19. ^{13}C NMR of **2e**

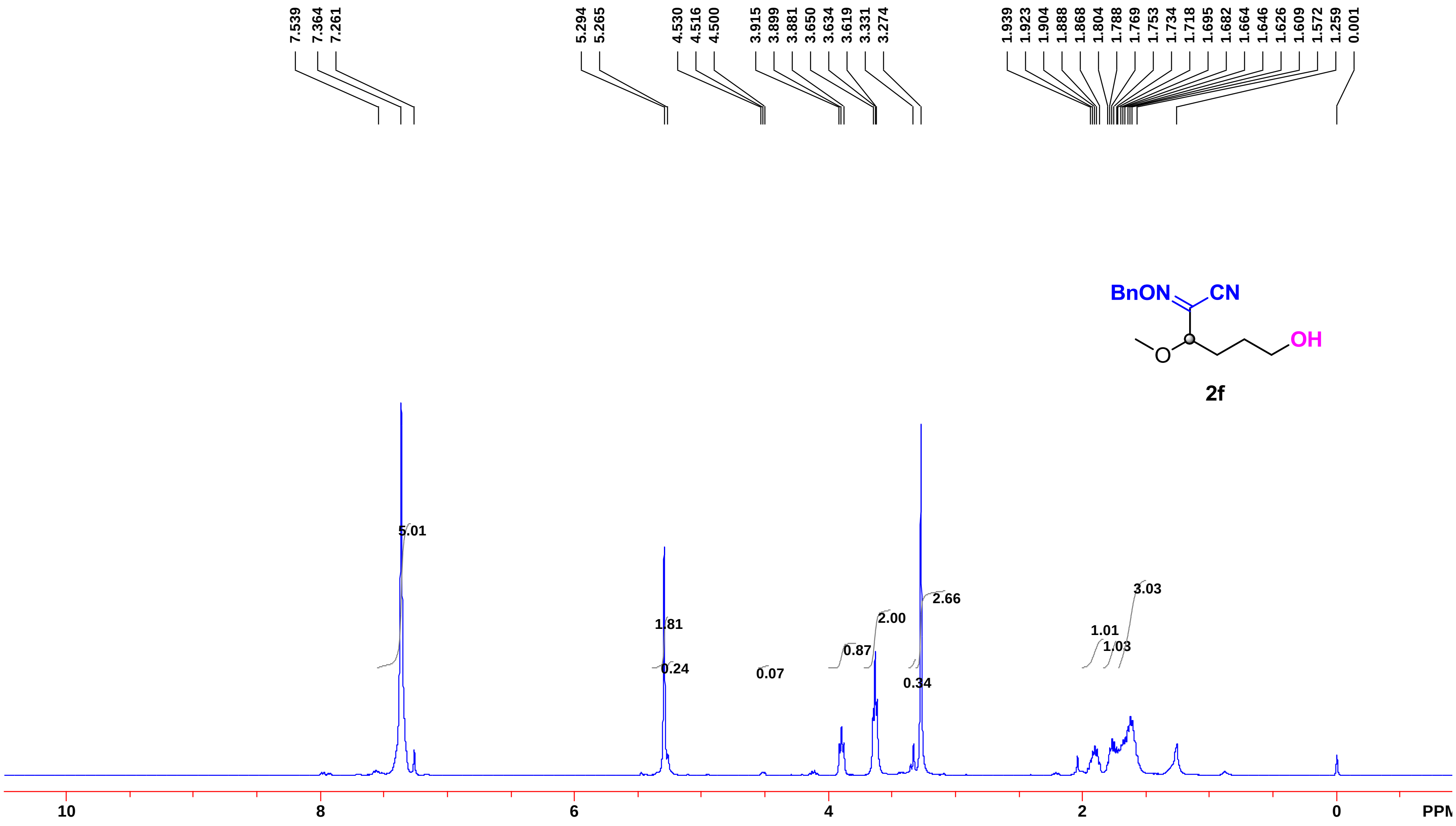

Supplementary Figure 20. ^{1}H NMR of **2f**

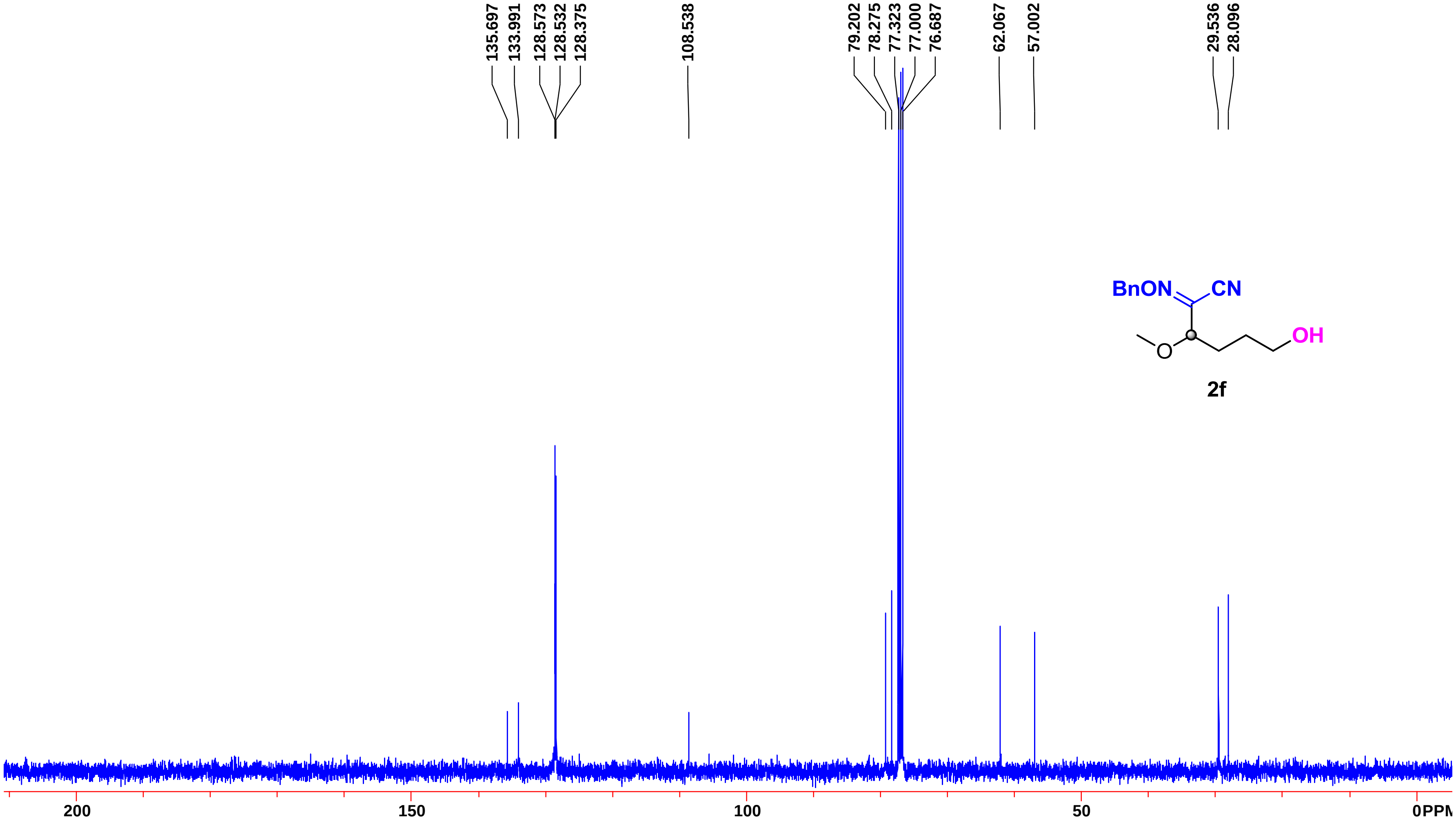

2f

Supplementary Figure 21. ^{13}C NMR of **2f**

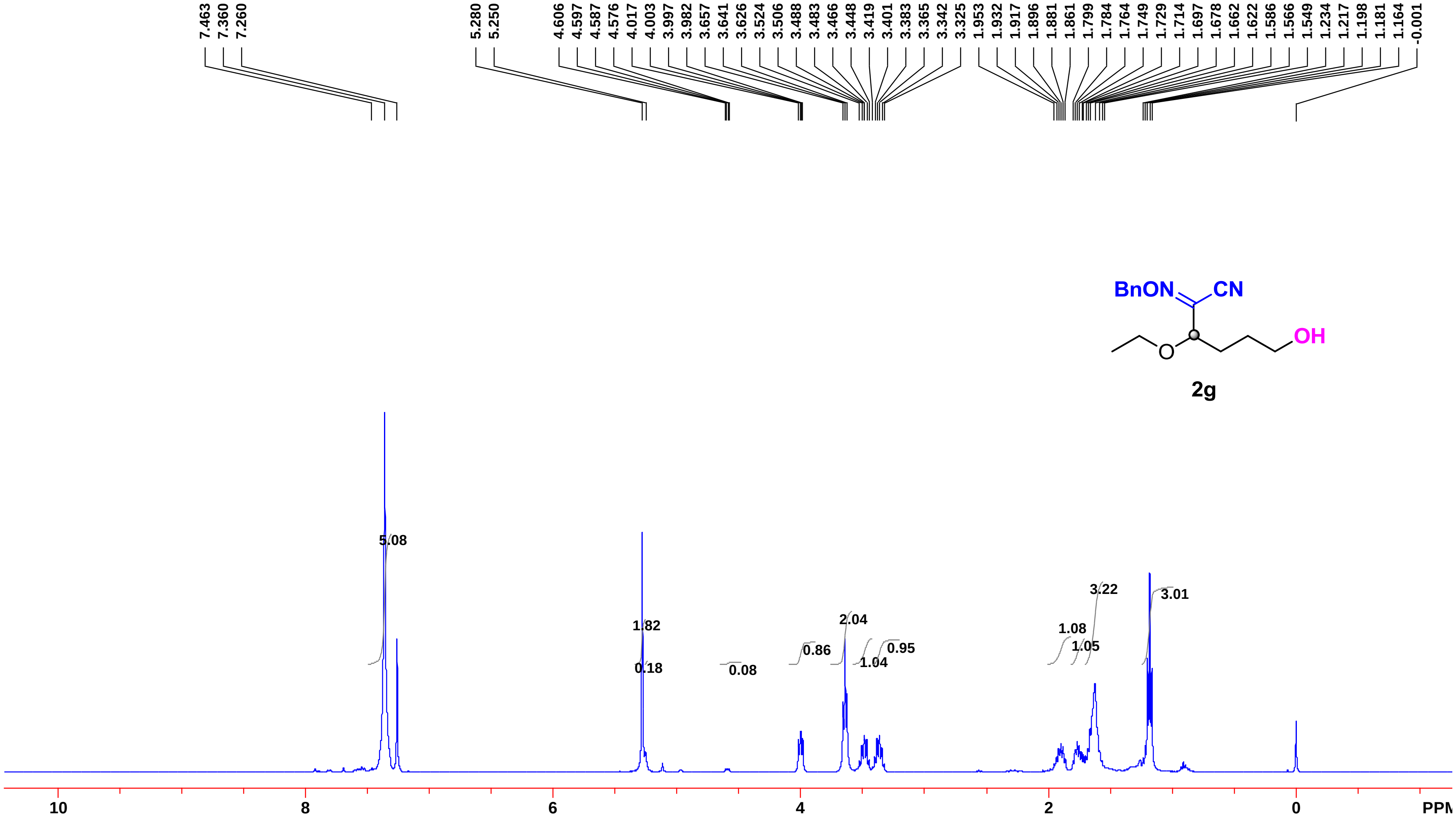

Supplementary Figure 22. ^{1}H NMR of **2g**

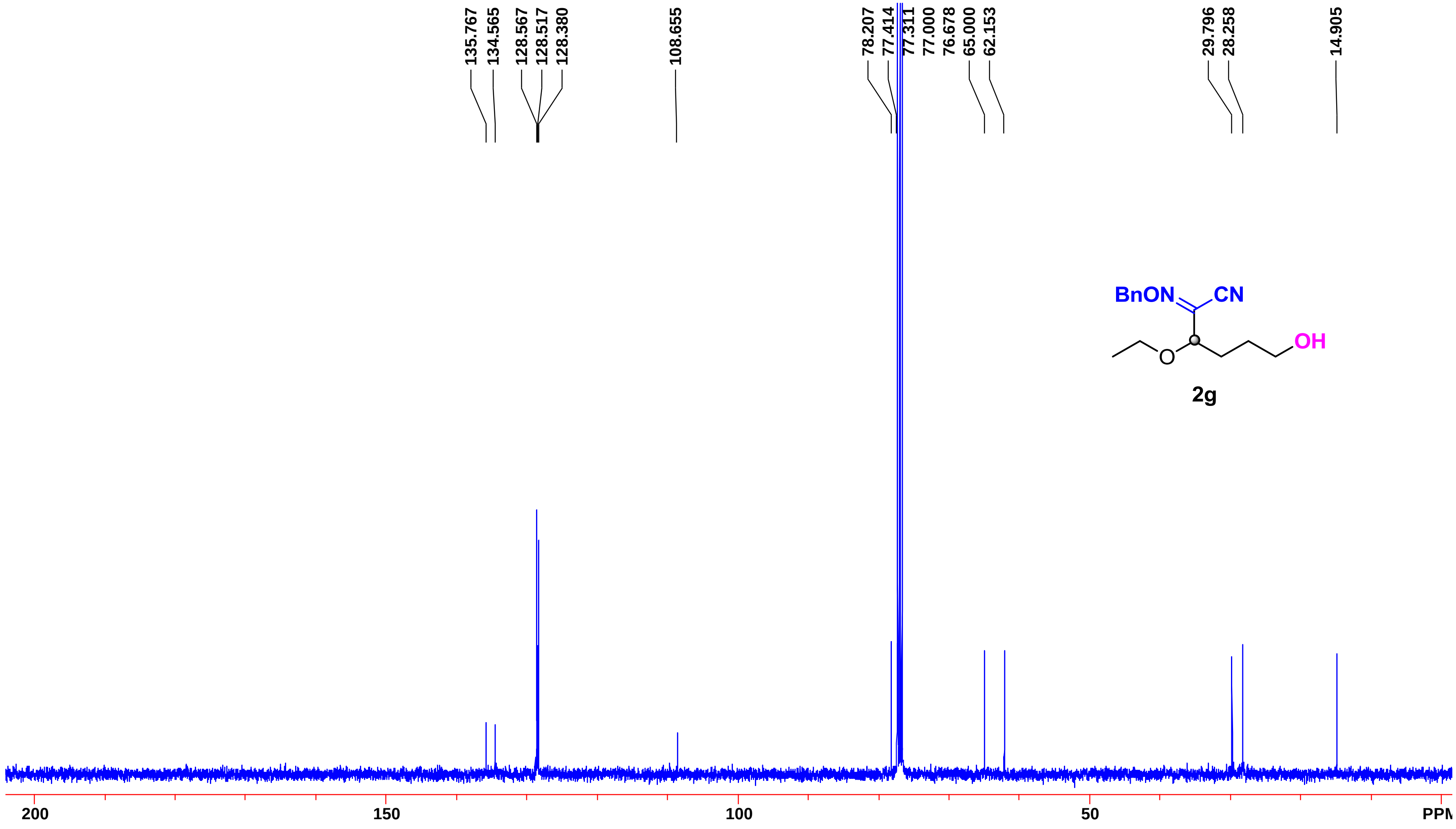

Supplementary Figure 23. ^{13}C NMR of **2g**

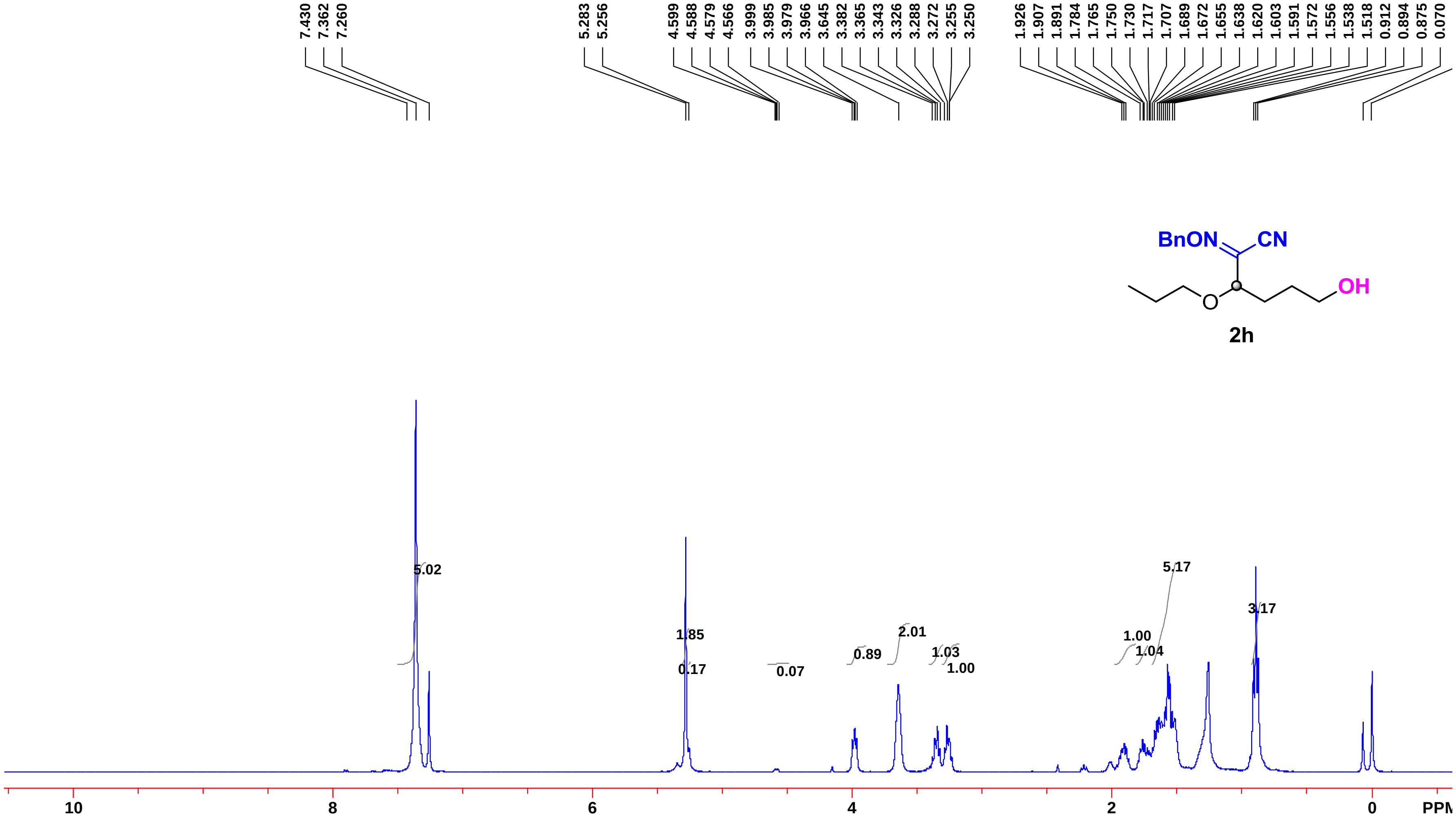

Supplementary Figure 24. ^{1}H NMR of **2h**

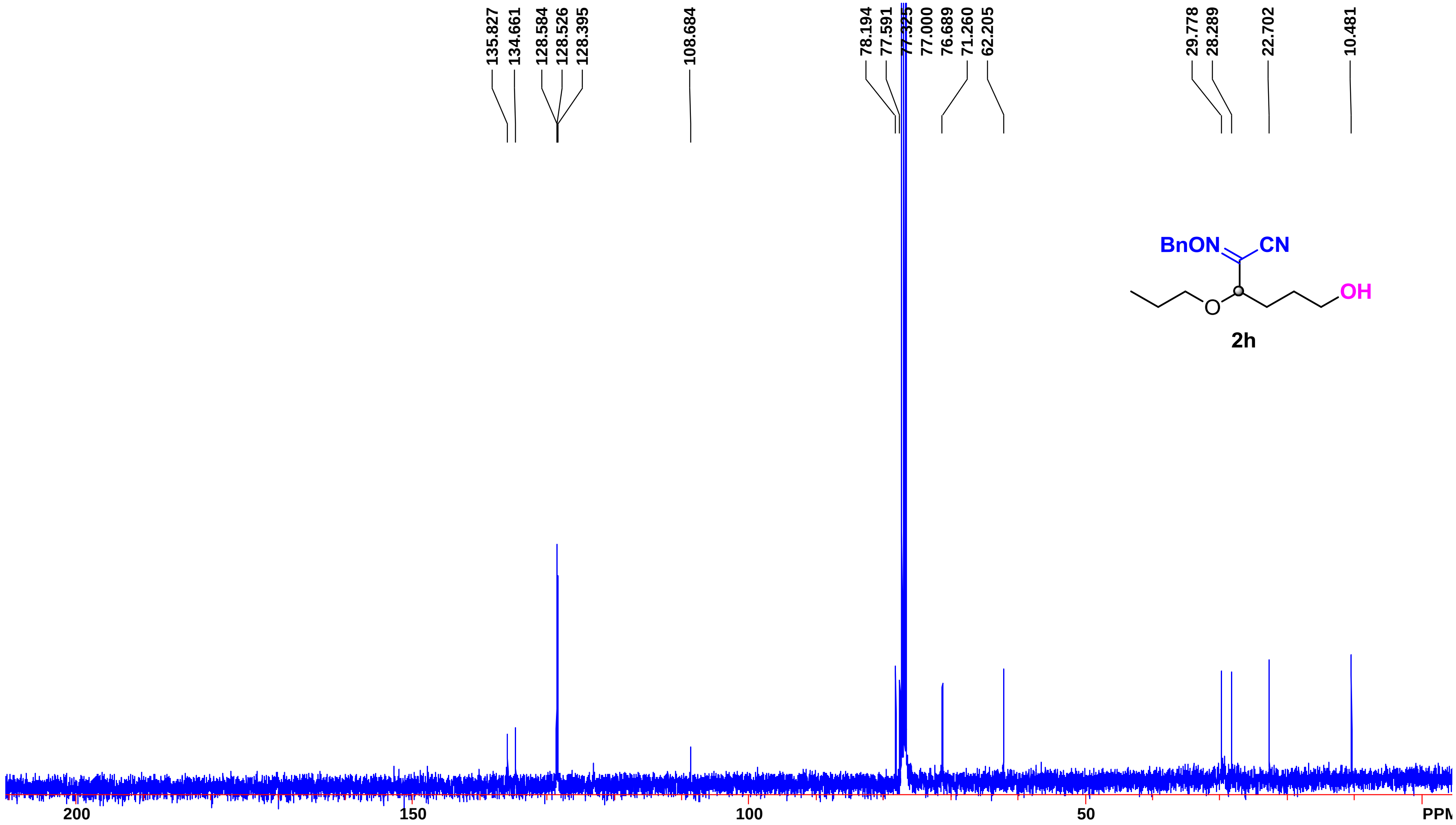

Supplementary Figure 25. ^{13}C NMR of **2h**

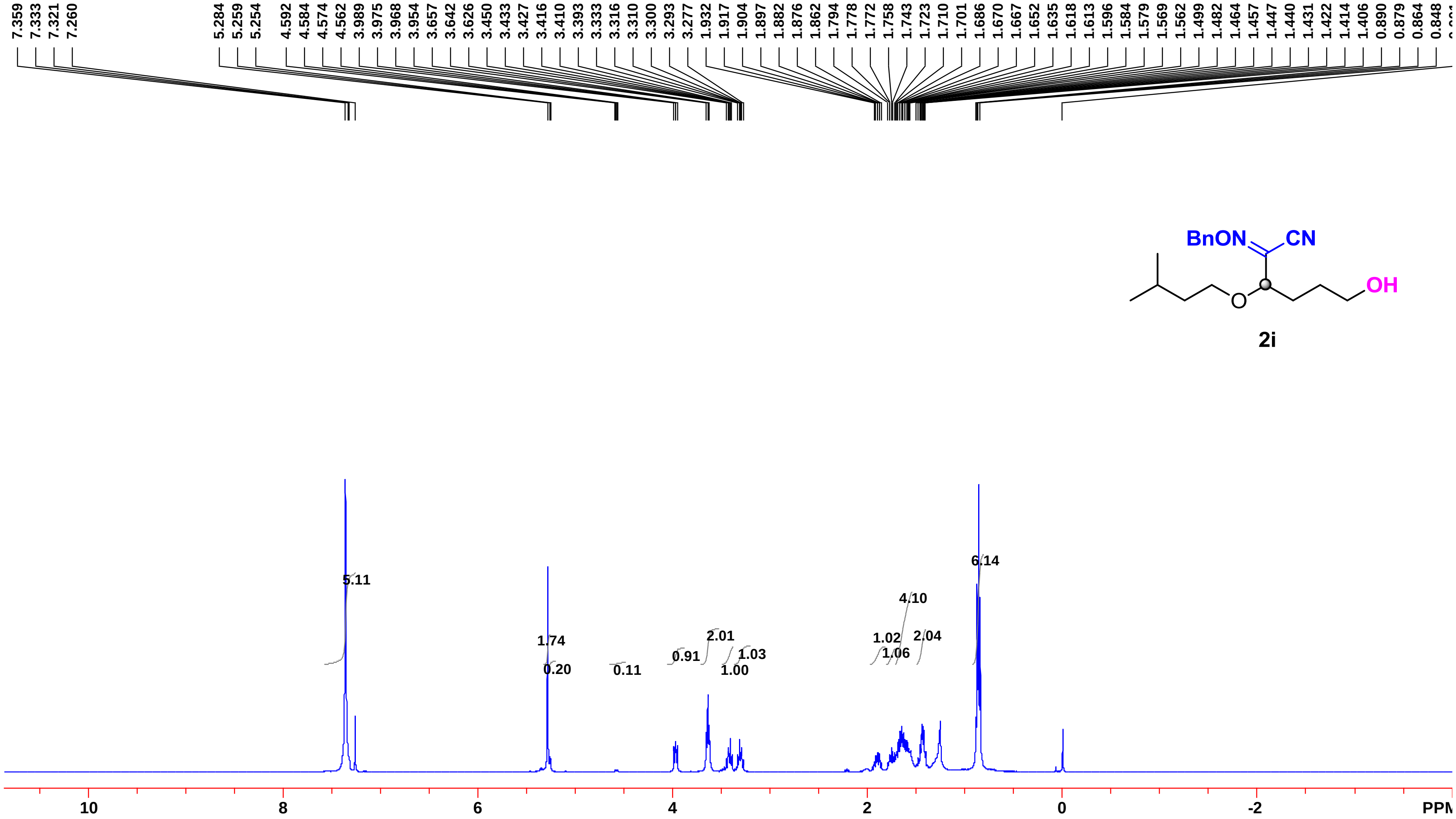

Supplementary Figure 26. ^{1}H NMR of **2i**

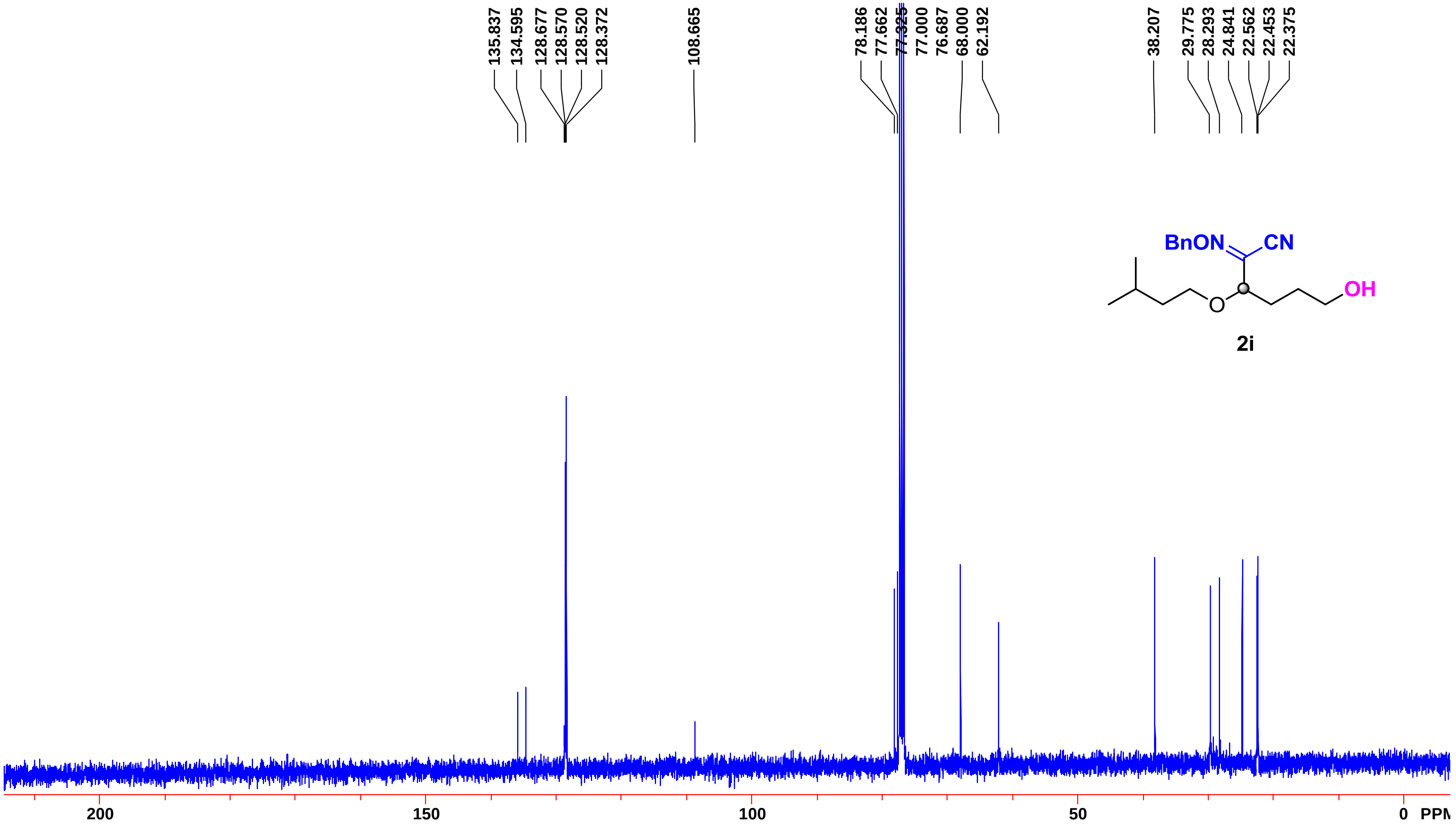

Supplementary Figure 27. ^{13}C NMR of **2i**

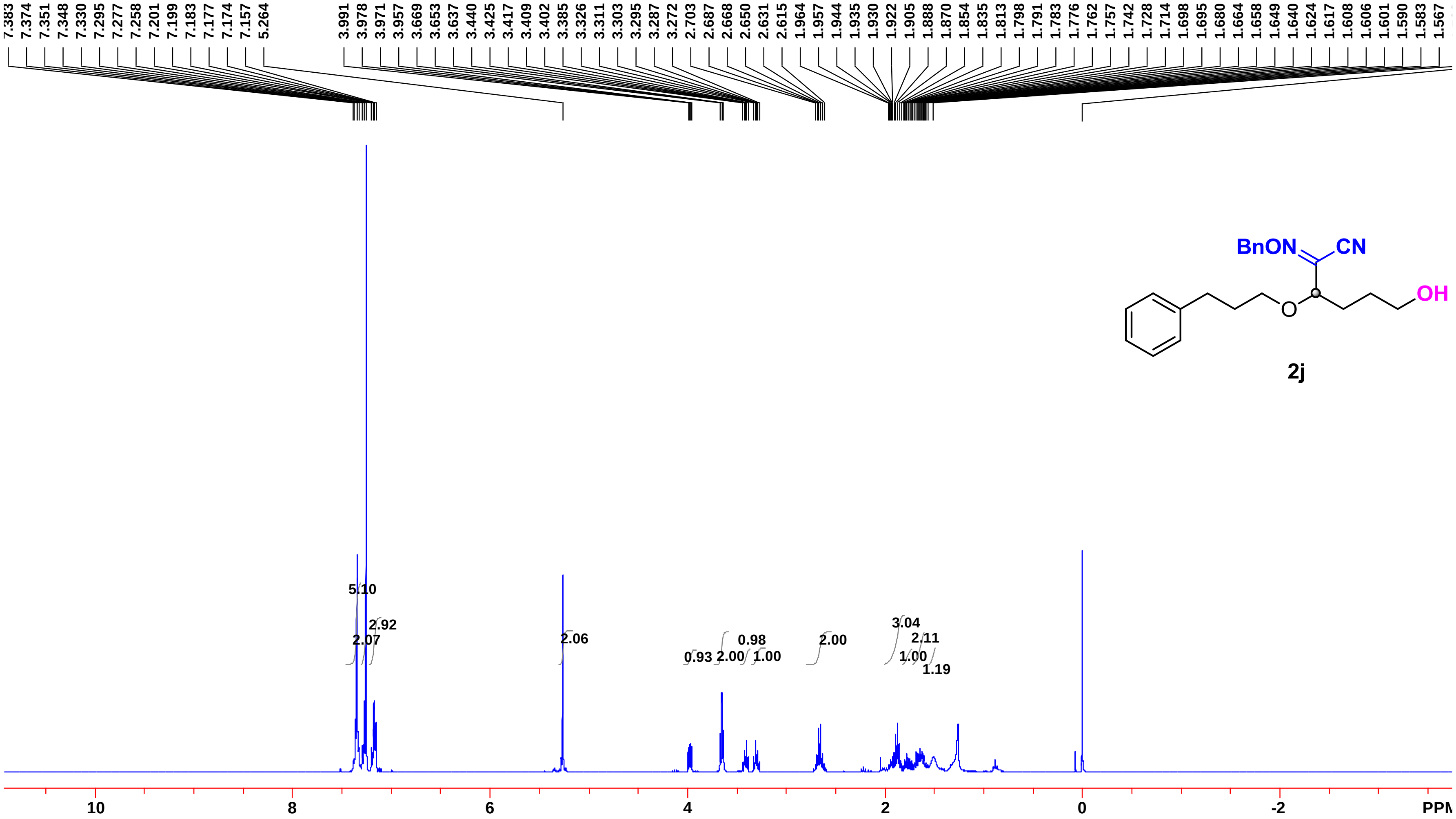

Supplementary Figure 28. ^{1}H NMR of **2j**

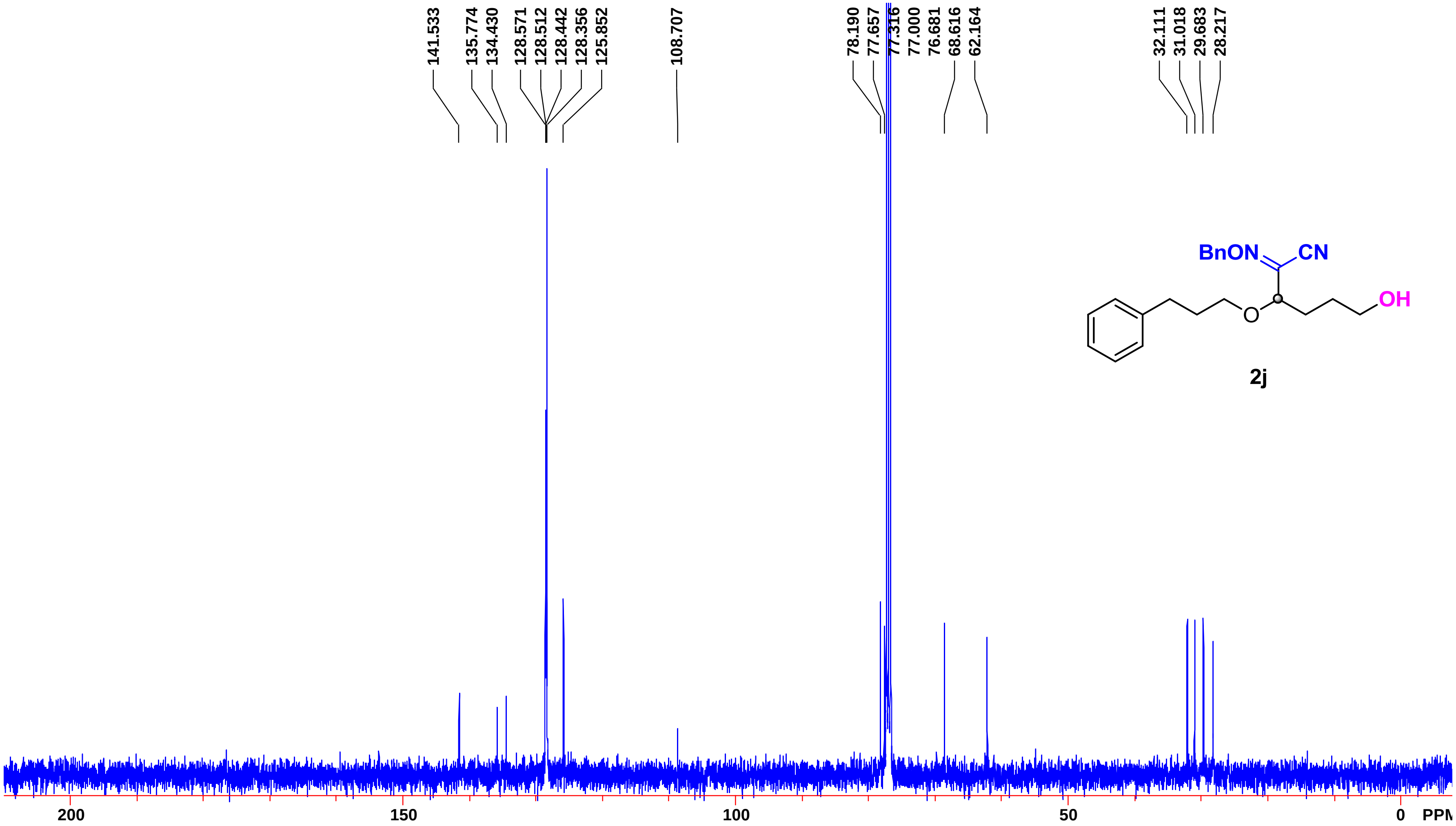

Supplementary Figure 29. ^{13}C NMR of **2j**

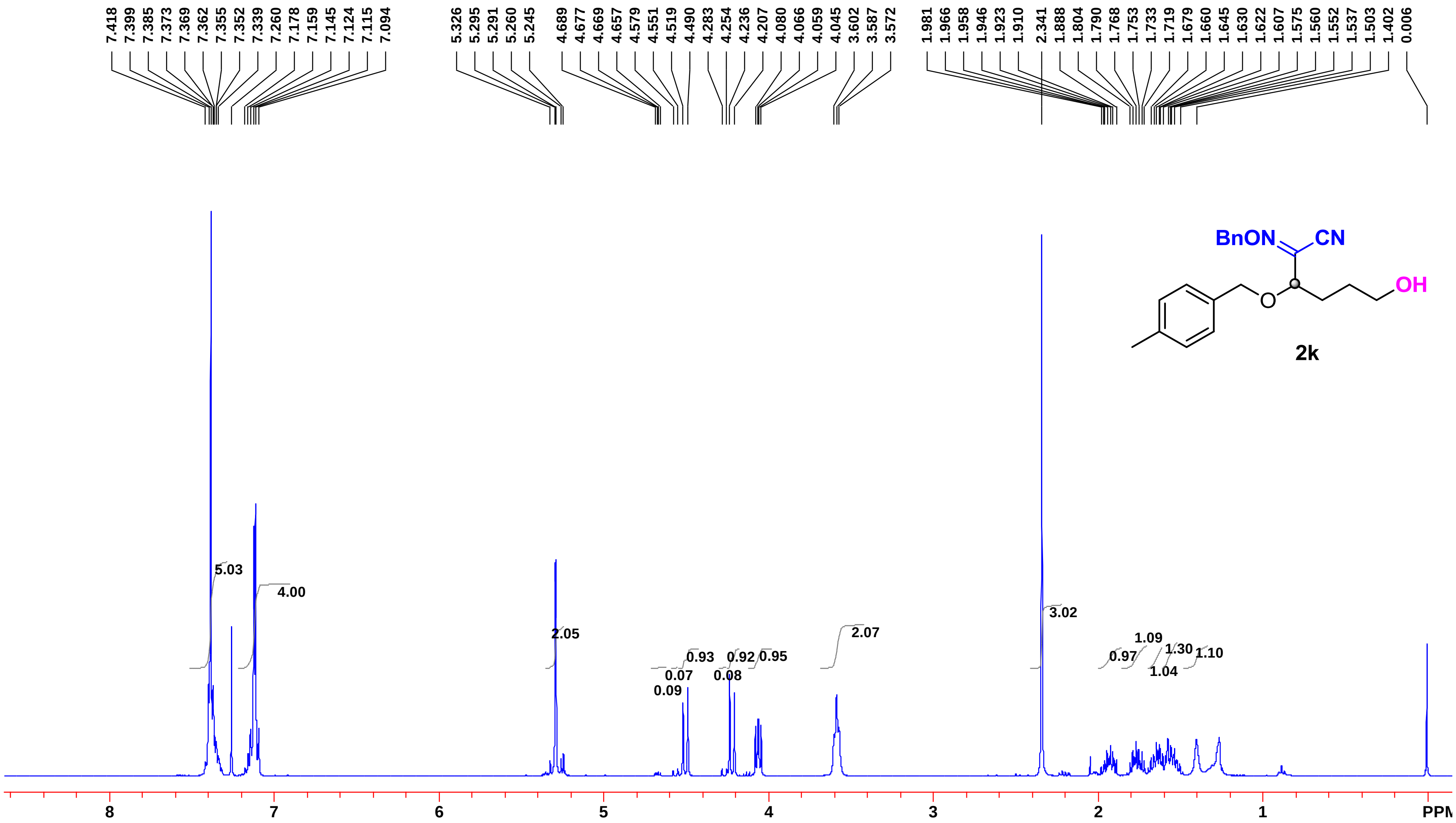

Supplementary Figure 30. ^{1}H NMR of **2k**

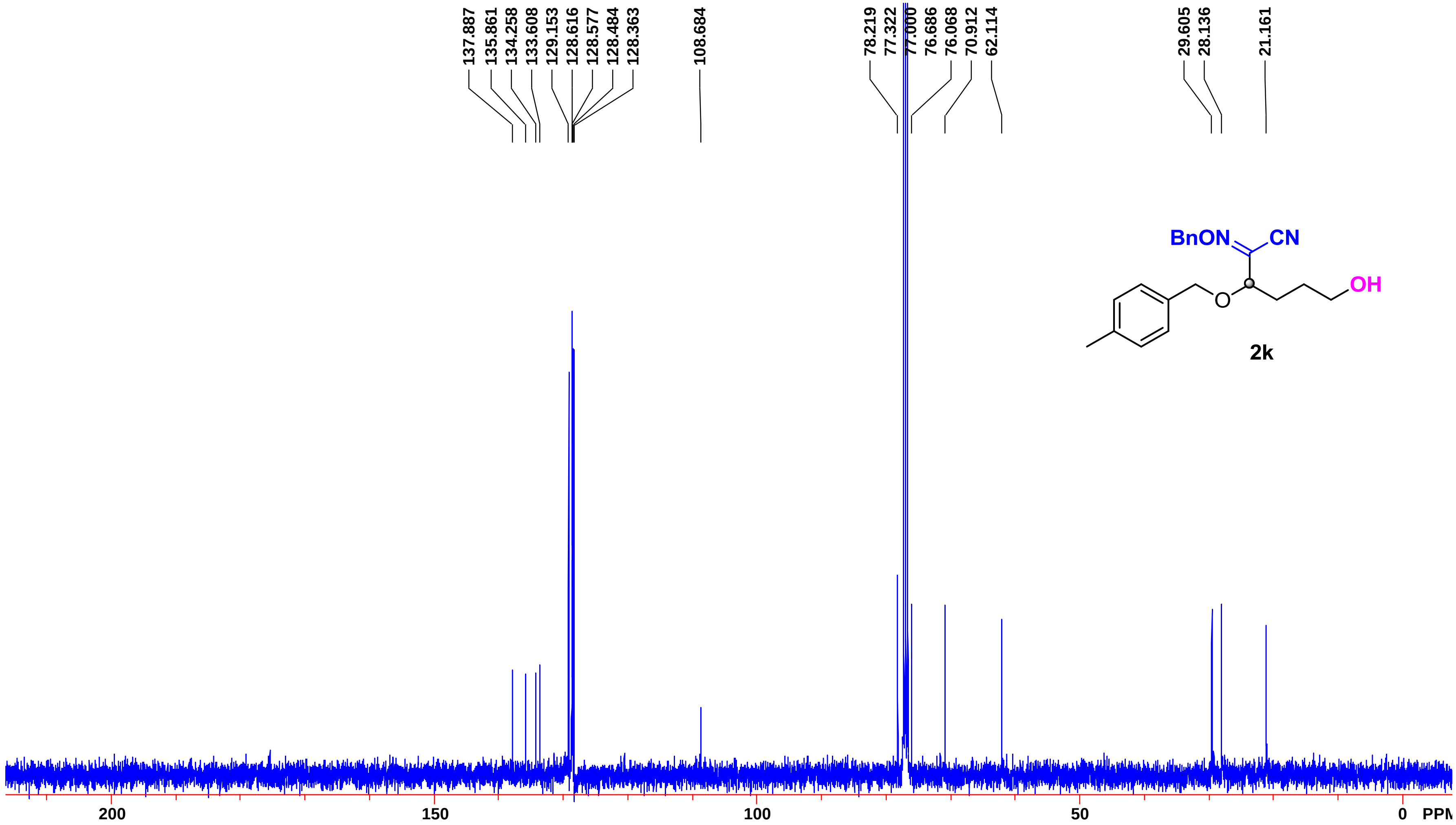

Supplementary Figure 31. ^{13}C NMR of **2k**

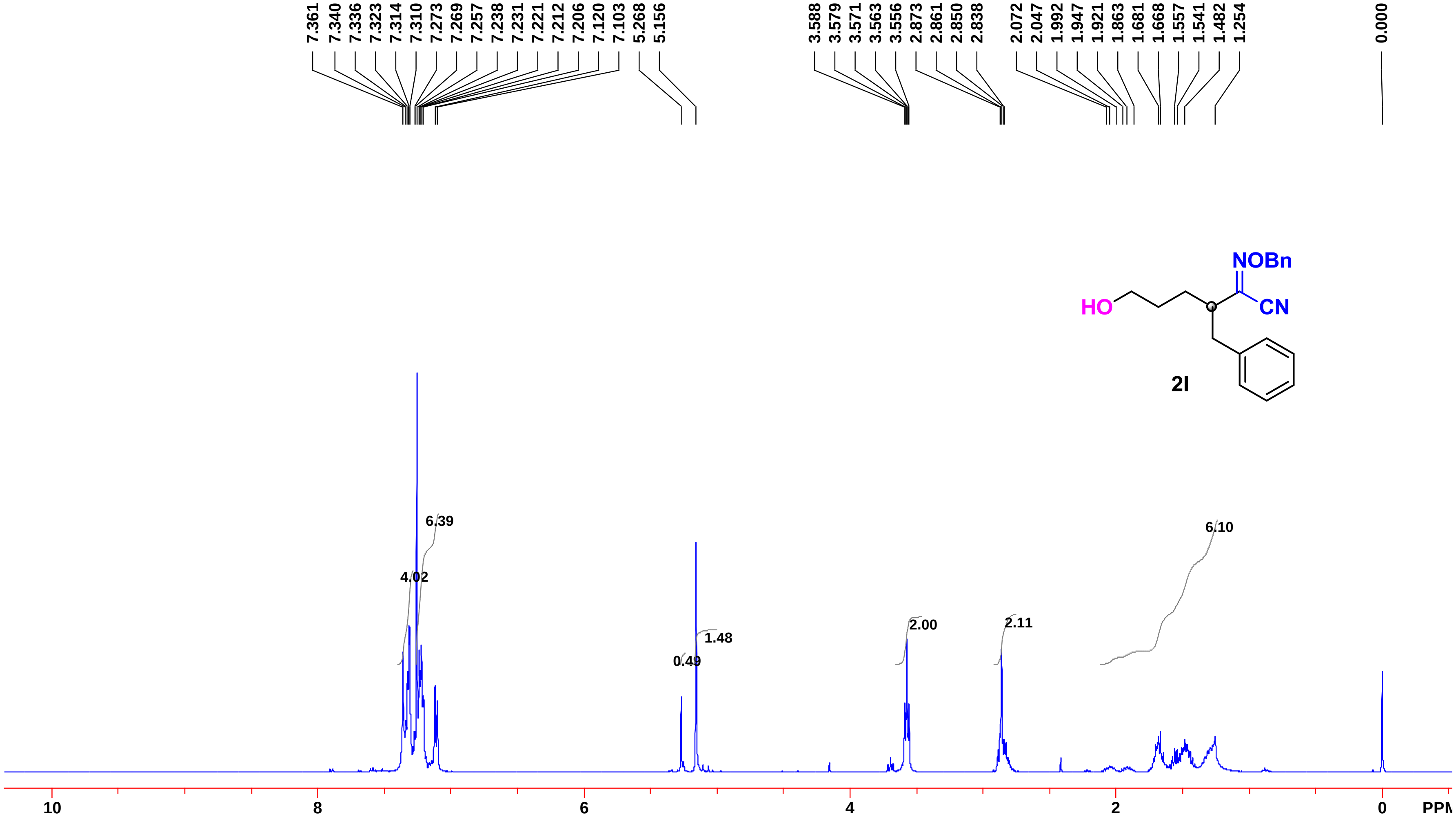

Supplementary Figure 32. ^{1}H NMR of **2l**

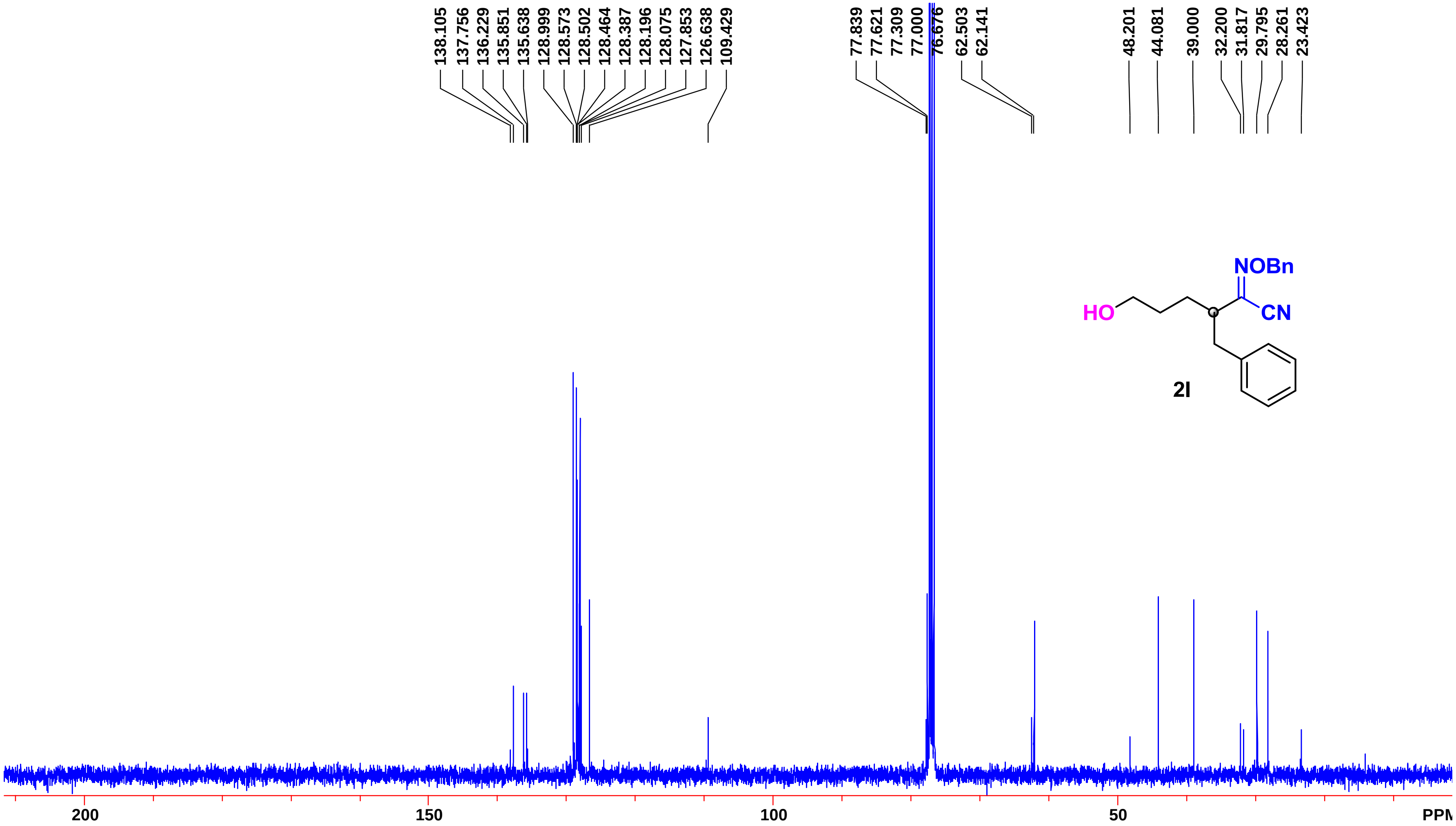

Supplementary Figure 33. ^{13}C NMR of **2l**

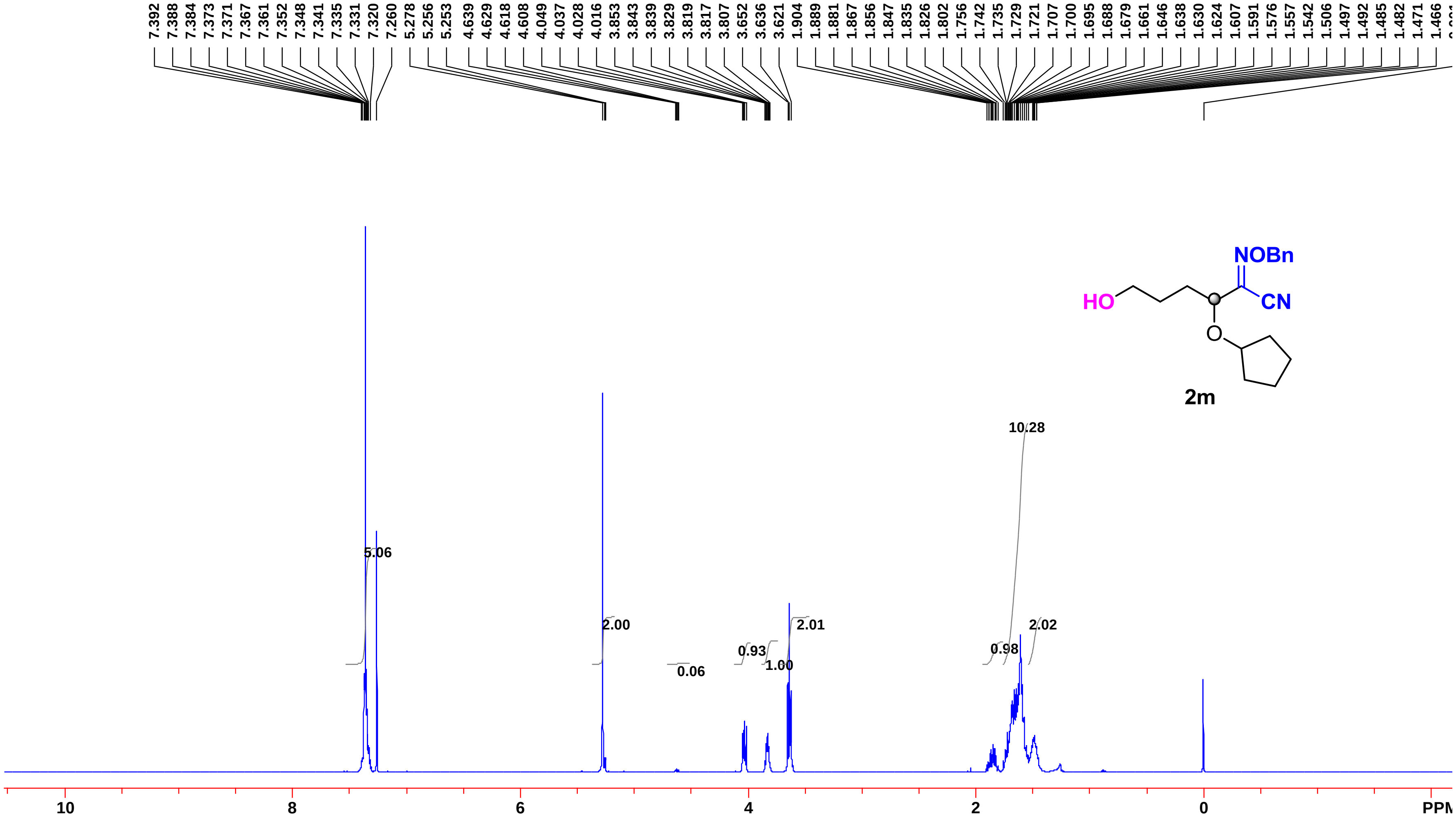

Supplementary Figure 34. ^{1}H NMR of **2m**

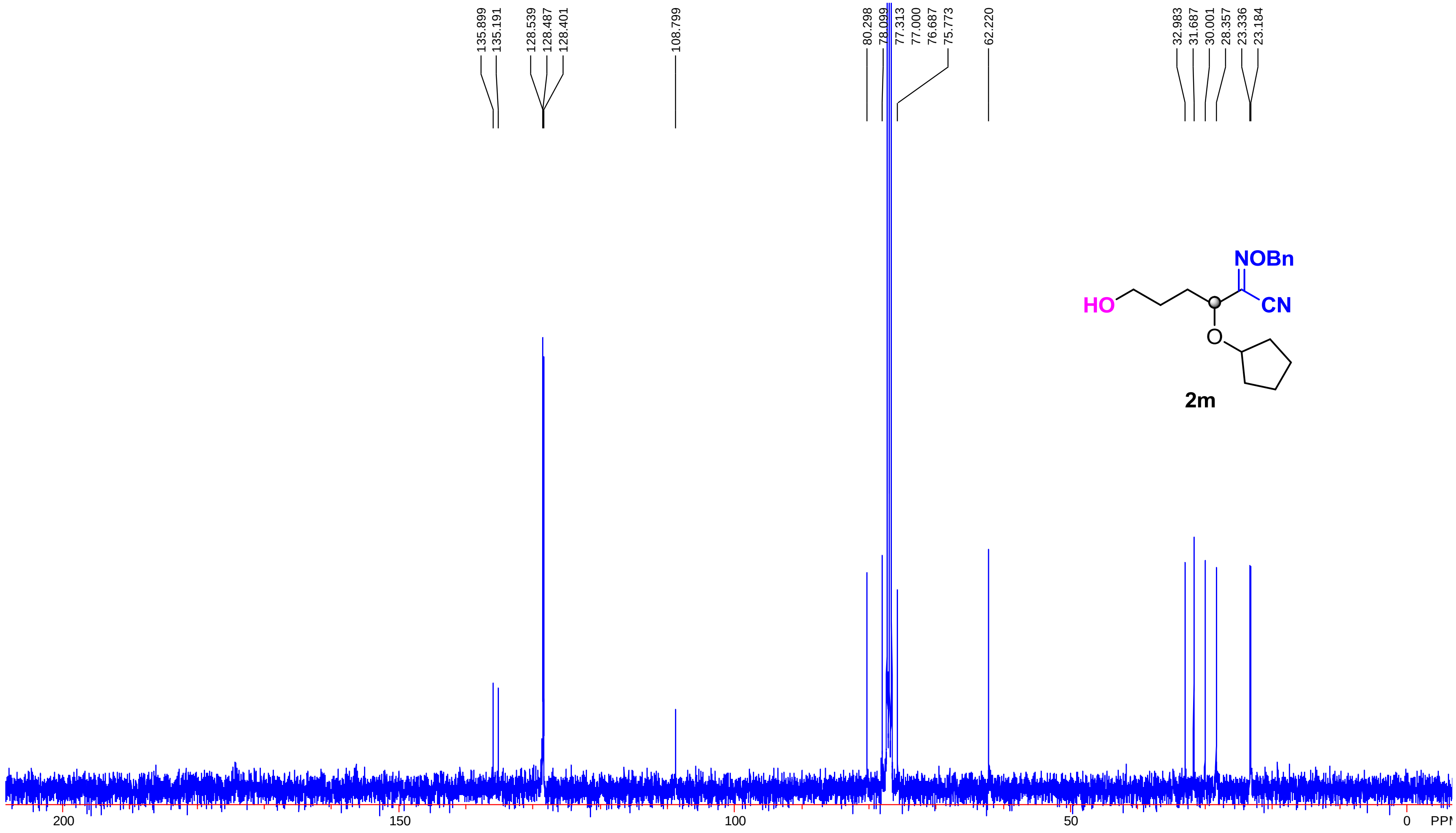

Supplementary Figure 35. ^{13}C NMR of **2m**

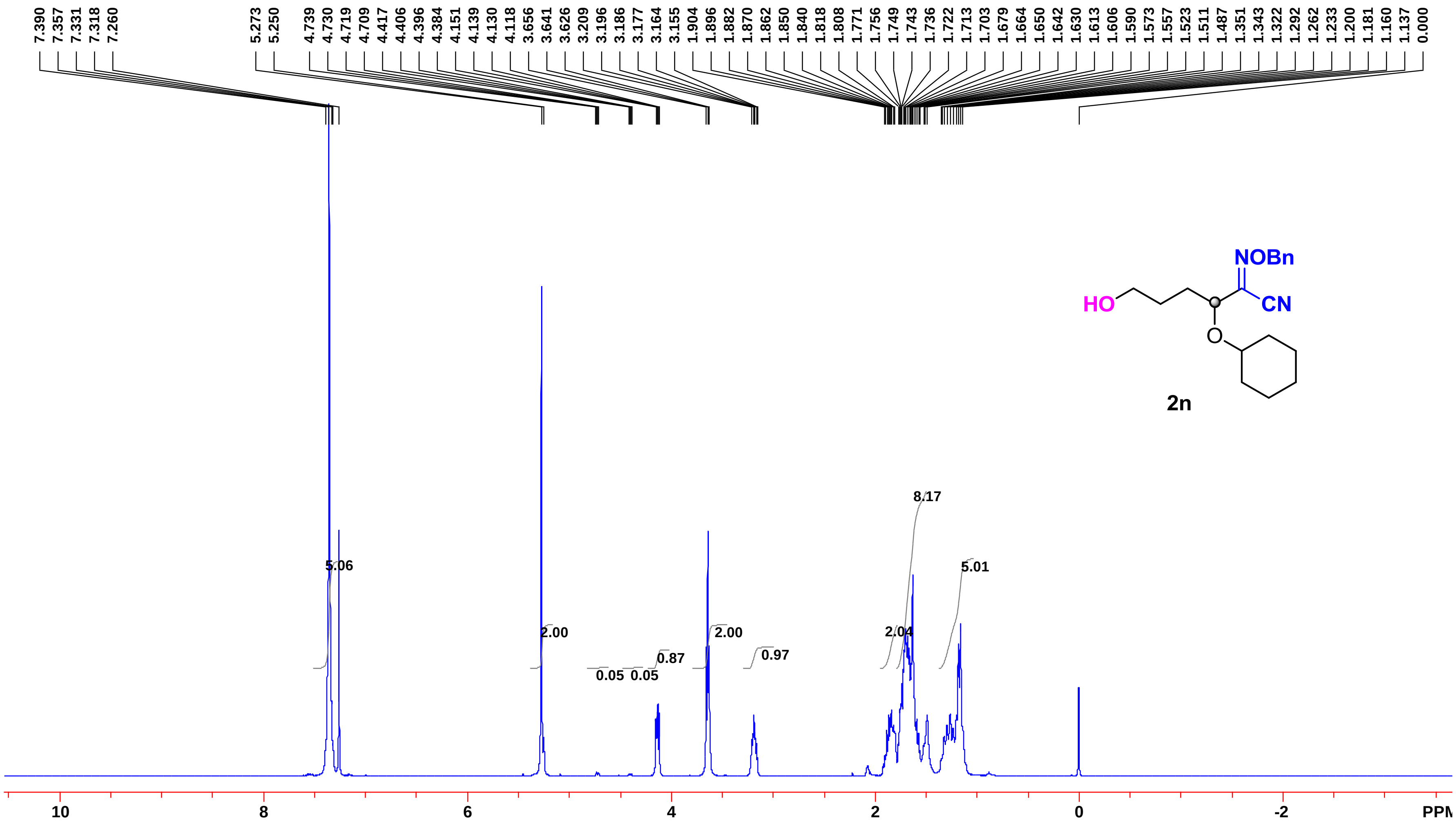

2n

Supplementary Figure 36. ^{1}H NMR of **2n**

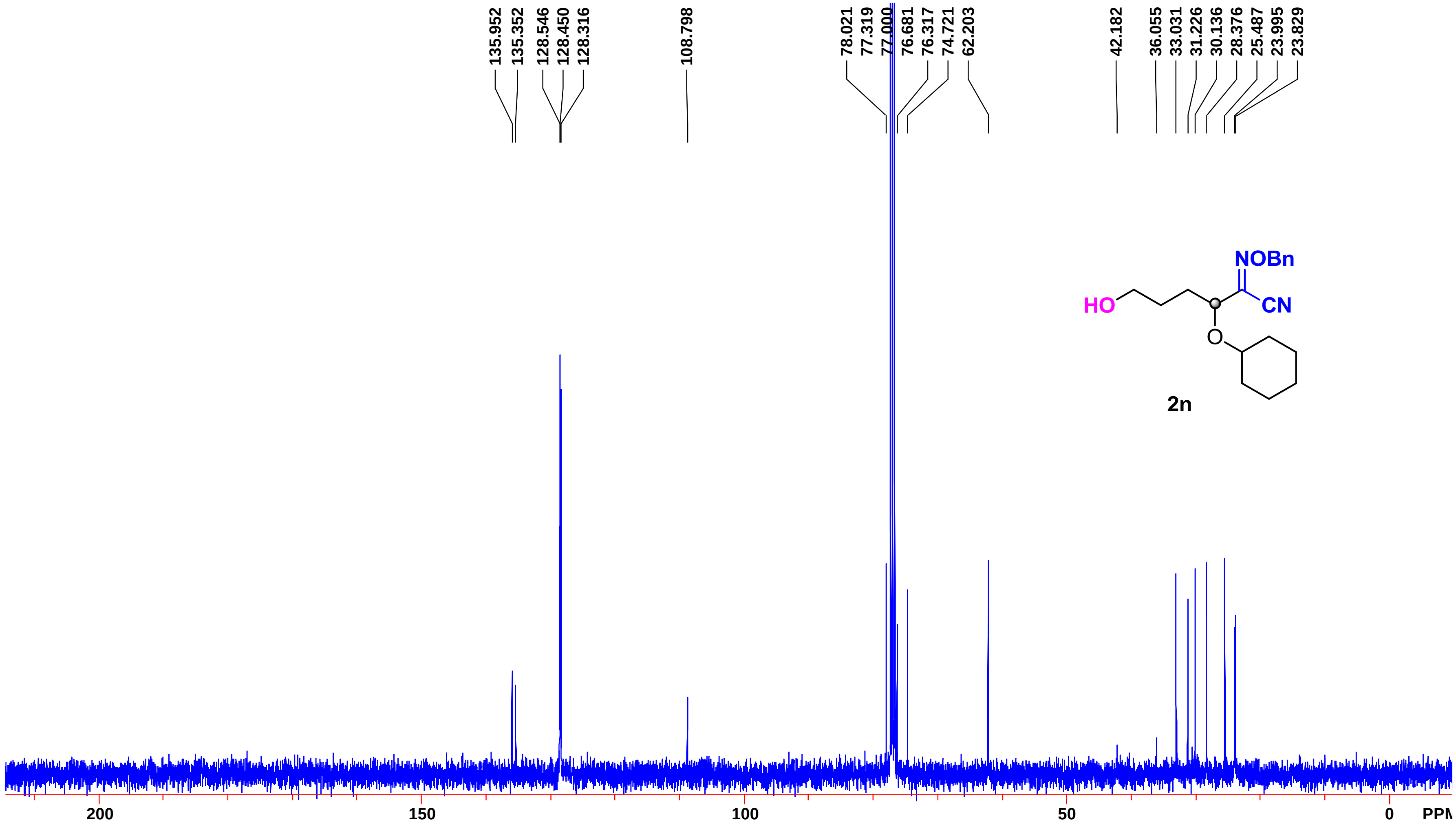

2n

Supplementary Figure 37. ^{13}C NMR of **2n**

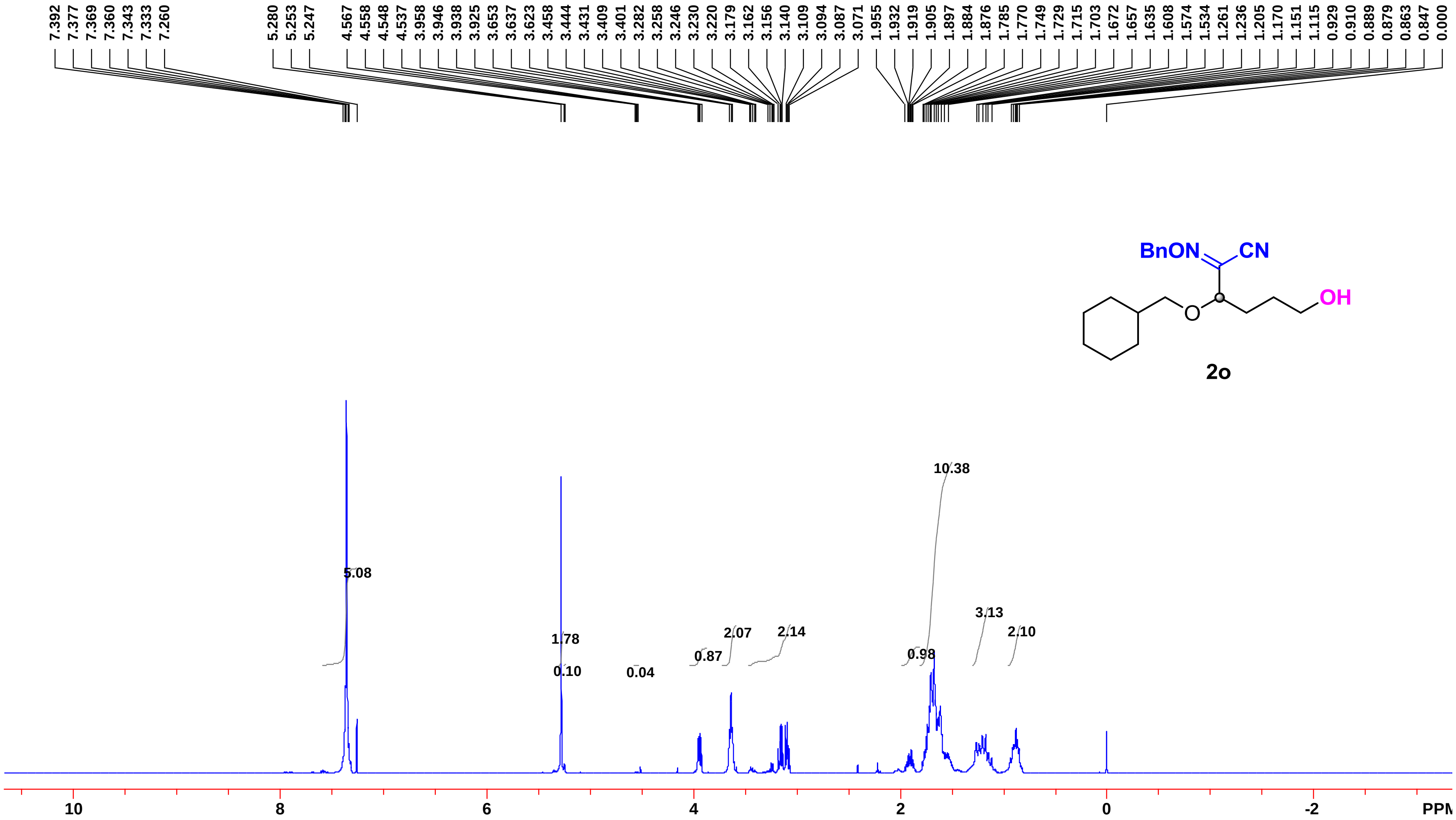

Supplementary Figure 38. ^{1}H NMR of **2o**

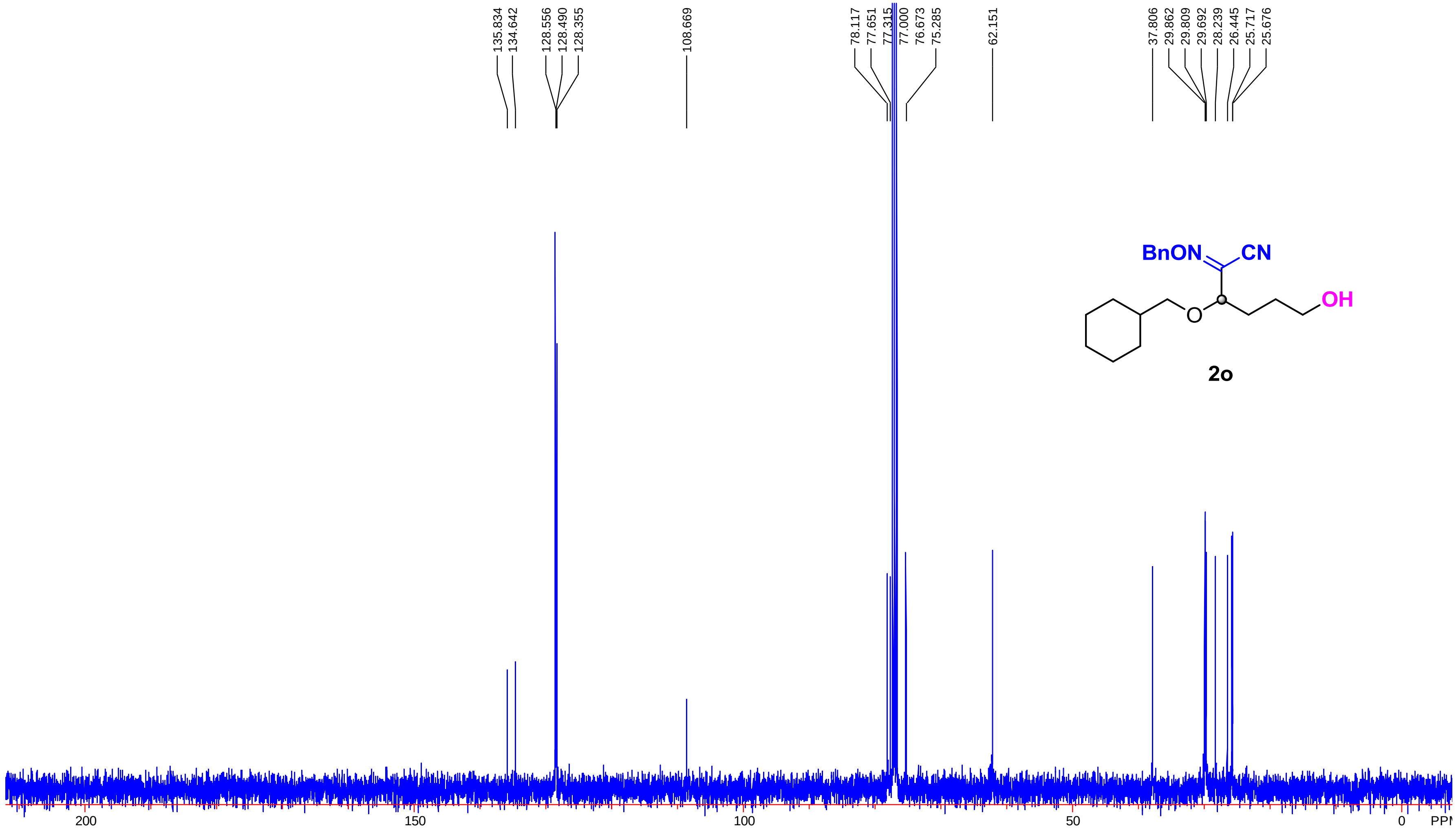

2o

Supplementary Figure 39. ^{13}C NMR of **2o**

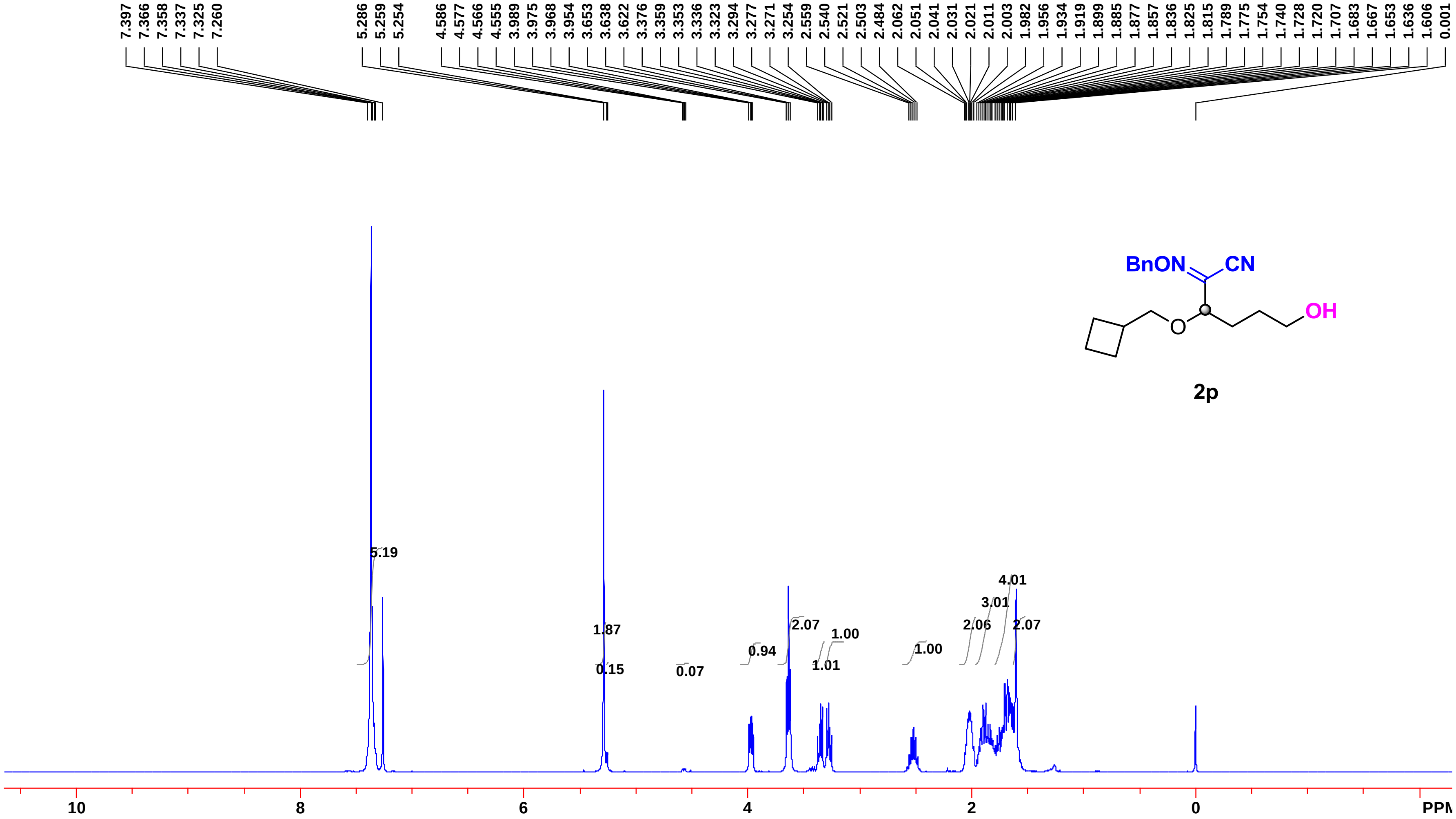

Supplementary Figure 40. ^{1}H NMR of **2p**

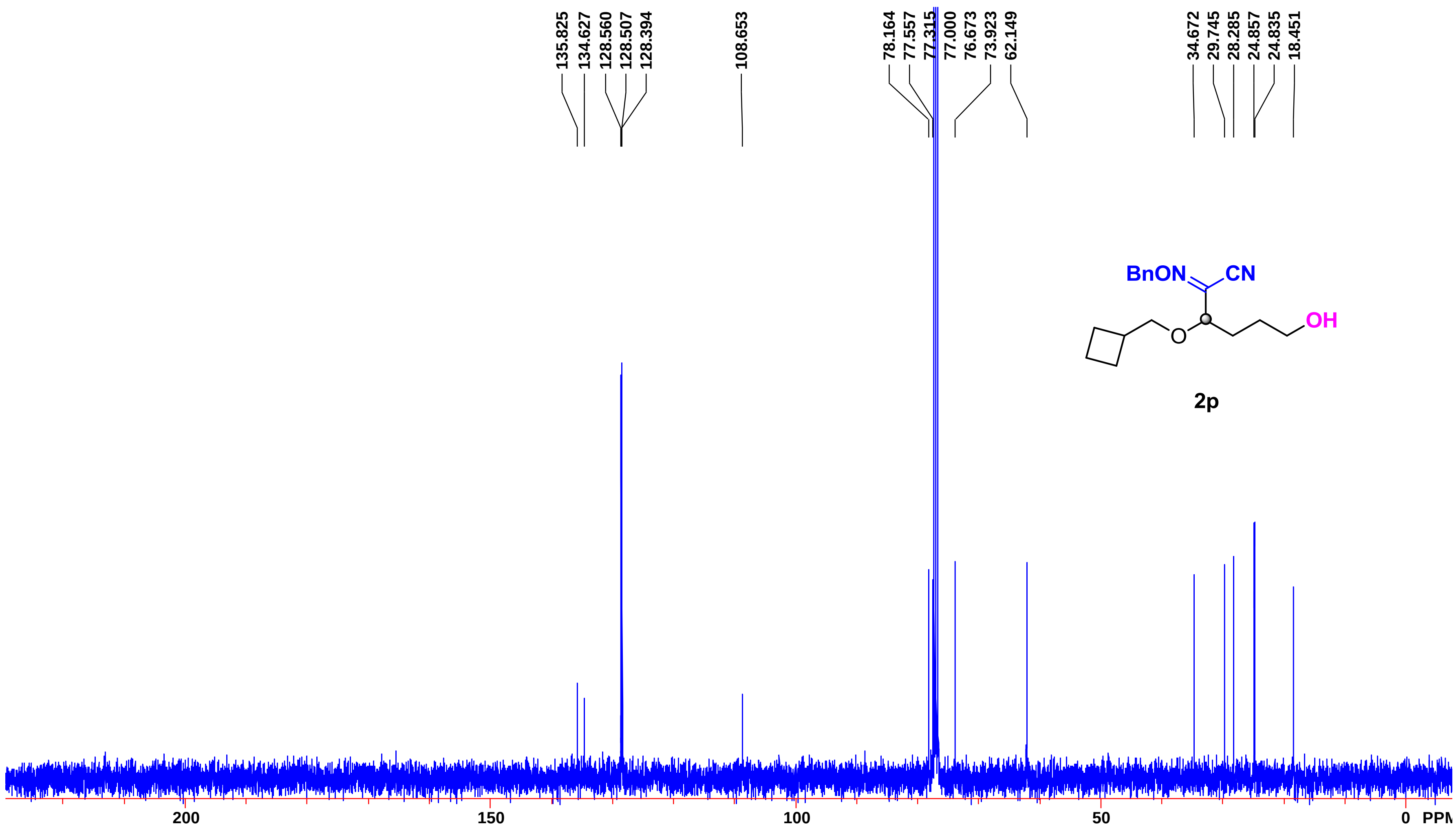

Supplementary Figure 41. ^{13}C NMR of **2p**

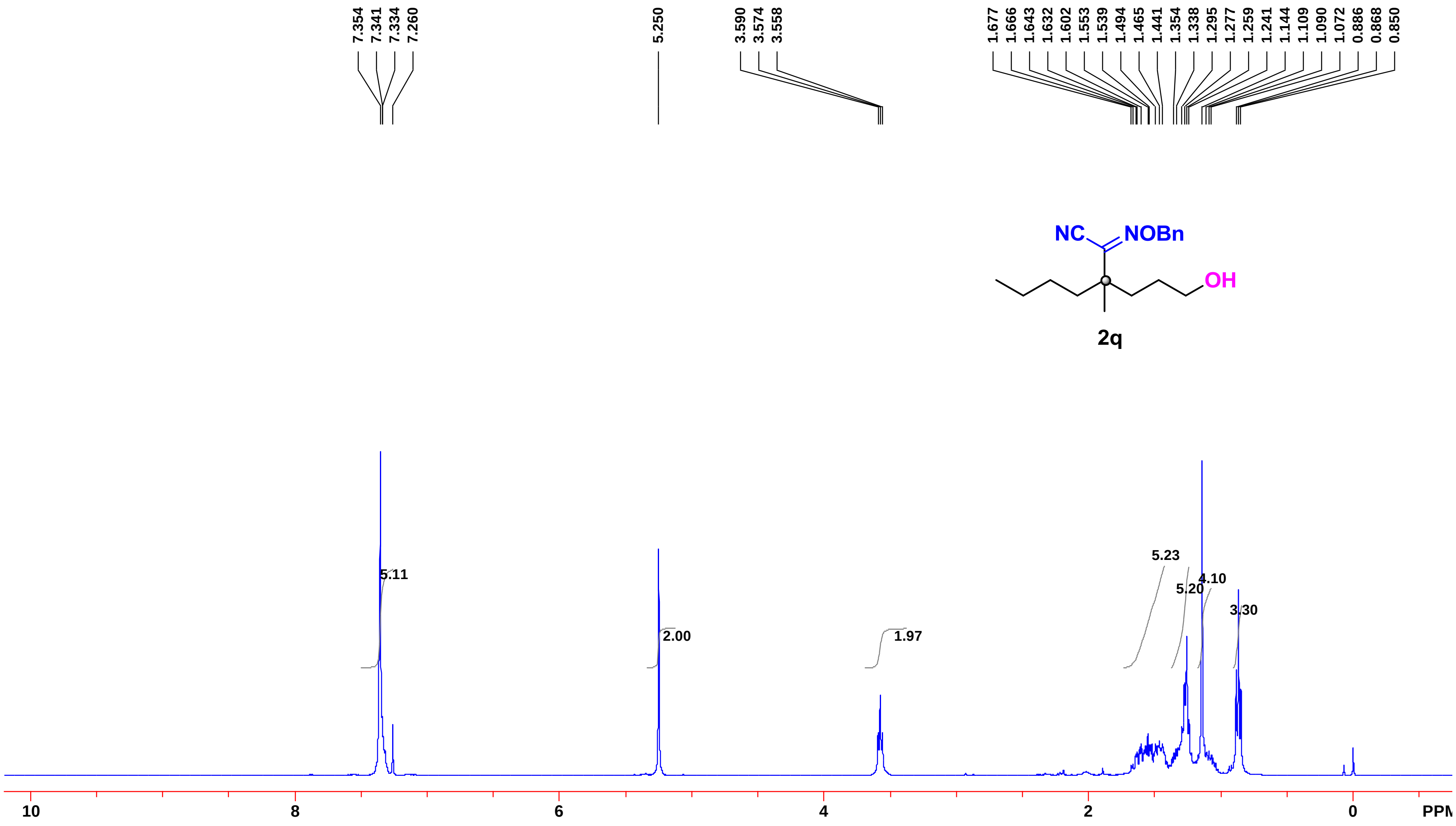

Supplementary Figure 42. ^{1}H NMR of **2q**

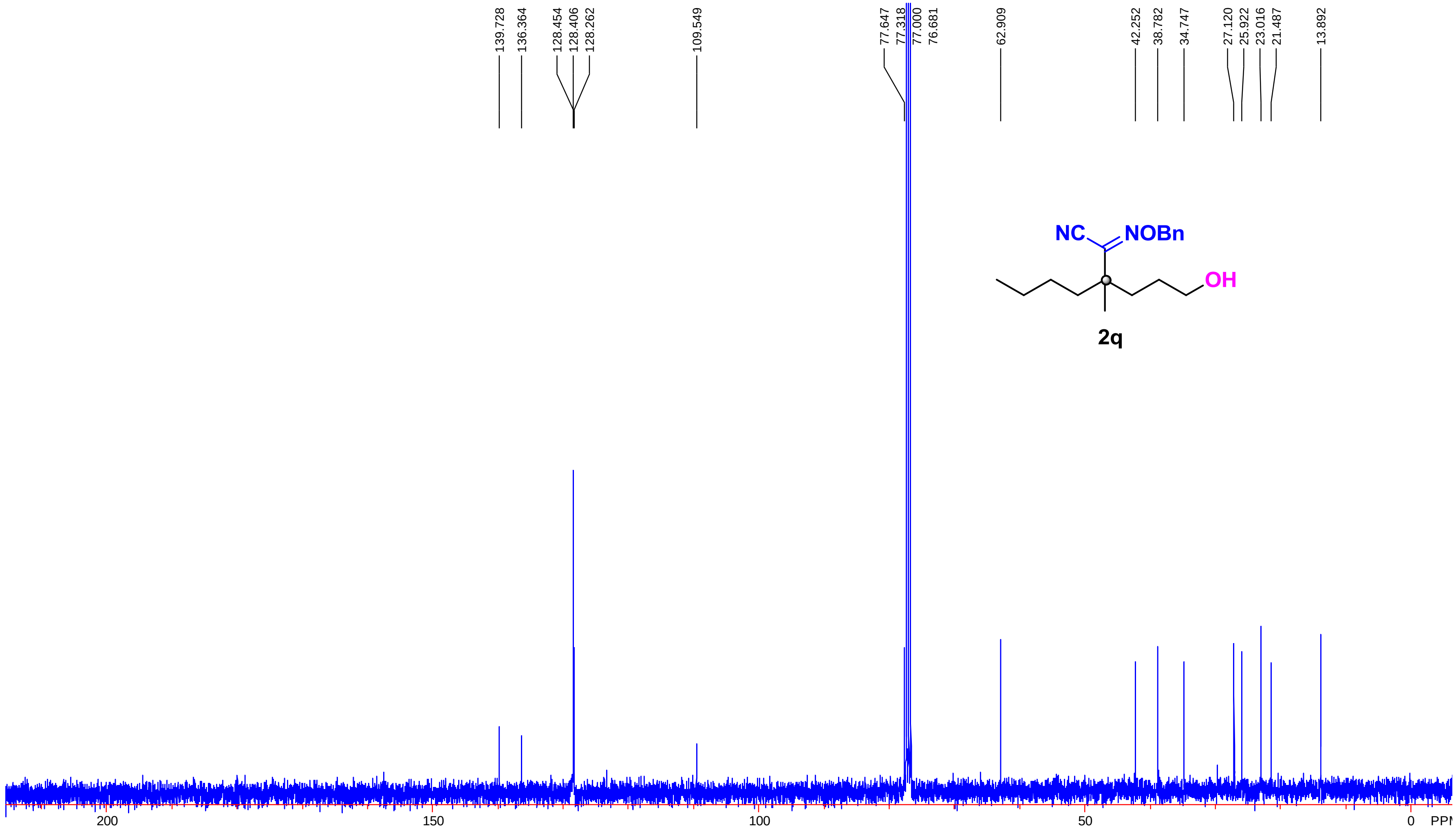

Supplementary Figure 43. ^{13}C NMR of **2q**

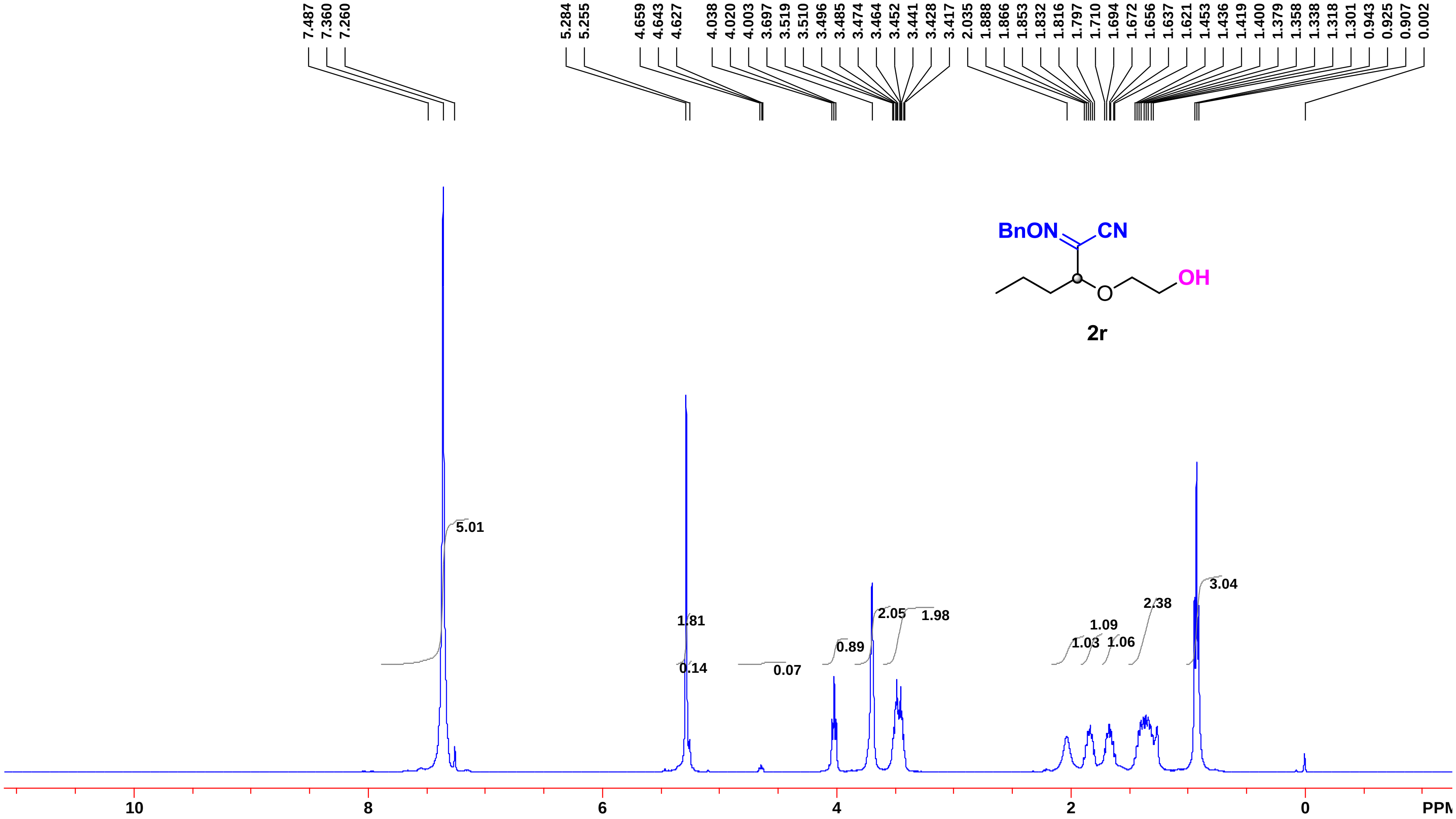

Supplementary Figure 44. ^{1}H NMR of **2r**

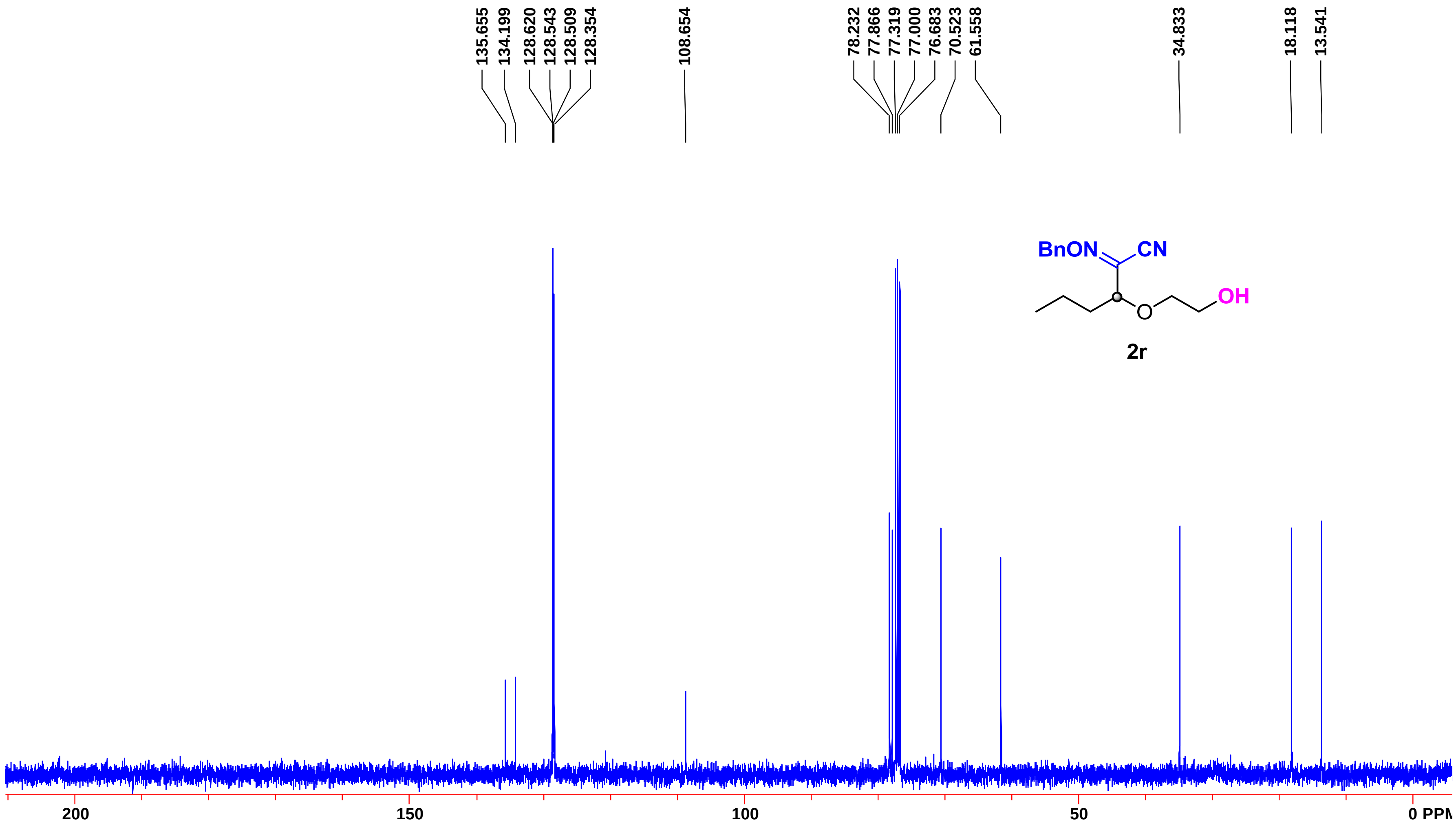

Supplementary Figure 45. ^{13}C NMR of **2r**

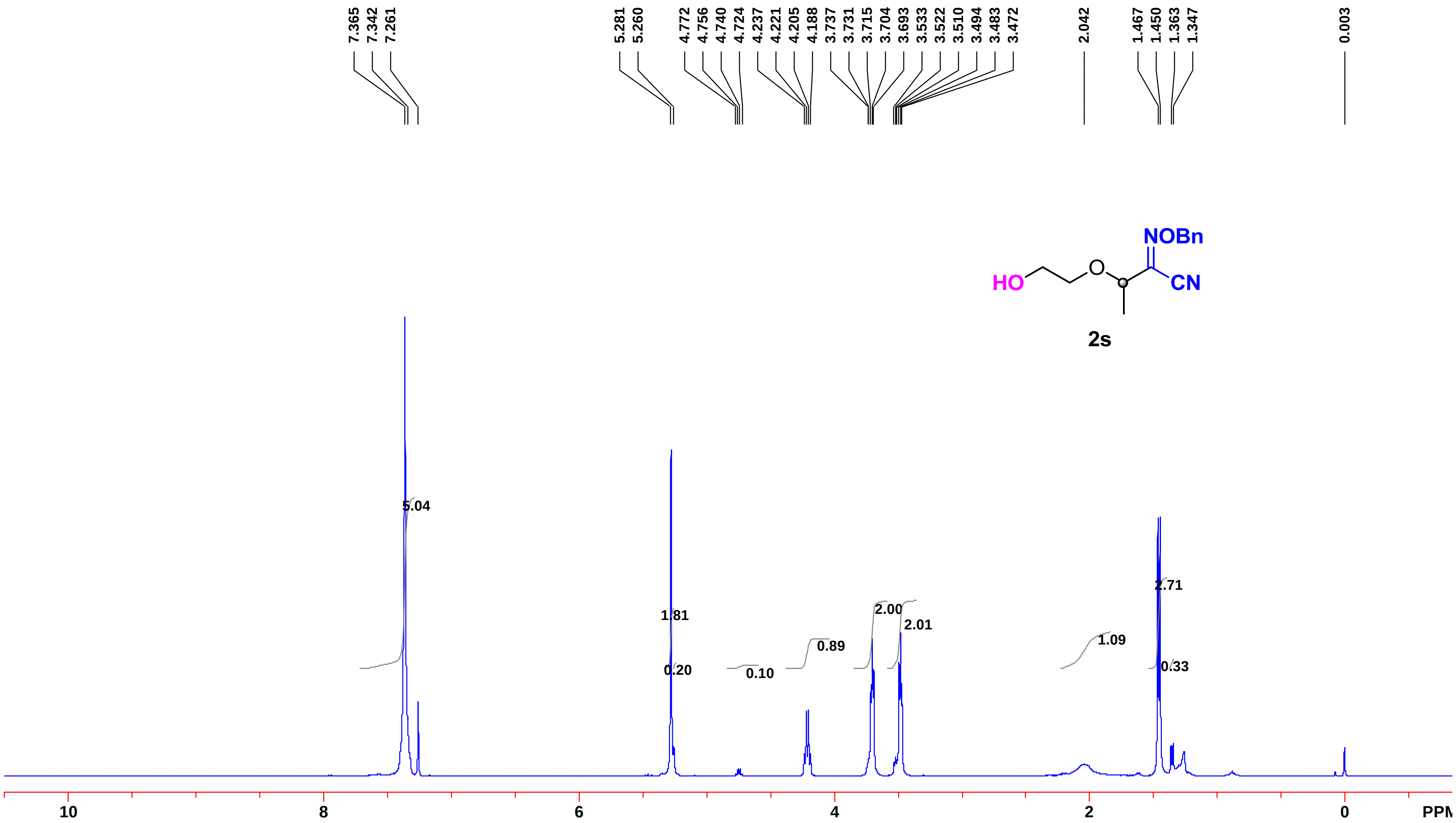

Supplementary Figure 46. ^{1}H NMR of **2s**

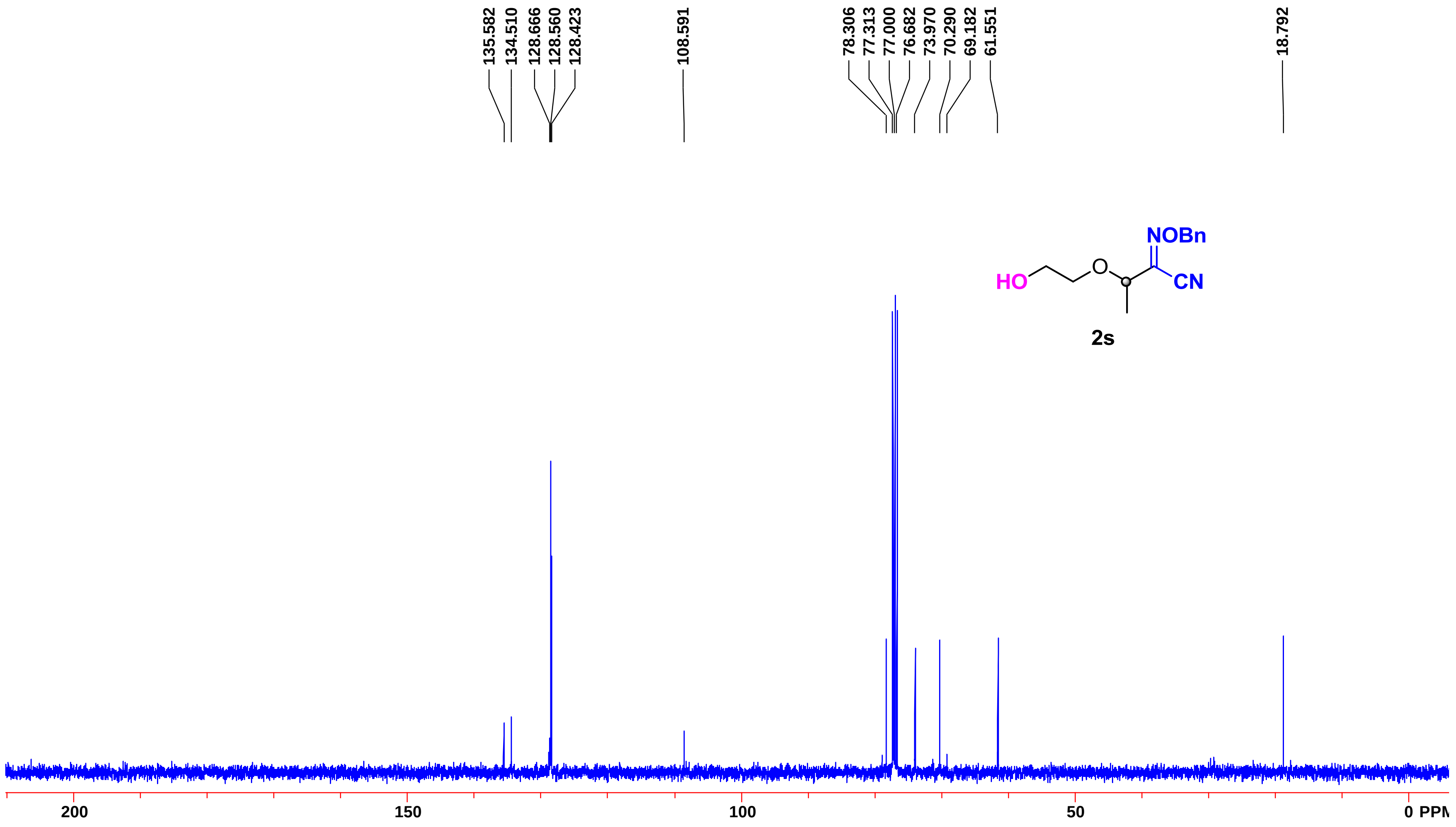

Supplementary Figure 47. ^{13}C NMR of **2s**

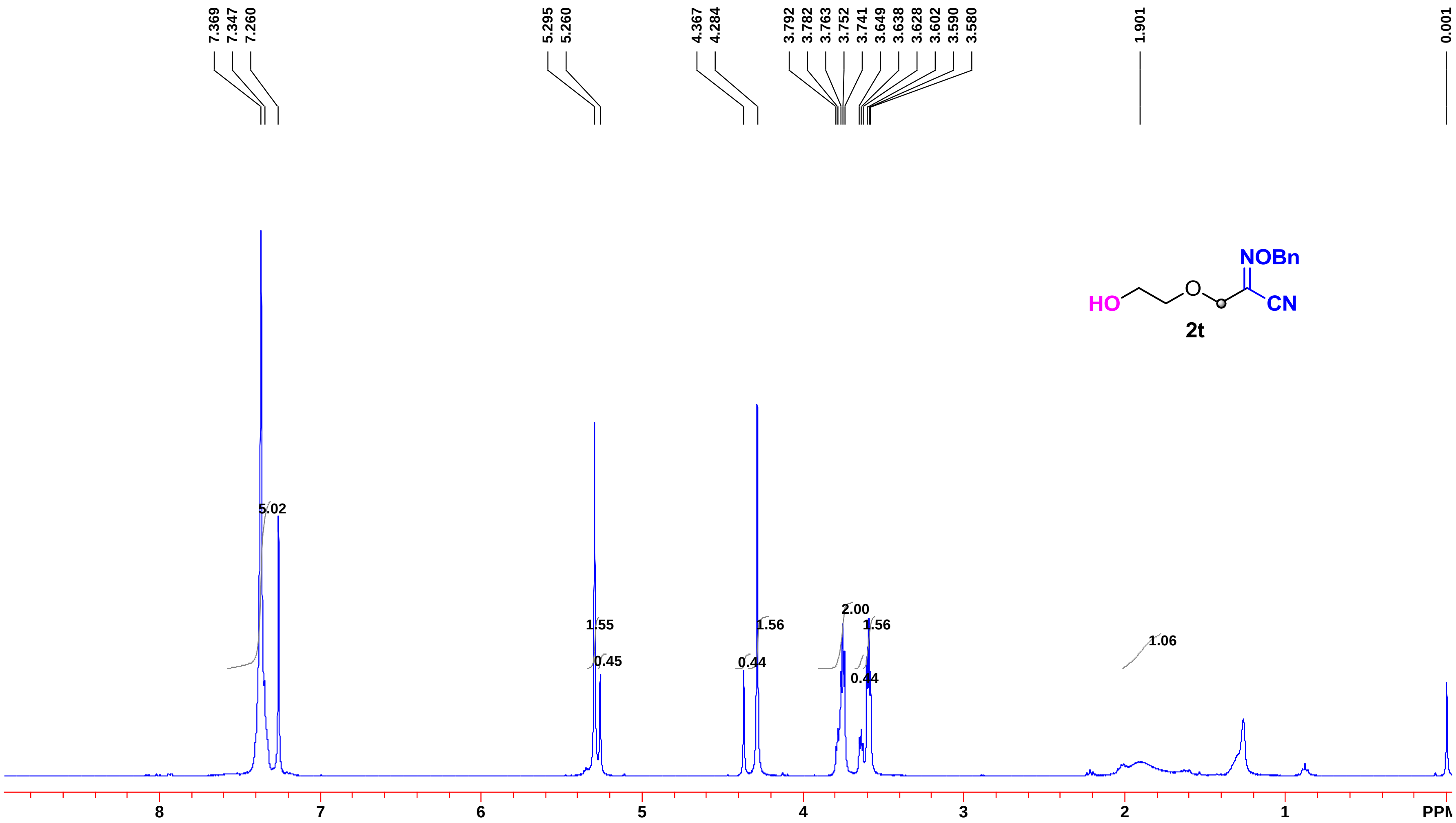

Supplementary Figure 48. ^{1}H NMR of **2t**

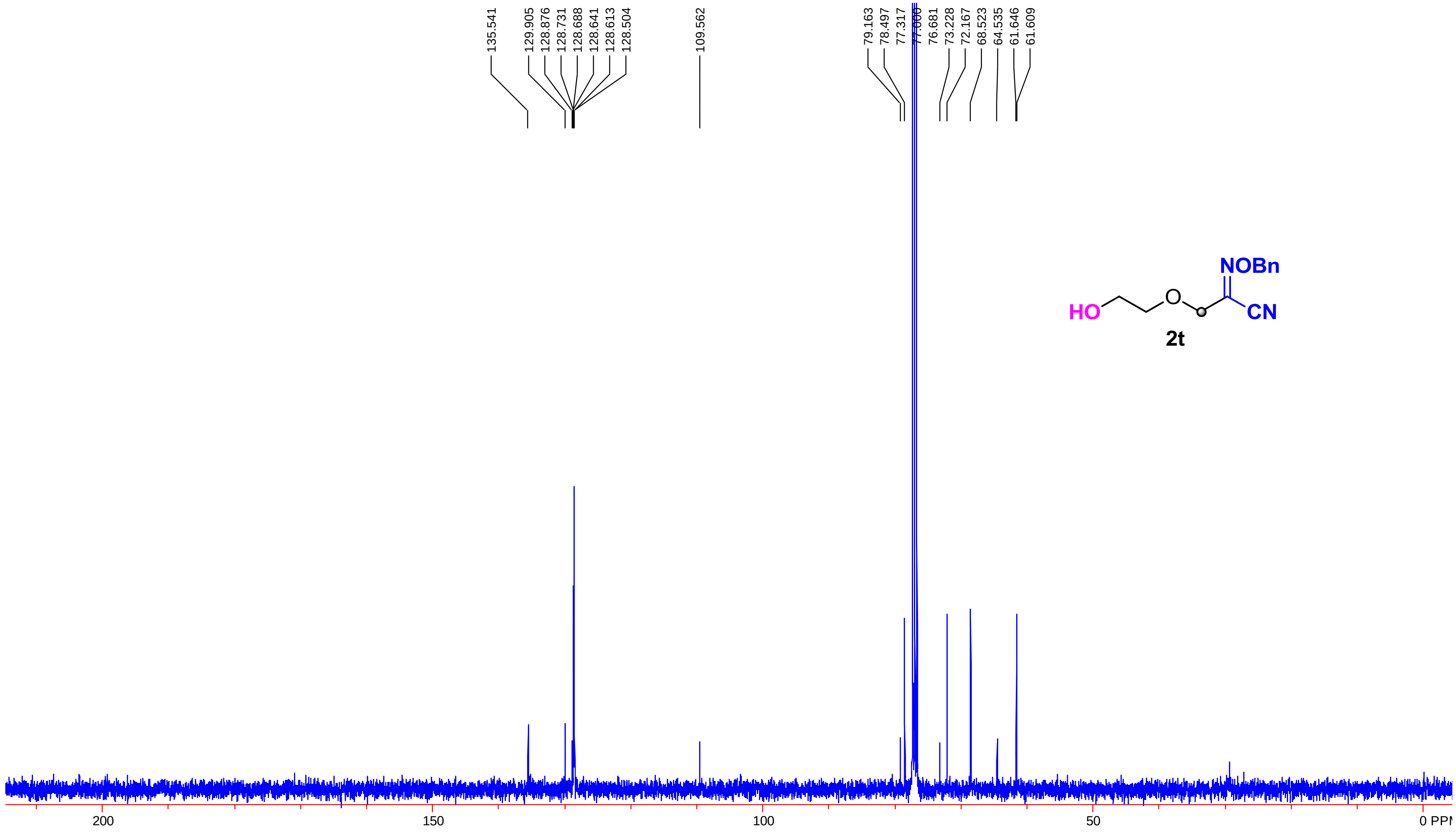

Supplementary Figure 49. ^{13}C NMR of **2t**

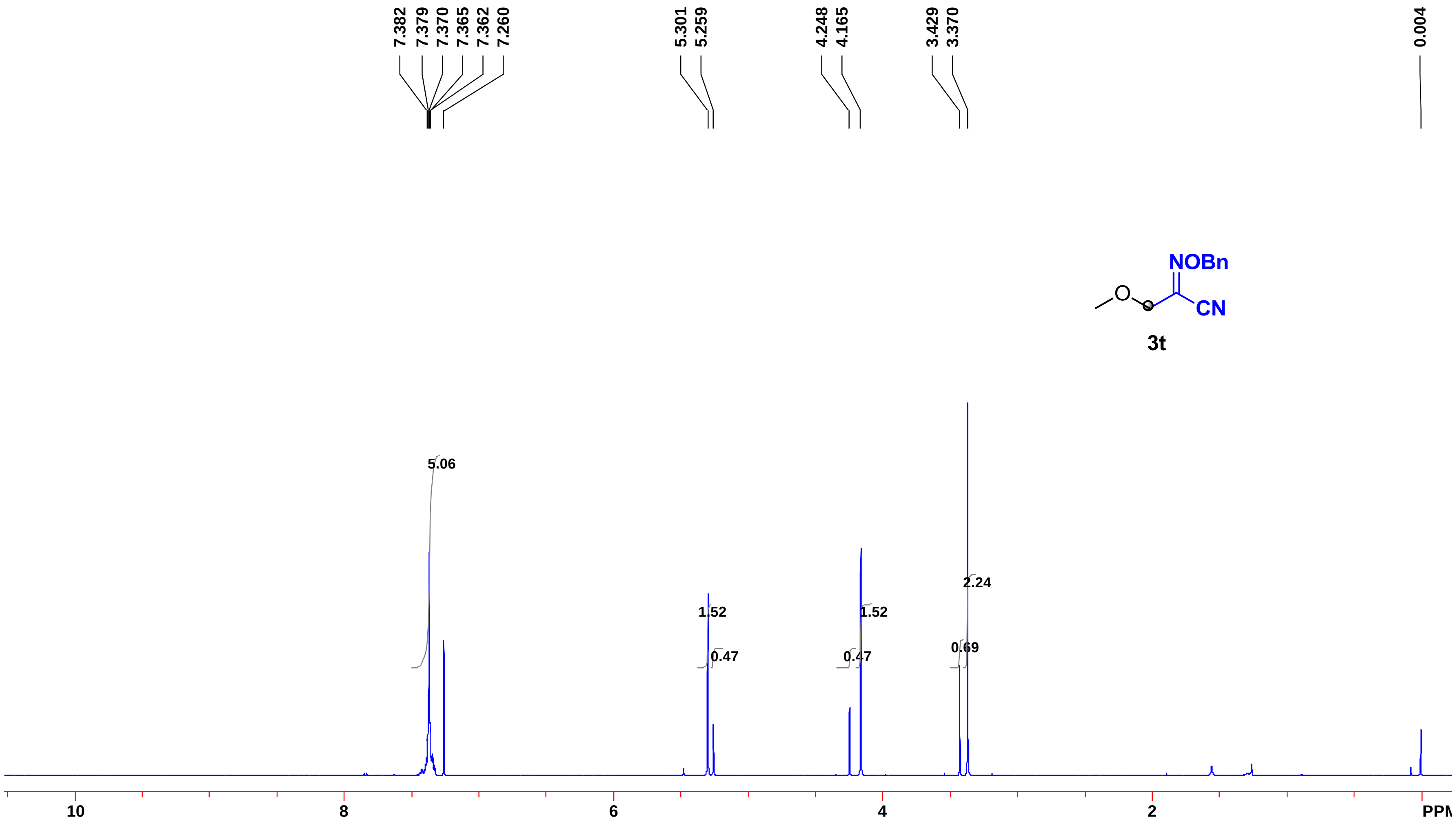

Supplementary Figure 50. ^{1}H NMR of **3t**

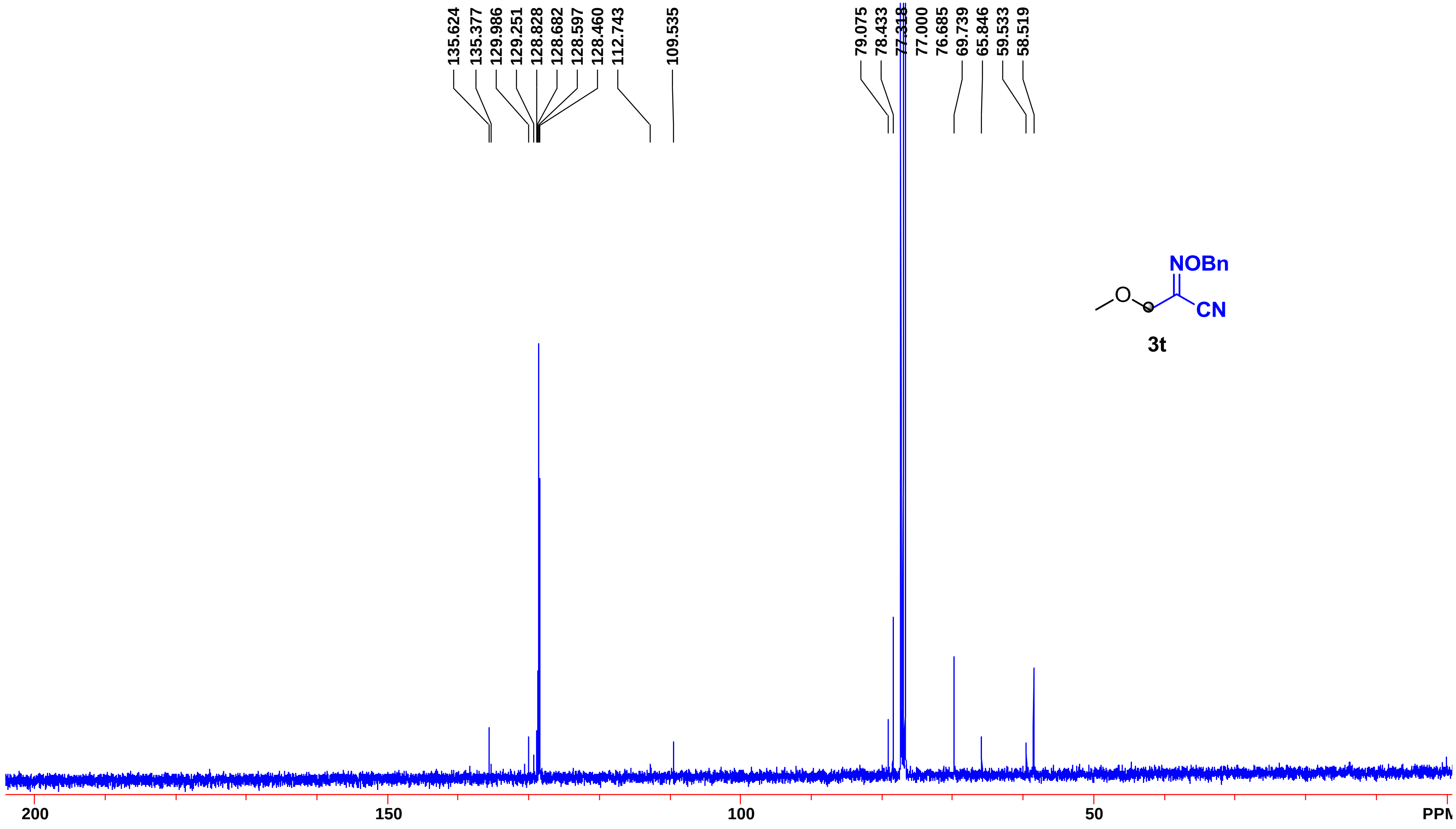

Supplementary Figure 51. ^{13}C NMR of **3t**

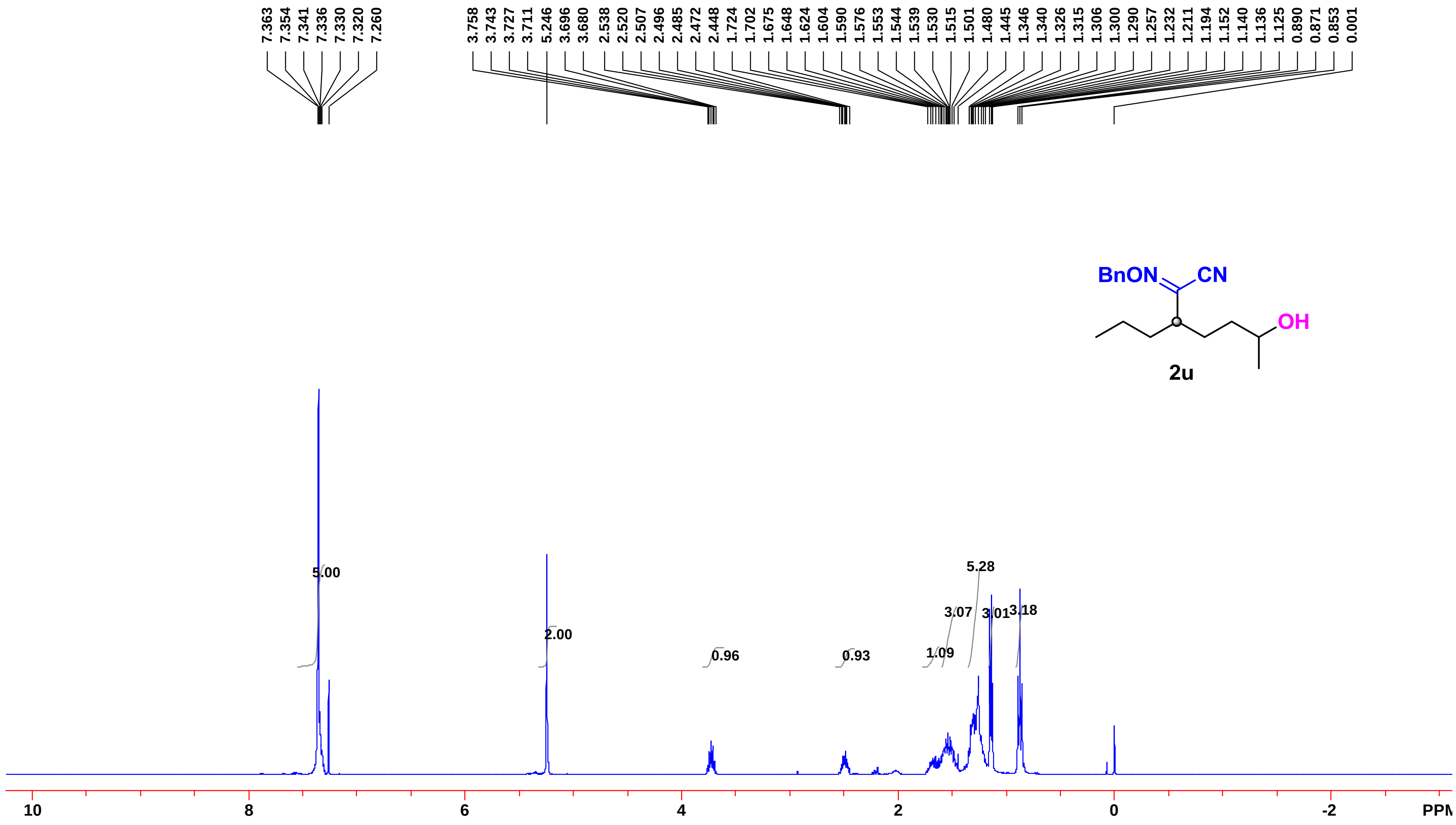

Supplementary Figure 52. ^{1}H NMR of **2u**

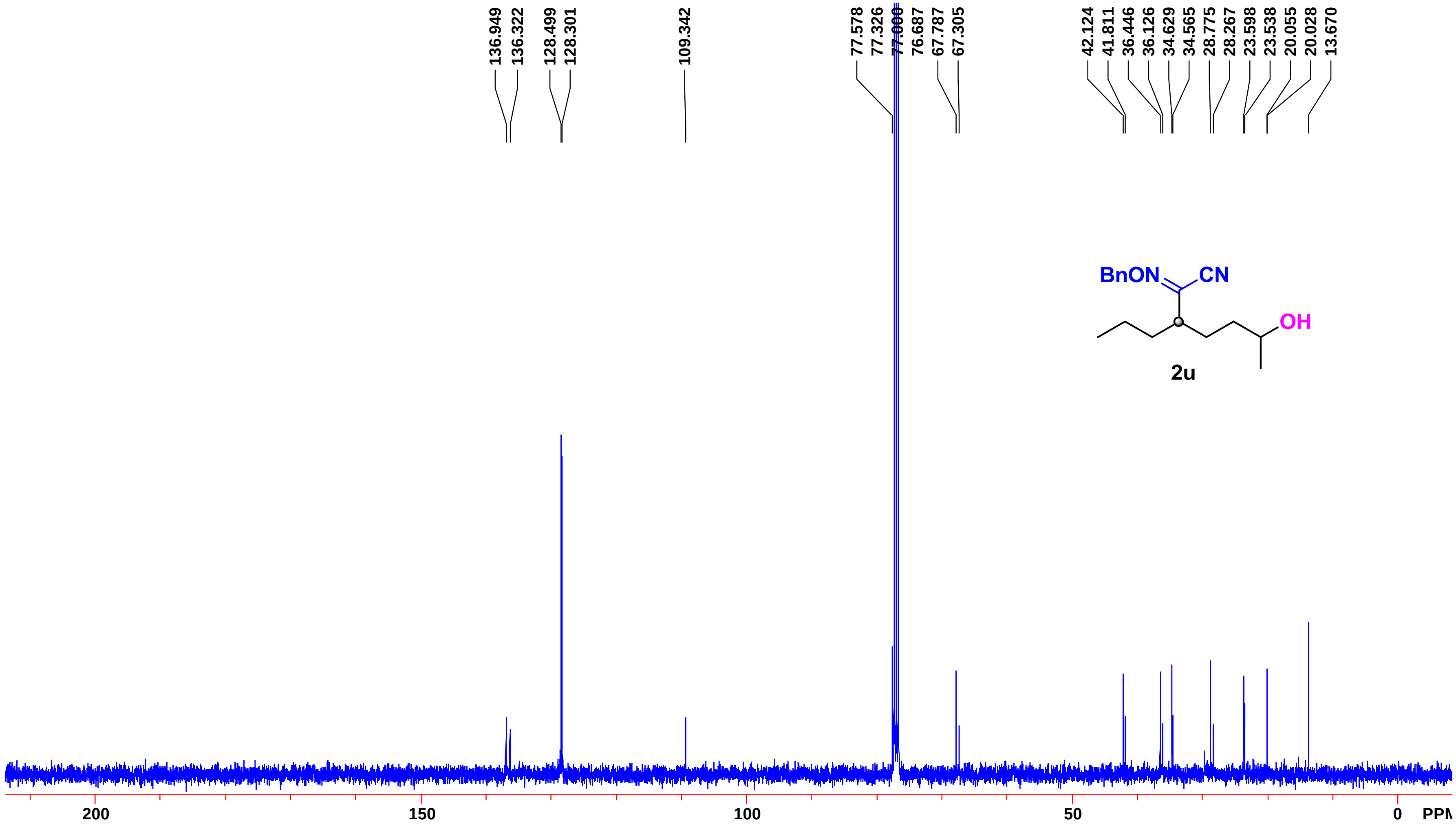

Supplementary Figure 53. ^{13}C NMR of **2u**

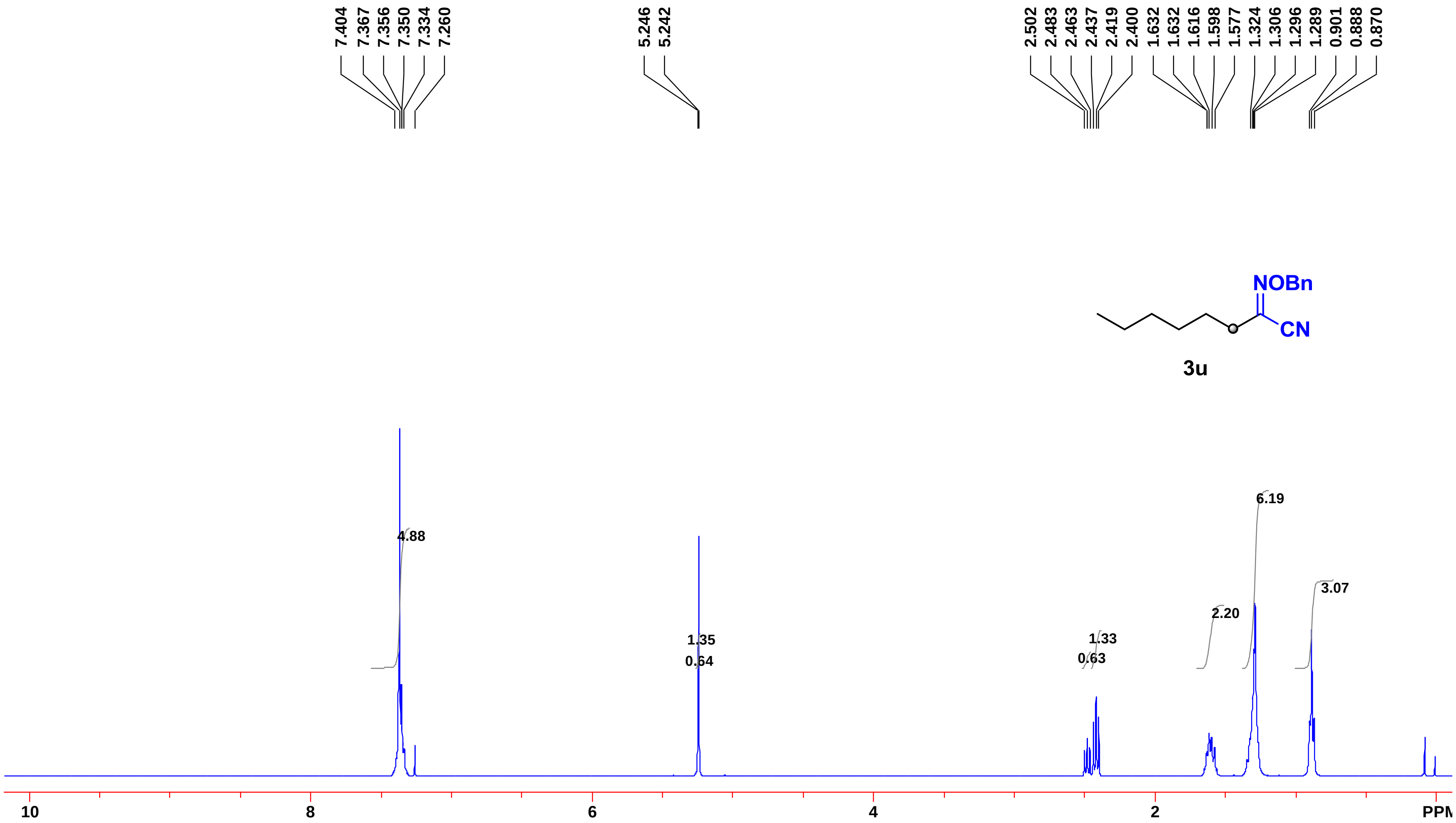

Supplementary Figure 54. ^{1}H NMR of **3u**

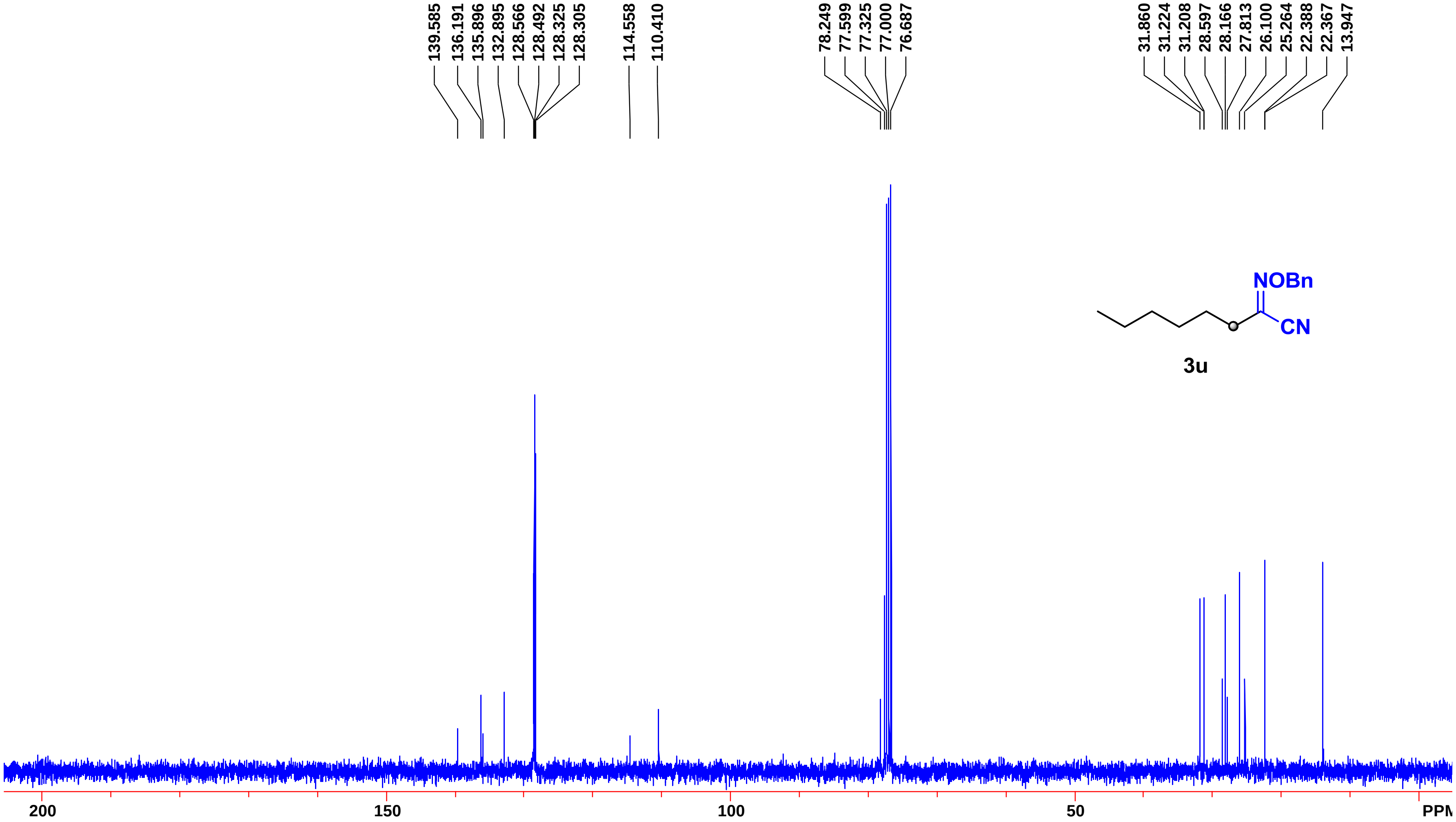

Supplementary Figure 55. ^{13}C NMR of **3u**

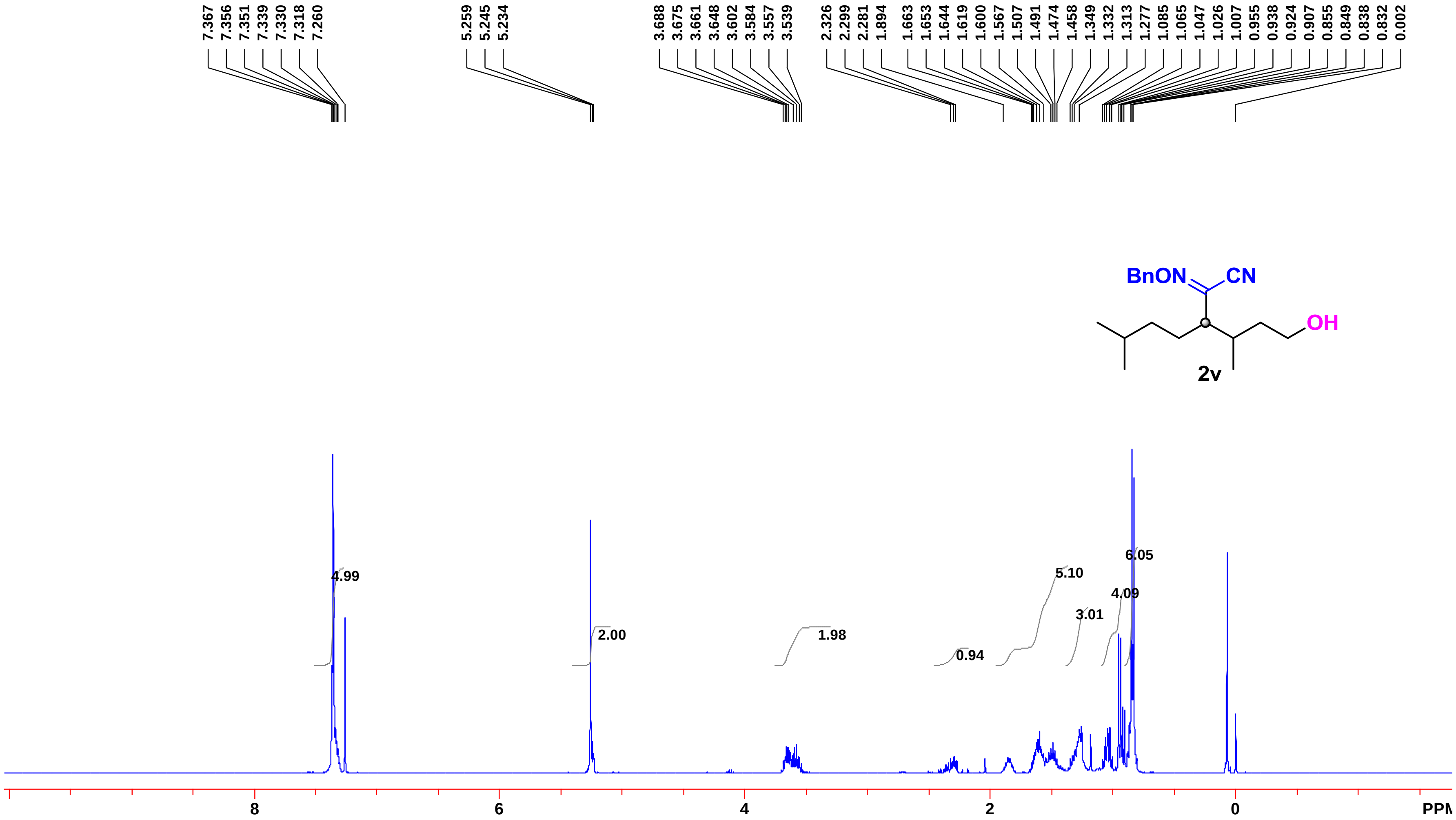

Supplementary Figure 56. ^{1}H NMR of **2v**

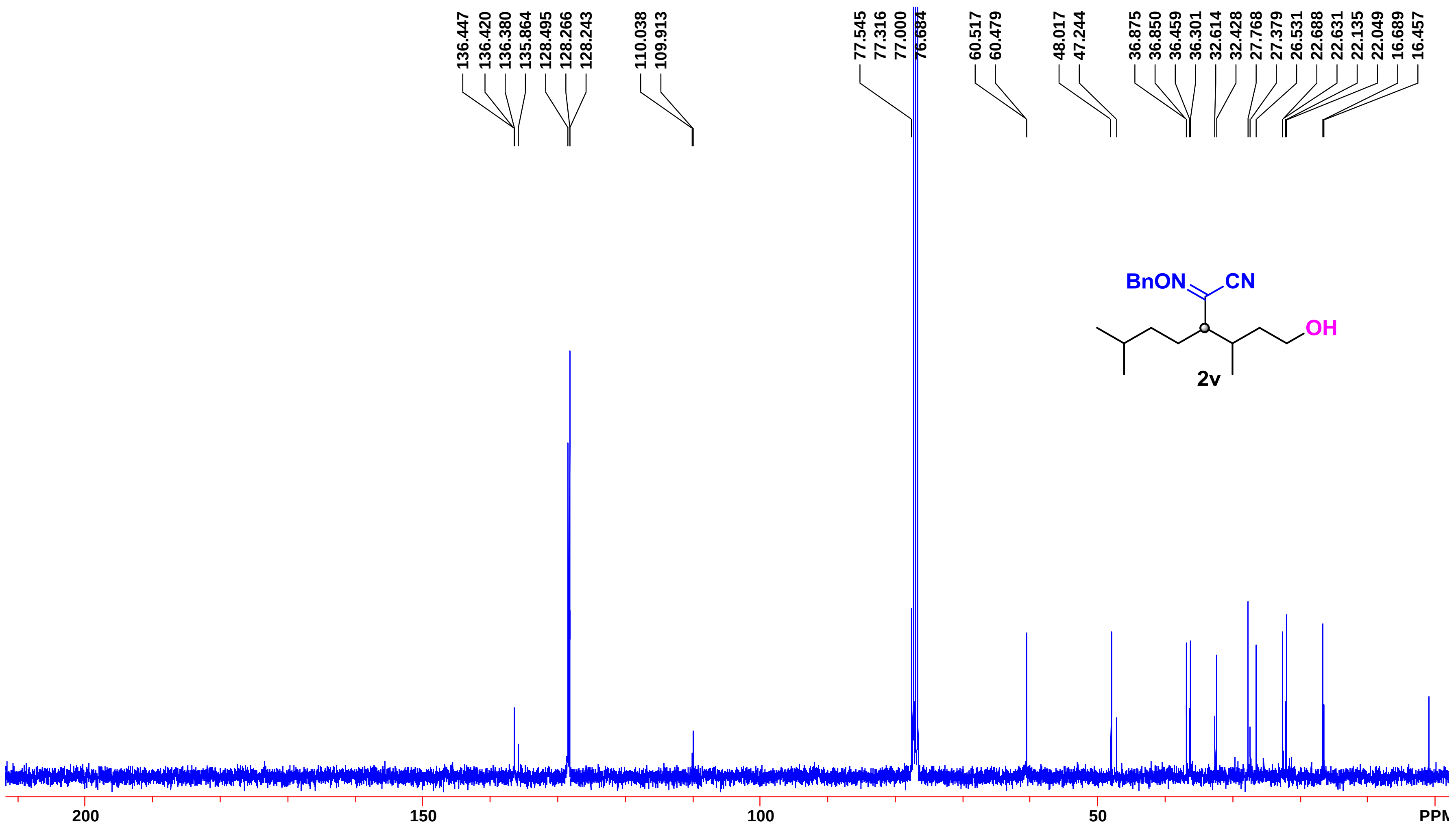

Supplementary Figure 57. ^{13}C NMR of **2v**

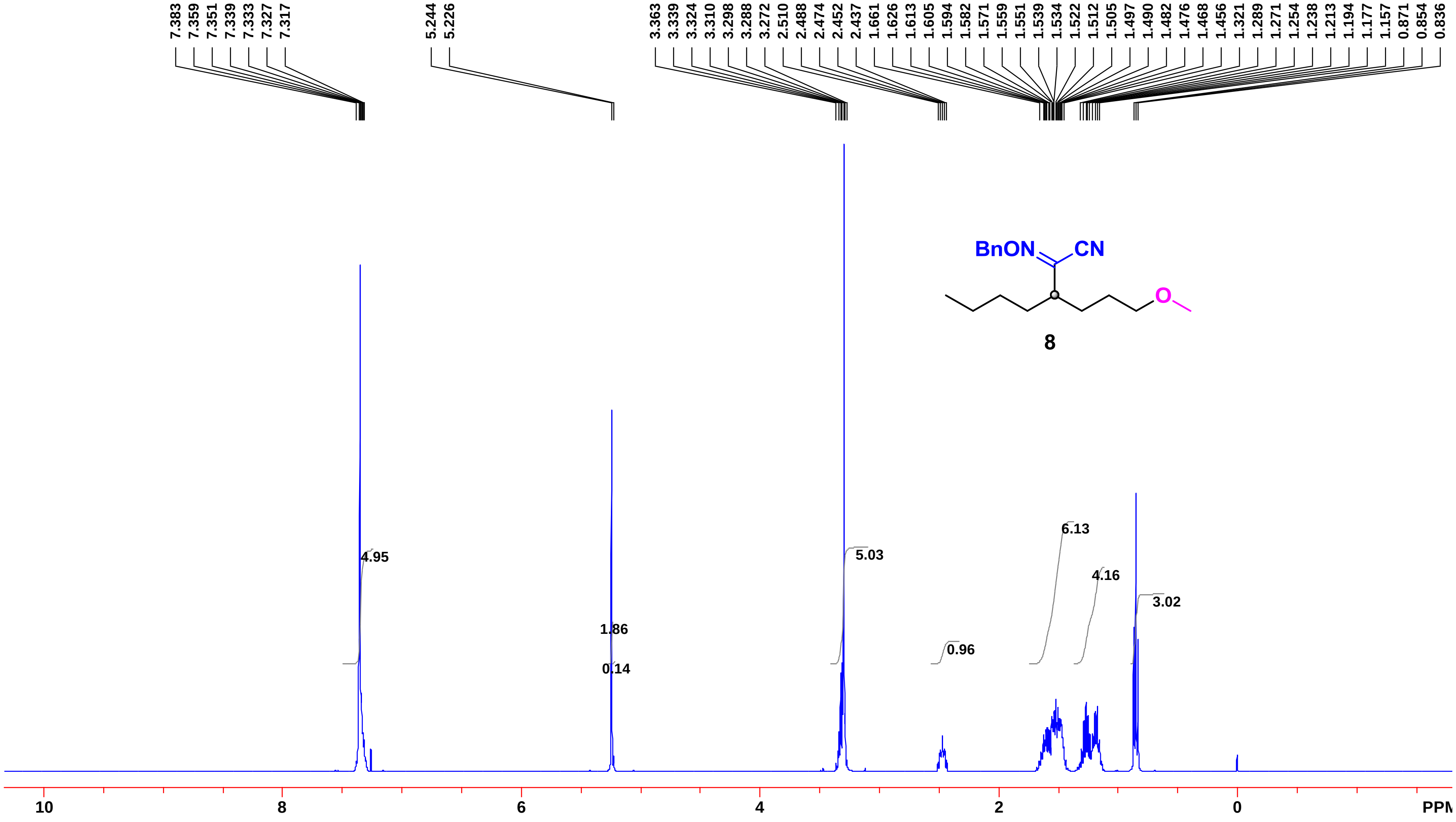

Supplementary Figure 58. ^{1}H NMR of **8**

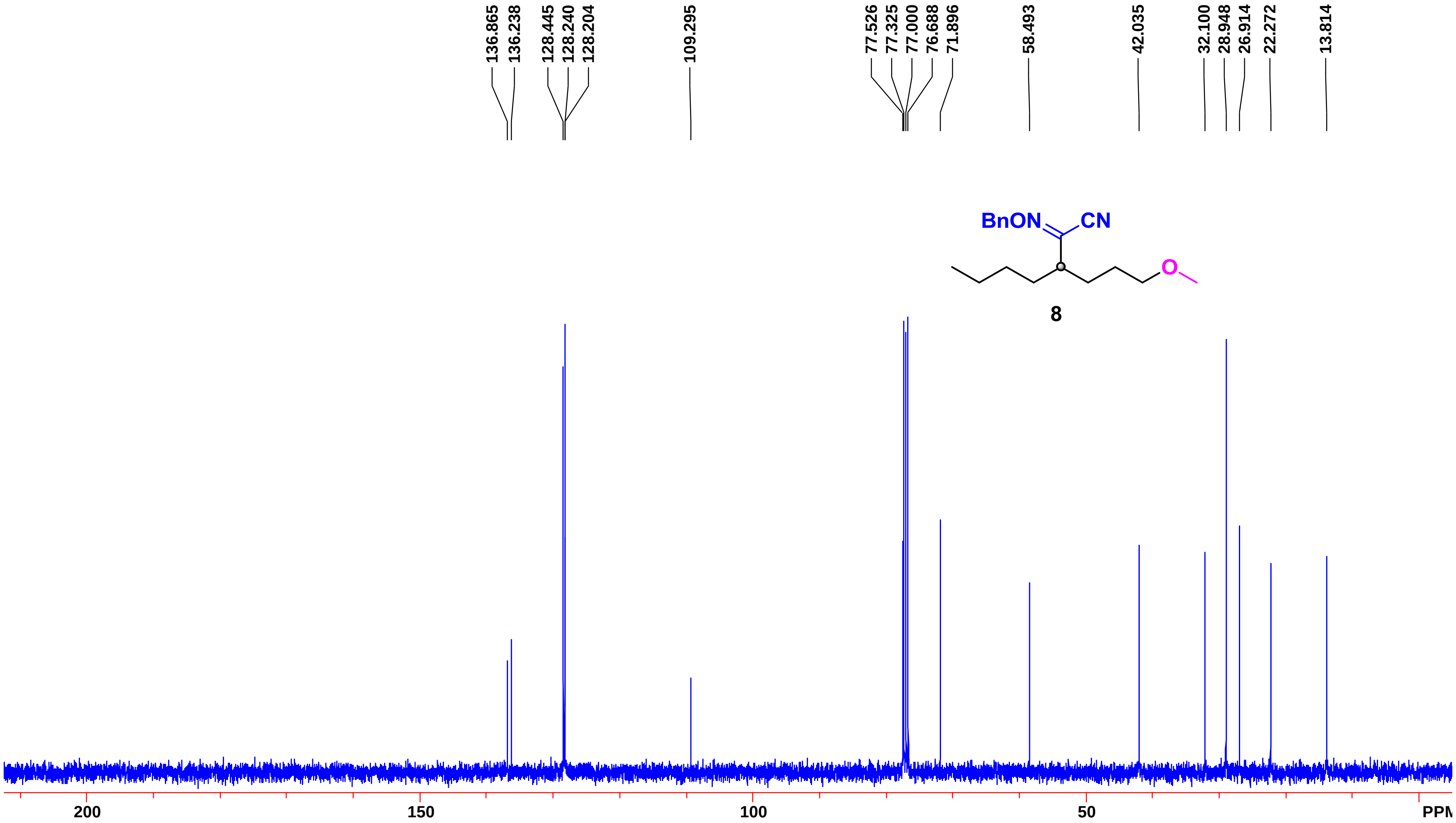

Supplementary Figure 59. ^{13}C NMR of **8**

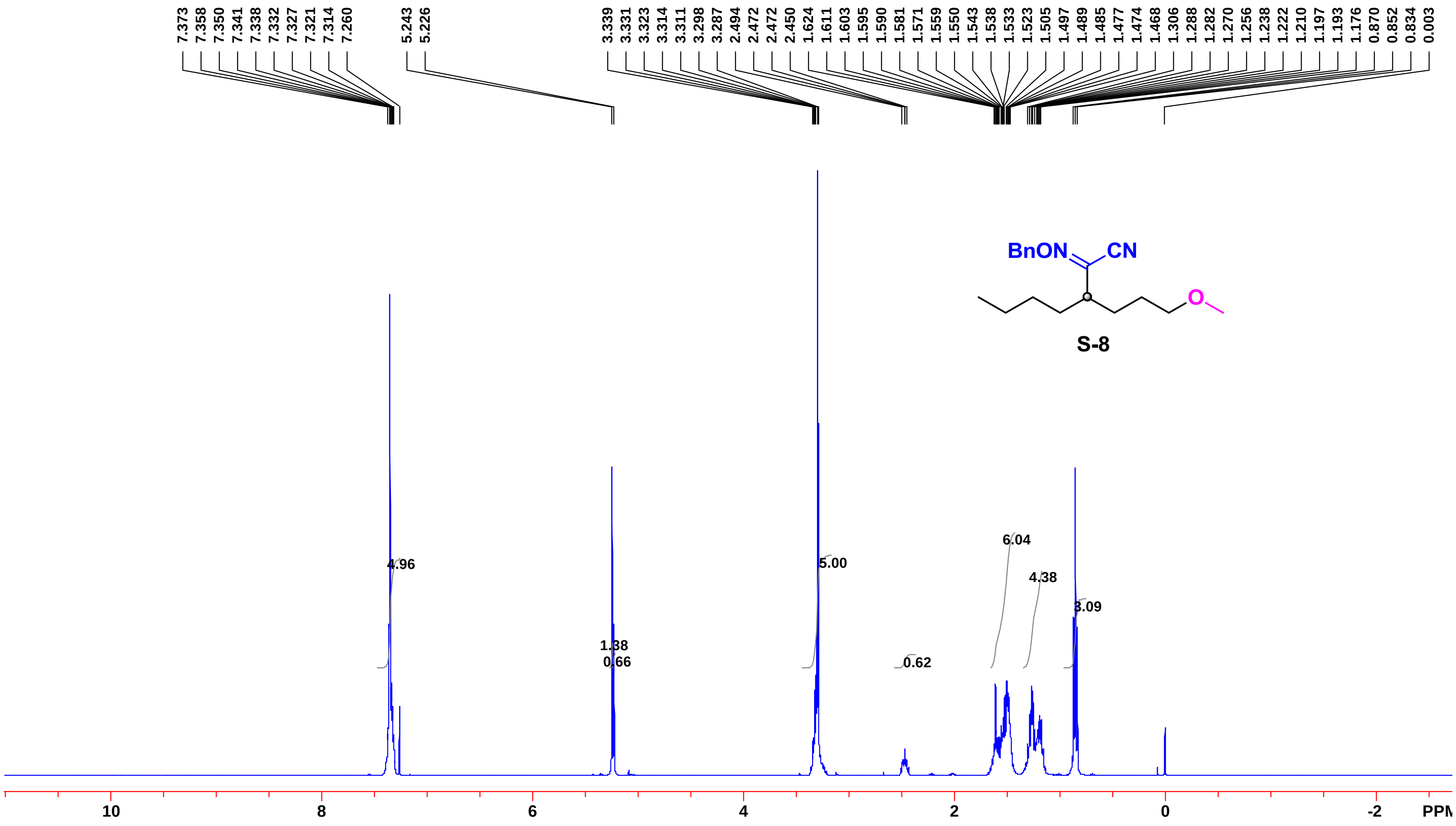

Supplementary Figure 60. ^{1}H NMR of **S-8**

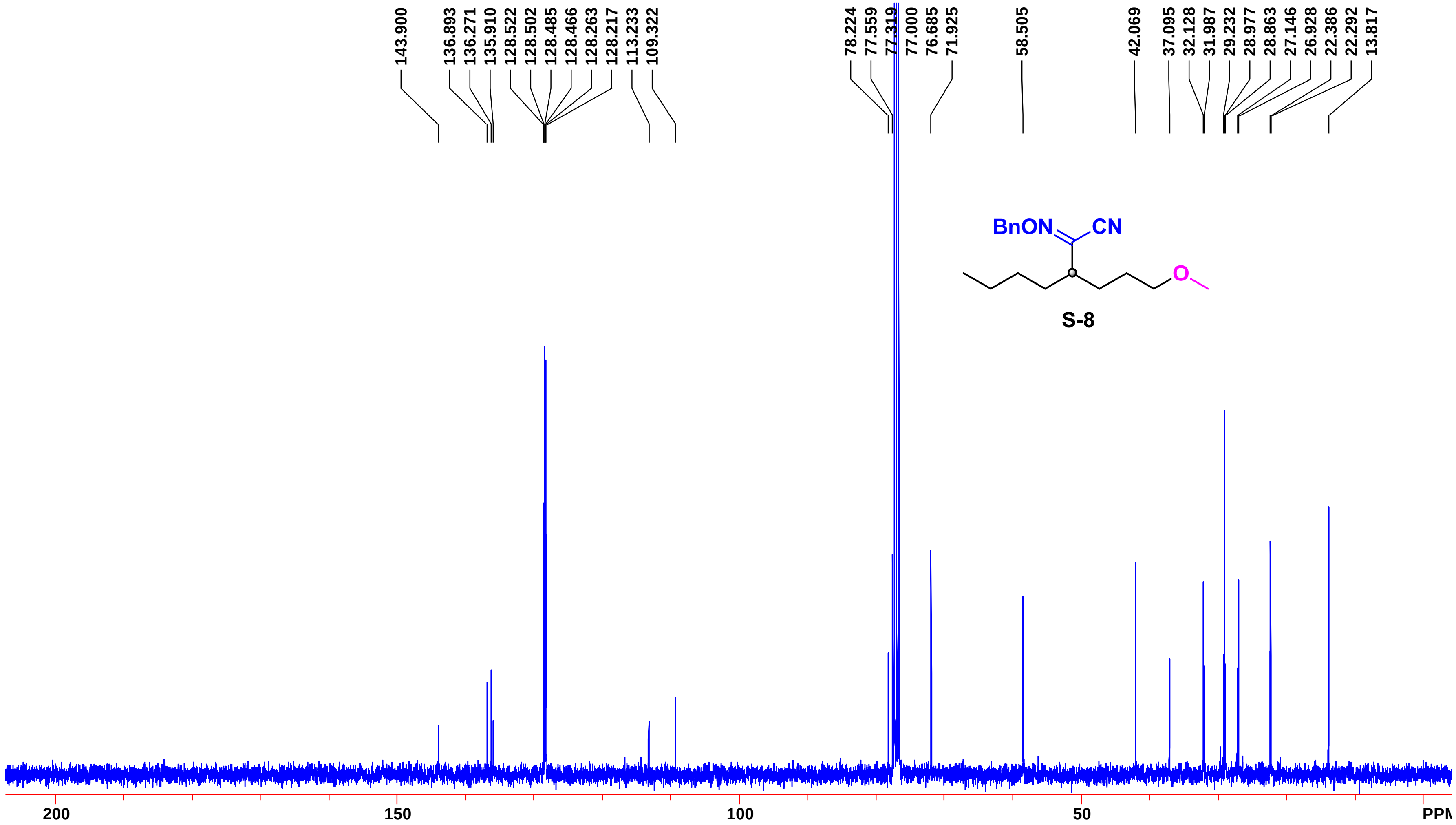

Supplementary Figure 61. ^{13}C NMR of **S-8**

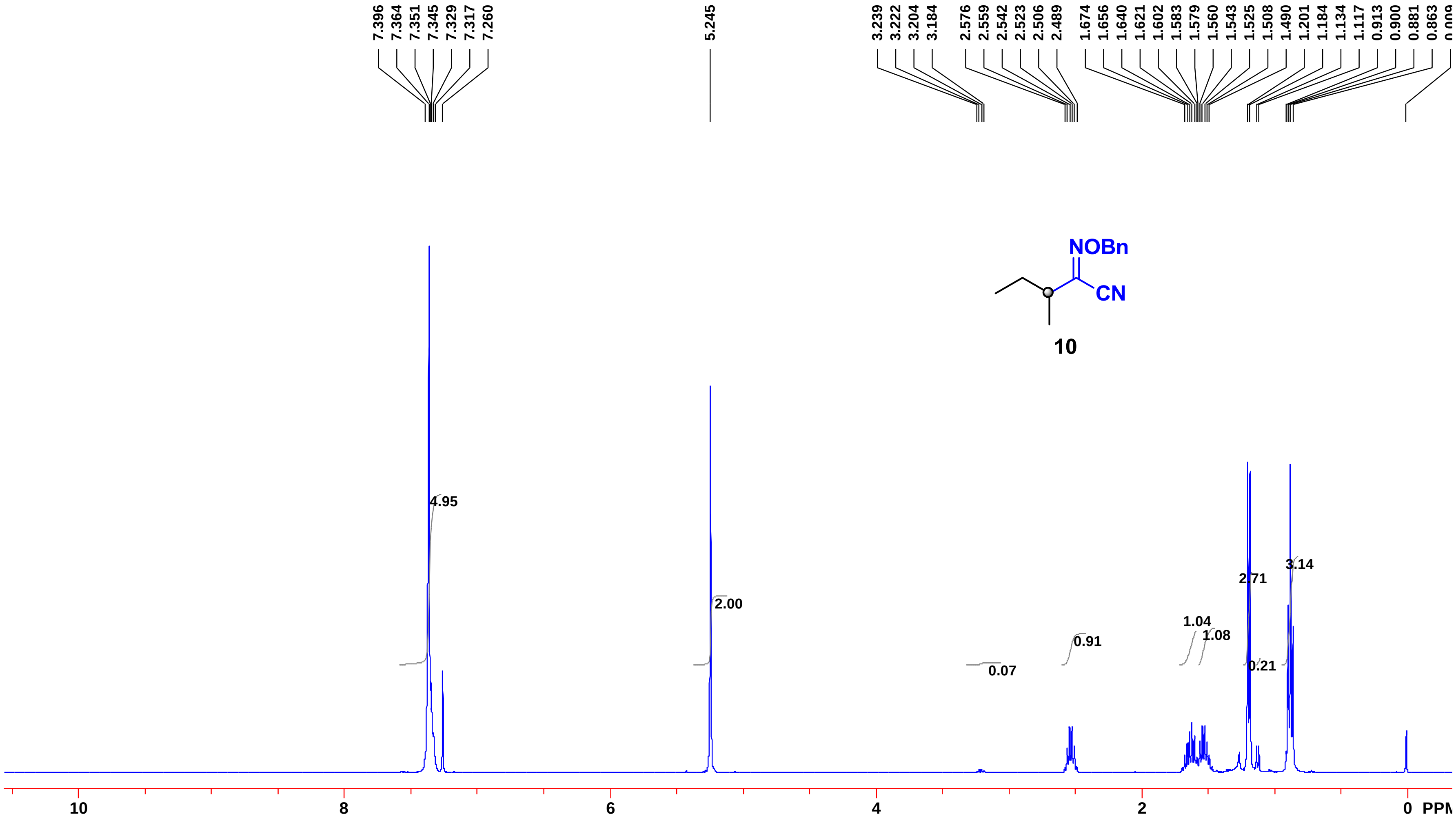

Supplementary Figure 62. ^{1}H NMR of **10**

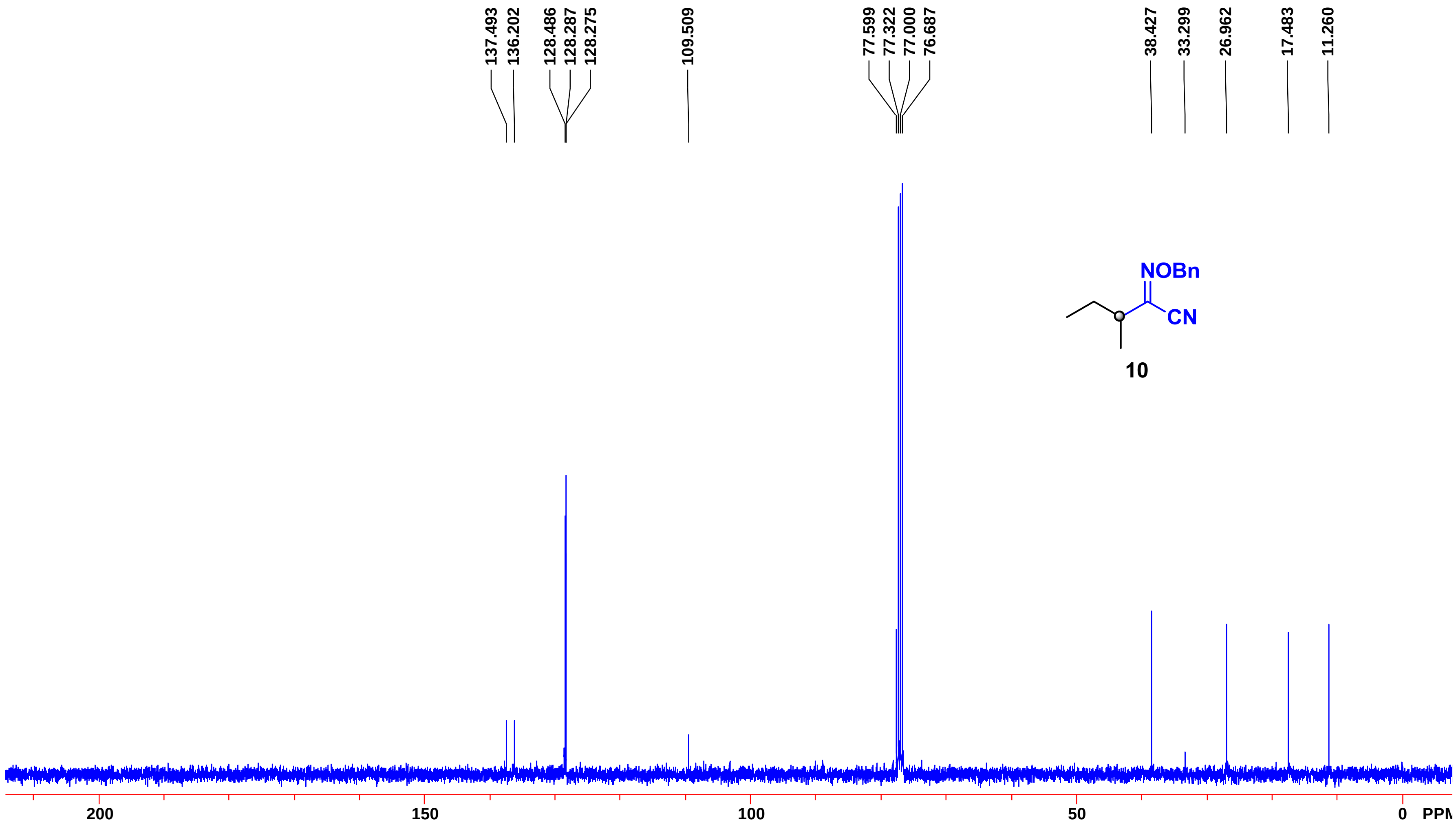

Supplementary Figure 63. ^{13}C NMR of **10**

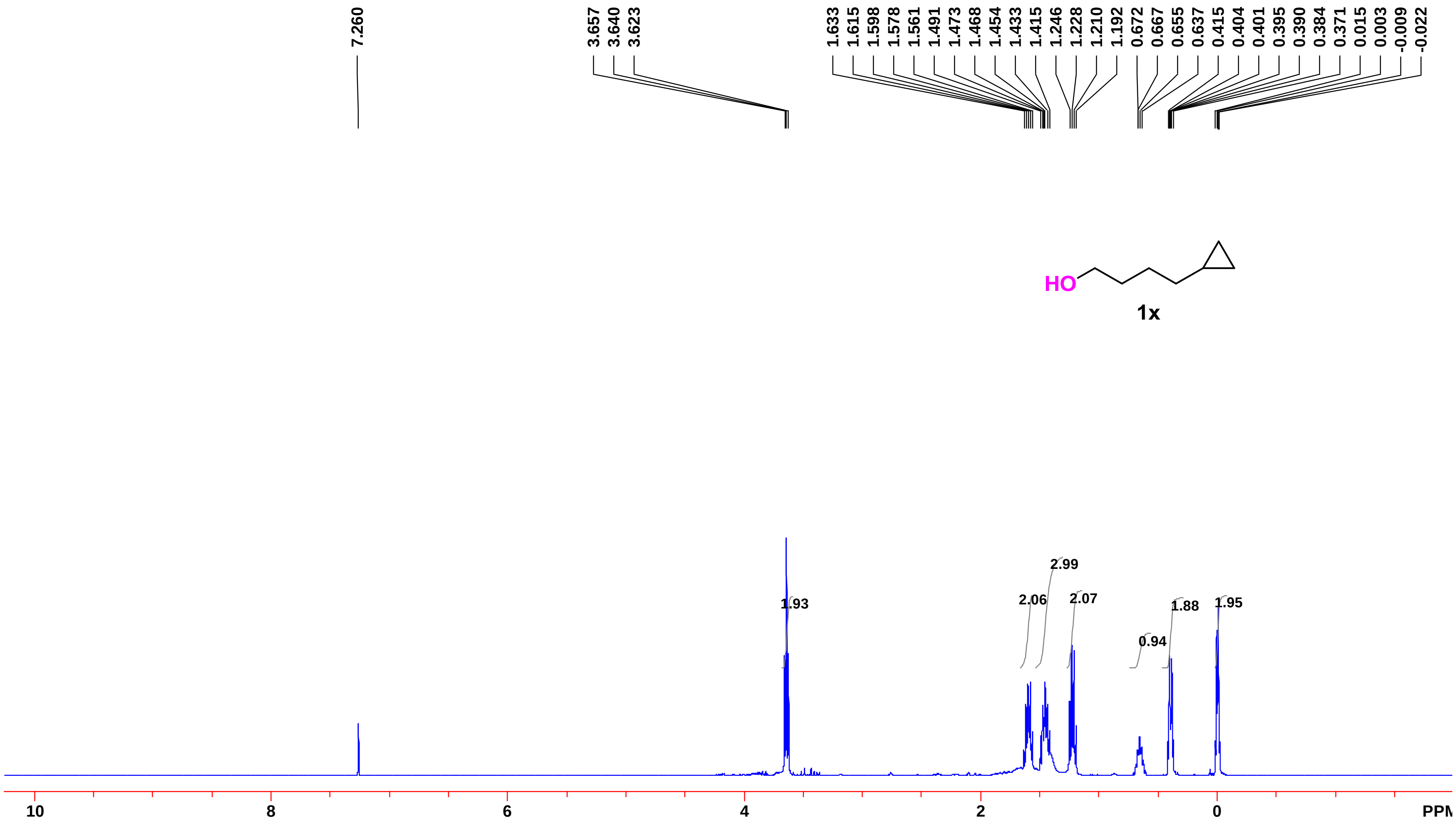

Supplementary Figure 64. ^{1}H NMR of **1x**

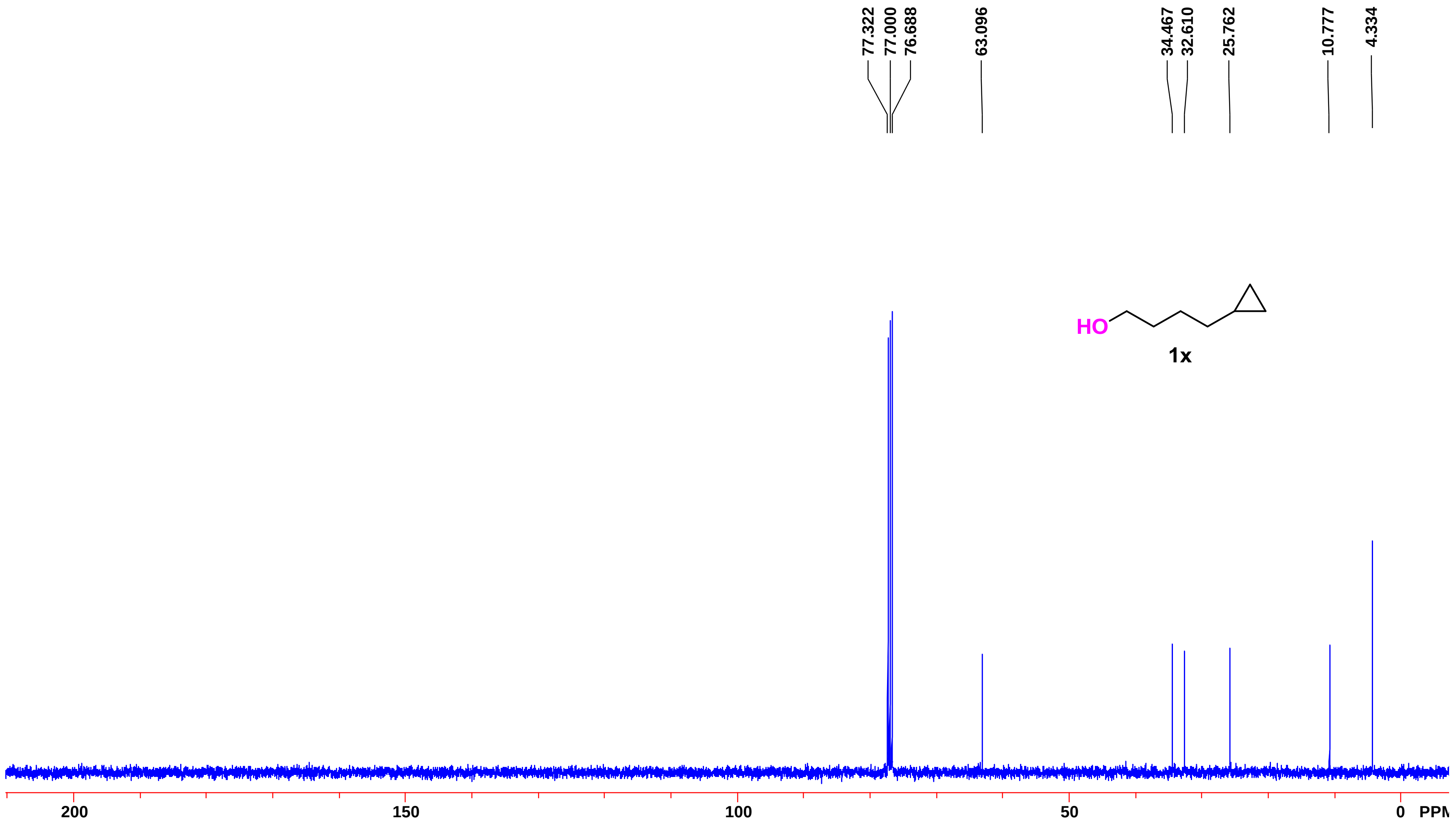

Supplementary Figure 65. ^{13}C NMR of **1x**

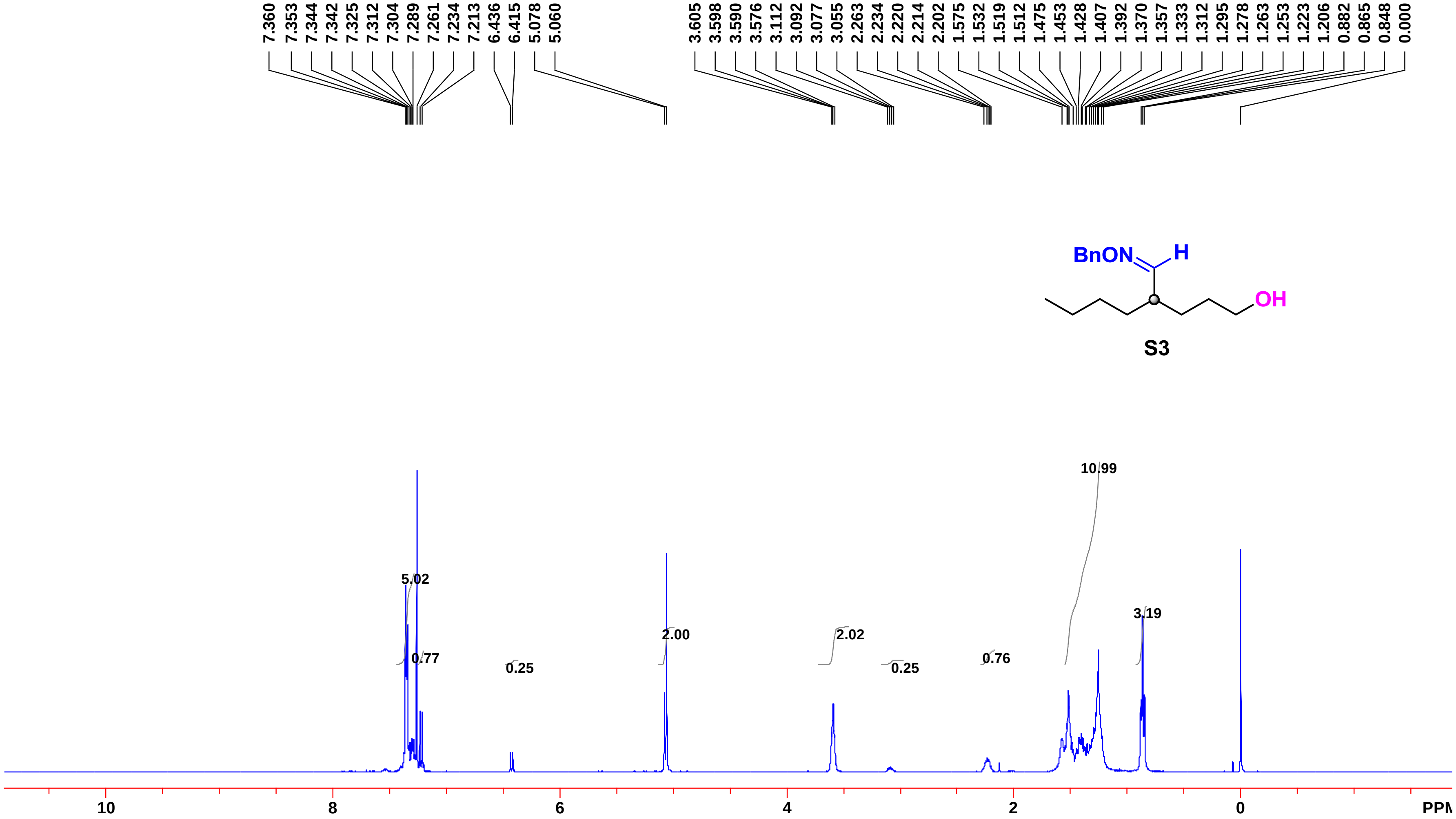

Supplementary Figure 66. ^{1}H NMR of **S3**

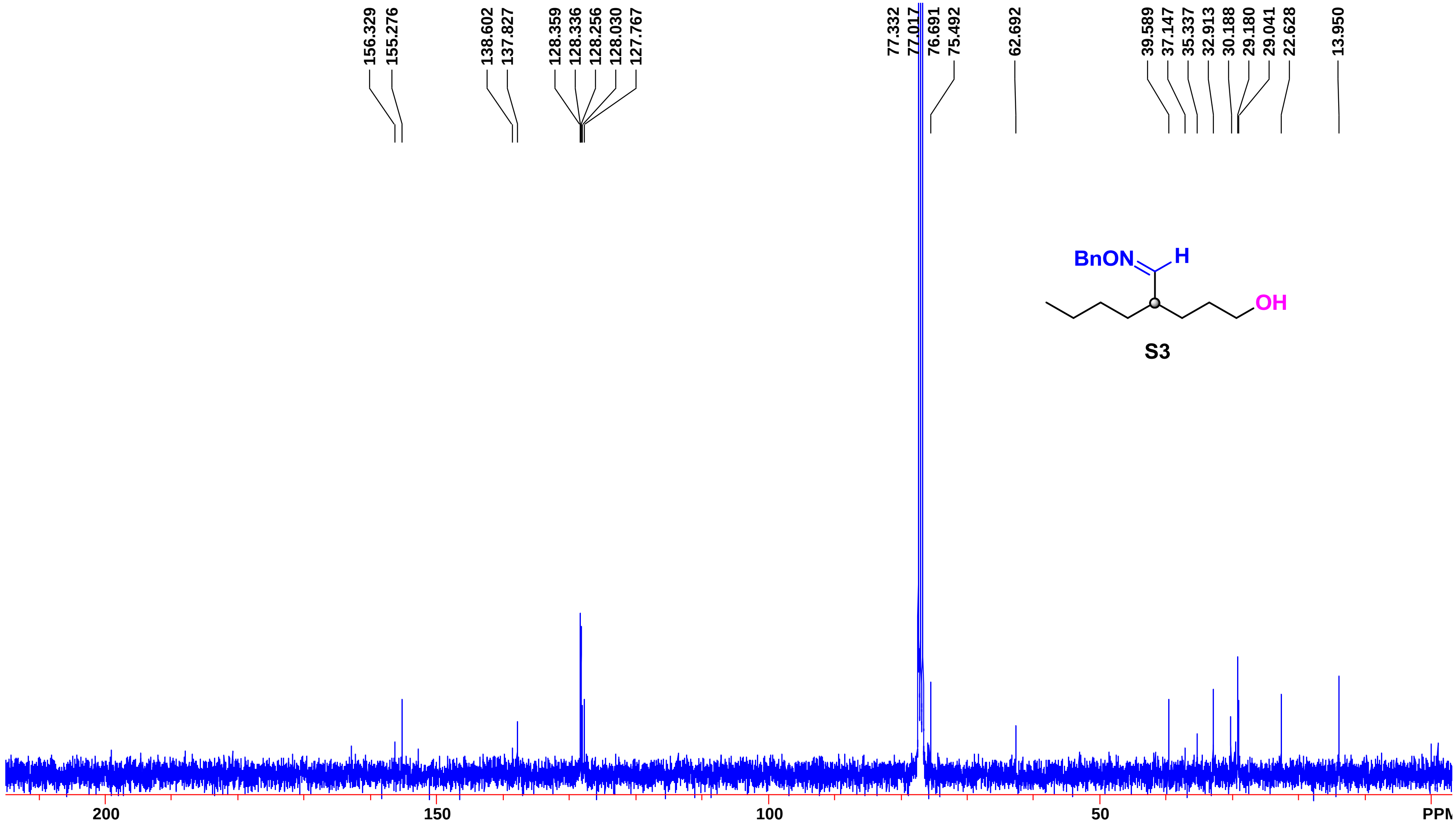

Supplementary Figure 67. ^{13}C NMR of **S3**

Supplementary References

1. Díaz, L., Casas, J., Bujons, J., Llebaria, A. & Delgado, A. New Glucocerebrosidase Inhibitors by Exploration of Chemical Diversity of N-Substituted Aminocyclitols Using Click Chemistry and in Situ Screening. *J. Med. Chem.* **54**, 2069-2079, (2011).

2. Djerassi, C., Sheehan, M. & Spangler, R. J. Mass spectrometry in structural and stereochemical problems. CCIX. Functional group interaction after electron impact. Anomalous ether cleavage in bifunctional benzyloxy ethers. *J. Org. Chem.* **36**, 3526-3532, (1971).

3. Asano, K. & Matsubara, S. Amphiphilic Organocatalyst for Schotten-Baumann-Type Tosylation of Alcohols under Organic Solvent Free Condition. *Org. Lett.* **11**, 1757-1759, (2009).

4. Melnitskii, I. A. Glukhova, O. F. Kiladze, T. K. Kantor, E. A. Rakhmankulov, D. L. & Paushkin, Ya. M. Cleavage of formaldehyde acetals by Grignard reagents. *Doklady Akademii Nauk SSSR* **292**, 1390-1392, (1987).

5. Murphy, J. A. *et al.* One-Carbon Extrusion from a Tetraazafulvalene. Isolation of Aldehydes and a Study of Their Origin. *J. Am. Chem. Soc.* **131**, 6475-6479, (2009).

6. Eliel, E. L., Nowak, B. E., Daignault, R. A. & Badding, V. G. Reductions with Metal Hydrides. XIV. Reduction of 2-Tetrahydropyranyl and 2-Tetrahydrofuranyl Ethers. *J. Org. Chem.* **30**, 2441-2447, (1965).

7. Vural, U., Durmaz, M. & Sirit, A. A novel calix[4]arene-based bifunctional squaramide organocatalyst for enantioselective Michael addition of acetylacetone to nitroolefins. *Org. Chem. Front.* **3**, 730-736, (2016).

8. Ryan, W. J., Banner, W. K., Wiley, J. L., Martin, B. R. & Razdan, R. K. Potent Anandamide Analogs: The Effect of Changing the Length and Branching of the End Pentyl Chain. *J. Med. Chem.* **40**, 3617-3625, (1997).

9. Gaspar, B. & Carreira, E. M. Cobalt Catalyzed Functionalization of Unactivated Alkenes: Regioselective Reductive C−C Bond Forming Reactions. *J. Am. Chem. Soc.* **131**, 13214-13215, (2009).

10. Landais, Y., Robert, F., Godineau, E., Huet, L. & Likhite, N. Free-radical carbo-oximation of olefins and subsequent radical-ionic cascades. *Tetrahedron* **69**, 10073-10080, (2013).

11. Kim, S., Lee, I. Y., Yoon, J. Y. & Oh, D. H. Novel radical reaction of phenylsulfonyl oxime ethers. A free radical acylation approach. *J. Am. Chem. Soc.* **118**, 5138-5139 (1996).

12. Zhu, Y., Wen, X., Song, S. & Jiao, N. Silver-Catalyzed Radical Transformation of Aliphatic Carboxylic Acids to Oxime Ethers. *ACS Catalysis* **6**, 6465-6472 (2016).

13. Mills, L. R., Barrera Arbelaez, L. M. & Rousseaux, S. A. L. Electrophilic Zinc

Homoenolates: Synthesis of Cyclopropylamines from Cyclopropanols and Amines. *J. Am. Chem. Soc.* **139**, 11357-11360, (2017).
